# Tracing the Invasion and Expansion Characteristics of the Flatid Planthopper, *Metcalfa pruinosa* (Hemiptera: Flatidae), in Korea Using Mitochondrial DNA Sequences

**DOI:** 10.3390/insects12010004

**Published:** 2020-12-23

**Authors:** Keon Hee Lee, Jun Seong Jeong, Jeong Sun Park, Min Jee Kim, Na Ra Jeong, Su Yeon Jeong, Gwan Seok Lee, Wonhoon Lee, Iksoo Kim

**Affiliations:** 1 Department of Applied Biology, Chonnam National University, Gwangju 61186, Korea; dlrjsgml0803@naver.com (K.H.L.); jjszzang1234@naver.com (J.S.J.); jungsun5009@naver.com (J.S.P.); minjeekim3@gmail.com (M.J.K.); ioveskfk@naver.com (N.R.J.); ouliais@naver.com (S.Y.J.); 2 Team of Protected Area Research, National Institute of Ecology, Seocheon 33657, Korea; 3 Experiment and Analysis Division, Honam Regional Office, Animal and Plant Quarantine Agency, Gunsan 54096, Korea; 4 Crop Protection Division, Department of Agro-Food Safety and Crop Protection, National Institute of Agricultural Sciences, RDA, Wanj 55365, Korea; gslee12@korea.kr; 5 Department of Plant Medicine and Institute of Agriculture & Life Sciences, Gyeongsang National University, Jinju 52828, Korea; wonhoon@gnu.ac.kr

**Keywords:** flatid planthopper, *Metcalfa pruinosa*, invasive species, origin, human-mediated dispersal, the A + T-rich region, population genetic analysis, multiple invasion

## Abstract

**Simple Summary:**

After the invasion of Korea in 2005, the first lines of studies on *Metcalfa pruinosa* inferred the origin of the species using a fragment of mitochondrial *COI* sequences. However, the low variability of the sequences limited further scrutinized inference on the invasion dynamics. In this study, we sequenced a fragment of the *COI* gene from 536 individuals of the species and combined the sequence data with the available GenBank data, totaling 830 individuals. These data indicated that the North-West region is a point of entry in addition to the South-East region, the presumed sole point of entry to Korea. Furthermore, it suggested that North-West entry involves the *M. pruinosa* originating from the USA. In an effort to find further variable regions in the mitochondrial genome, one region provided substantially increased variability compared to the fragment of the *COI*. The concatenated sequences of *COI* and the newly obtained variable region, which were used to infer the expansion pattern in Korea, indicated that the main highway, running obliquely between the North-West and South-East regions, appears to be responsible for the current population genetic structure of *M. pruinosa* in Korea, facilitating gene flow through this highway traffic.

**Abstract:**

The flatid planthopper, *Metcalfa pruinosa* (Hemiptera: Flatidae), which is an invasive species, is widespread in Korea. We sequenced a fragment of the *COI* from 536 individuals collected mainly in Korea and the European countries and combined these sequence data with the public data, totaling 830 individuals worldwide. The identification of one shared haplotype only between Korea and the USA, the presence of this haplotype only in the North-West region of Korea, and the highest haplotype diversity in this region suggested that the North-West region is another point of entry in addition to the South-East region, which is the presumed sole point of entry to Korea. Furthermore, it suggested that North-West entry involves the *M. pruinosa* originating from the USA. In an effort to find further variable regions in the mitochondrial genome, one region provided substantially increased variability compared to that of the fragment of *COI*. *F*_ST_ estimation, PCoA, and BAPS analysis, using the concatenated sequences of *COI* and the newly detected variable region to infer the expansion pattern in Korea, indicates that the main highway, running obliquely between the North-West and South-East regions, appears to be responsible for the current population genetic structure of *M. pruinosa* in Korea, facilitating gene flow through this highway traffic.

## 1. Introduction

The flatid planthopper, *Metcalfa pruinosa* (Say) (Hemiptera: Flatidae), which is native to North America, including Mexico and Cuba [1,2], was accidentally introduced to north-eastern Italy in 1979 [3]. It spread to all southern, central, and eastern European countries; Russia; and Australia [4]; and was also reported in Korea in 2005 [5].

*Metcalfa pruinosa* is univoltine and overwinters as eggs buried in the bark of a range of host plants both in the USA [1,6] and Europe [7]. In Korea, the species also overwinters as eggs buried in the bark; the nymphs occur between the end of May and the middle of July, and the adults occur between the middle of July and late October [8]. *M. pruinosa* causes economic and aesthetic damage to approximately 100–300 species of crops, woody plants, and ornamental vegetation in the USA, Europe, and Korea, by feeding on phloem sap, secreting wax and honeydew, inhibiting transpiration and subsequently forming sooty mold [8,9,10,11,12,13,14]. In Italy, a yield loss of 30–40% of the soybean crop was reported [15].

Previously, the genetic magnitude and divergence of *M. pruinosa* populations were investigated to trace the origin of the Korean populations [16,17]. Park et al. [17] analyzed a 472-bp fragment of the DNA barcoding region of samples collected mainly from Korea, four European countries, and those native to the USA, along with a few GenBank-registered sequences from Canada [18]. They detected 19 haplotypes, which showed 0.21% (1 bp) to 2.54% (12 bp) divergence, presenting mid-to-high intraspecific divergence compared to that in other insect species [19]. However, 162 Korean samples collected from nationwide regions revealed only three haplotypes (H1, H3, and H9), which diverged by a maximum of only 0.64% (3 bp). One haplotype (H1) was dominant in Korea (94%), the majority of European countries (72%), and the USA (50%), representing 69.8% of the worldwide samples [17]. Similarly, using a 577-bp fragment of the DNA barcoding region from the 42 samples collected in Korea, Kwon et al. [16] found only two haplotypes (Hap_01 and Hap_02), with the frequency of Hap_01 at 95%. These previous studies analyzed the sequence data from several perspectives, including haplotype relationships and pairwise *F*_ST_, to trace the origin of the Korean populations [16,17]. Consequently, either Europe or both the USA and Europe have been ascribed as the sources of introduction to Korea.

Although a considerable amount of time has elapsed since the first record of *M. pruinosa* in Korea, it is important to accumulate more knowledge on its invasion history for several quarantine aspects such as the origin of invasion, the point of entry, an expansion pattern, and additional invasion. Furthermore, the low number of haplotypes and sequence divergence of the *COI* sequences recorded thus far for the Korean populations might be a limiting factor for a thorough interpretation of such aspects of invasion. Thus, other DNA segments that have a higher divergence than the *COI* sequences would be necessary. Recently, we sequenced two mitochondrial genomes (mitogenomes) of *M. pruinosa*, each from a different individual, which possessed different *COI*-based haplotypes and comparison of the two mitogenomes provided several variable regions [20]. After the preliminary test using several representative individuals originating from different locations, one region that had a higher number of haplotypes, sequence divergence, and variable sites than those of the DNA barcoding region was selected.

In this study, we first sequenced a 658-bp fragment of the mitochondrial *COI*, corresponding to the DNA barcoding region, from more than 500 individuals collected mainly from Korea and a few from three European countries. These sequences were combined with those from worldwide populations via a GenBank search (a total of 830 individuals from 50 localities in seven countries, including Korea) to identify any additional haplotype, which might be helpful to infer the connection between the Korean and the potential source populations. In addition, a newly discovered variable mitochondrial DNA segment located in the A + T-rich region was sequenced for 342 individuals collected mainly from Korea and a few from three European countries. Finally, these sequences were concatenated with the DNA barcoding region to examine within-country genetic relationships, population structure, and genetic diversity to understand the expansion pattern in Korea.

## 2. Materials and Methods

### 2.1. Sample Collection and DNA Extraction

A total of 536 individual adults or nymphs of *M. pruinosa* were collected from 31 localities, including 23 in Korea, 4 in Italy, 3 in France, and 1 in Spain, during several field trips from 2011–2018 (Table S1). The majority of the samples collected in Korea were stored at −70 °C immediately after transportation to the laboratory, whereas those from foreign countries were preserved in 95% ethanol until molecular experiments were performed. Total DNA was extracted from one or two legs of the adults and the whole body of both the nymph and the old ethanol-preserved samples using the Wizard Genomic DNA Purification Kit, in accordance with the manufacturer’s instructions (Promega, Madison, WI, USA).

### 2.2. Primers, PCR, and Sequencing

For amplification of the 658 bp fragment of the DNA barcoding region, a pair of primers was adapted from Folmer et al. [21]; however, the reverse primer was later replaced with the species-specific one designed in this study using complete mitogenome sequences to increase amplification efficiency [20]. The primer sequences are as follows: LCO1490, 5′-GGTCAA CAAATCATAAAGATATTGG-3′ [21] and MPCOIR2, 5′-AGAATATAAACTTCTGGGTGA-3′ (designed in this study).

To identify variable regions in addition to the DNA barcoding region, two complete mitogenome sequences of *M. pruinosa*, each of which differed in their DNA barcoding region [20] (collected from Gimhae, Gyeongsangnam-do Province, Republic of Korea and Montpellier in France, respectively), were compared. The two genomes showed a sequence divergence of only 0.061% in the whole genome, 0.064% (seven sites) in the protein-coding genes, including the DNA barcoding region, and 0.168% (three sites) in the A + T-rich region. Based on this information, primers were designed from the five variable regions (Table S2), and a few individuals were sequenced for these regions before we selected the final region. Among them, unfortunately, only one region (Region 5), which is located in the A + T-rich region, provided higher variability than the DNA barcoding region in terms of variable nucleotide positions (three vs. nine positions), the number of haplotypes (two vs. five), and percentage sequence divergence (0.168% vs. 0.503%) when several representative individuals originating from different regions were used (data not shown). Thus, Region 5 was selected finally. The primers for Region 5 were designed to amplify the 424–428 bp fragment of the A + T-rich region, which is located close to the 5-end of the *tRNA^Ile^* in the 1788-bp long A + T-rich region of *M. pruinosa* [20]. The primer sequences mainly used are as follows (Table S2): Region5F1, 5′-ATTAATTAAAATGCGTTGATC-3′ for forward amplification and Region5R1, 5′-CAATATATAATCTAAGTTATAC-3′ for reverse amplification. Region 5 was sequenced for 342 individuals, comprising 16 populations from among the 23 Korean populations and all foreign populations (eight populations from three countries, Italy, Spain, and France) that were sequenced for the DNA barcoding region (Table S1).

PCR amplification was performed under the following conditions: an initial denaturation step at 94 °C for 4 min, 35 amplification cycles (denaturation at 94 °C for 1 min, annealing at 48–52 °C for 1 min for each DNA barcoding region and 50–54 °C for Region 5, and extension at 72 °C for 1 min), and a final extension step at 72 °C for 7 min using the AccuPower^®^ PCR PreMix (Bioneer, Daejeon, Korea), which contains 1 U of Top DNA polymerase, both directional primers, and the template DNA in a reaction volume of 20 µL. The PCR products were then purified using an AccuPrep^®^ PCR purification kit (Bioneer, Seoul, Korea). Electrophoresis was performed in 1× Tris-acetate EDTA buffer on 1% agarose gel to confirm successful DNA amplification. DNA sequencing was performed using an ABI PRISM^®^ BigDye^®^ Terminator v. 3.1 Cycle Sequencing Kit with an ABI 3100 Genetic Analyzer (PE Applied Biosystems, Foster City, CA, USA). All the PCR products were sequenced in both forward and reverse directions.

### 2.3. Sequence Analysis

The sequences of both strands from each individual were aligned using Clustal Omega [22] (http://www.ebi.ac.uk/Tools/msa/clustalo) to obtain a finalized sequence for each individual and each sequence region. When homologous sequences from two individuals differed by ≥ one nucleotide or an insertion/deletion (indel), the sequences were considered to be different haplotypes. Unordered pairwise comparisons between haplotypes were performed using PAUP ver. 4.0b [23]. Nucleotide sequences for the DNA barcoding region were translated based on the invertebrate mitochondrial DNA genetic code to check for the presence of any pseudogene sequences. Haplotype designations were applied to the new sequences as they were discovered (i.e., MPBAR01, MPBAR02, and MPBAR03 for DNA barcoding sequences (658 bp); MPR501, MPR502, and MPR503, and so forth for Region 5 (424–428 bp); and MPBR01, MPBR02, MPBR03, and so forth for the concatenated sequences of the DNA barcoding region and Region 5 (1082–1086 bp)).

### 2.4. GenBank Data Search

The preexisting DNA barcoding sequences of 294 individuals from seven countries, including Korea, referring to previous studies as of June 2019, were downloaded from the GenBank [16,17,18,24] (Table S3). These comprised samples originated from 38 locations in seven countries (Korea, USA, Canada, Italy, Spain, France, and Slovenia). Several sequences were excluded owing to a shorter length, ambiguity as *M. pruinosa*, possible pseudogene sequences, and redundancy. The finalized 294 sequences were combined with the 536 currently obtained individual sequences, resulting in a total of 830 individual sequences originating from 50 localities in seven countries (Figure S1) and the overlapping 470 bp fragment of *COI* sequences was used for subsequent analyses, which are specified in the Section 3.2. Haplotypes were named MPH01, MPH02, MPH03, and so forth.

### 2.5. Genetic Diversity

Using worldwide *COI* data, genetic diversity estimates, including haplotype diversity and nucleotide diversity, were obtained based on each country using Arlequin ver. 3.5 (Excoffier and Lischer 2010). This analysis was performed for each country, which was represented by ≥ two haplotypes according to the method proposed by Nei [25]. The maximum sequence divergence within each country was obtained by extracting the within-population estimates of unrooted pairwise distances from PAUP [23].

Within Korea, the localities with a relatively higher number of haplotypes were scattered throughout the Korean regions in the preliminary analysis when the concatenated sequences of the DNA barcoding region and Region 5 were used. Thus, a thorough examination and interpretation of the diversity distribution were difficult. Therefore, for the concatenated sequences, genetic diversity estimates were obtained primarily based on each country, but those for the Korean populations were additionally obtained by subdividing the 23 Korean populations into four Korean regions by geographic affinity to examine the expansion pattern of *M. pruinosa* within Korea (North-West, North-East, South-West, and South-East regions).

### 2.6. Structure

Genetic distance (and migration rate) was estimated from subroutines in Arlequin ver. 3.5 [26]. Population pairwise genetic distances (*F*_ST_) and a permutation test of the significant differentiation (1000 bootstraps) of the pairs of countries for worldwide *COI* data and countries/Korean regions for concatenated sequences, were obtained following the approach described by Excoffier et al. [27]. The distances between the DNA sequences were calculated using the Kimura two-parameters method [28]. Pairwise *F*_ST_ values were used to estimate per generation migration rate, *N_m_* (the product of the effective population size, *N_e_*, and the migration rate, *m*), based on the equilibrium relationship: *F*_ST_ = 1/(2*N_m_* + 1). The degree of population differentiation was visualized as a heat map using Python 3.5.2, seaborn 0.7.1, numpy 1.12.0, and pandas 0.19.2 (Python Software Foundation, Beaverton, OR, USA). Furthermore, principal coordinate analysis (PCoA) [29] was performed using the pairwise *F*_ST_ obtained from Arlequin ver. 3.5 [26] to detect and plot the relationships among populations for worldwide *COI* data and countries/Korean regions for concatenated sequence data using GenAlEx ver. 6.5 with default parameters [30]. The genetic structure of *M. pruinosa* among countries for worldwide *COI* data and among countries/Korean regions for concatenated sequences was further analyzed using Bayesian Analysis of Population Structure (BAPS) ver. 6.0 [31]. The analysis was performed using clustering, with a linked locus module and a codon model. In this process, mixture analysis was performed with *K*-values ranging from 1–10, and optimal clusters were identified based on the maximum log marginal likelihood values.

## 3. Results

### 3.1. DNA Barcoding Region

The analysis of the DNA barcoding region (658 bp) for 536 individuals collected from Korea and three European countries (Italy, France, and Spain) revealed only three haplotypes (MPBAR01, MPBAR02, and MPBAR03), with a maximum sequence divergence of 0.46% (3 bp; between MPBAR01 and MPBAR03). Two haplotypes were found in both Italy and France (MPBAR01 and MPBAR02), MPBAR01 was found in only Spain, and all three haplotypes were recorded in Korea (Table S4). In Korea, MPBAR01 was found in all 23 populations (94.29%), MPBAR02 was found in 11 populations (5.5%), and MPBAR03 was found as a single individual (0.2%) in a population located in the North-West region (locality 4, Yeoju; Figure S1; Table S4). In the European countries, the frequency of MPBAR01 was 68.89% (31 individuals) and that of MPBAR02 was 31.11% (14 individuals), thus indicating a difference in the haplotype frequency between Korea and the European countries.

### 3.2. Worldwide COI Sequence Analysis

#### 3.2.1. Haplotype Distribution

**Figure 1:**
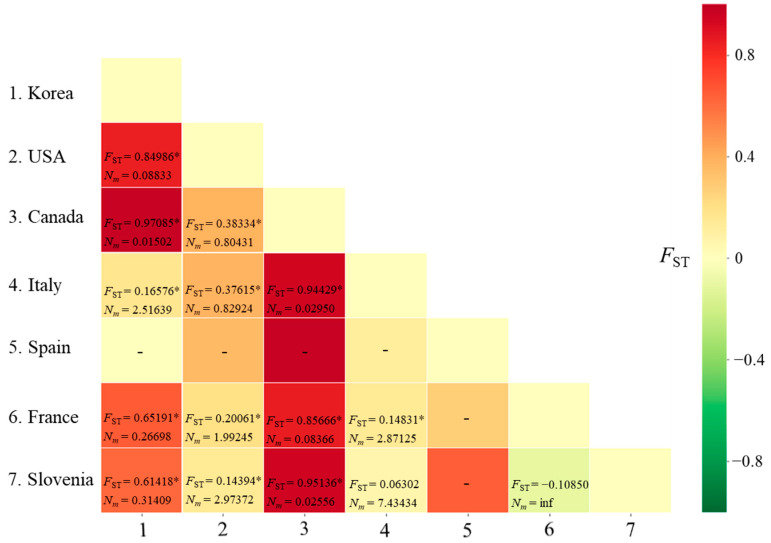
Matrix of genetic distances (*F*_ST_) and migration rate (*N_m_*) between pairs of countries for *Metcalfa pruinosa* using the worldwide *COI* sequences. * *p* < 0.05. inf, infinite. −, not available owing to a single haplotype in Spain.

The addition of the newly sequenced Korean (491 individuals) and European (45 individuals) samples to the GenBank data collection (294 individuals) led to the detection of only one additional haplotype (MPH20), which was found in Canada as a single individual (Unpublished, GenBank accession number MF932798) (Table 1; Table S3). In particular, no single new haplotype was detected in Korea. Thus, only a single haplotype was added after the report by Park et al. [17]. Nevertheless, we were able to download four additional individuals that have MPH03 (MPBAR03 for the 658-bp barcoding region) from the GenBank data, which were originally reported in Park et al. [17], in addition to a single individual detected in the current study. This haplotype was not highlighted in Park et al. [17]; however, it signifies that the four individuals possessing this haplotype were all found in a North-West locality in Korea (locality 4, Yeoju; Figure 1) and in the USA (Table 1). Among the 830 individuals, MPH01 was represented by 674 individuals (81.2%) and the next highest frequency was that of MPH02 by 54 individuals (6.50%) (Table 1). These two haplotypes are the most extensively shared in all the introduced countries, although MPH02 was not detected in Spain.

#### 3.2.2. Genetic Diversity

The estimation of within-country diversity using the worldwide *COI* sequences showed that haplotype diversity (*H*, max = 1.0) was lower in some countries invaded by *M. pruinosa*, such as Korea (0.1068) and Spain (not applicable owing to a single haplotype), whereas it was moderate in France (0.6381), Slovenia (0.6667), and Italy (0.3528) (Table S5). The USA, which is the most likely source of introduction among the native countries, was the highest in haplotype diversity (0.9413), whereas that of another native country, Canada, was low at 0.1215. Nucleotide diversity (π) per country also showed a pattern similar to that of *H*; the highest π was in the USA (0.020395); and the lowest π was in Spain (0), with a relatively lower estimate in Korea (0.000477).

#### 3.2.3. Genetic Distance and Structure

The *F*_ST_ between pairs of countries ranged from −0.10850 (between France and Slovenia) to 0.97085 (between Korea and Canada), with a range of *N_m_* from infinite (between France and Slovenia) to 0.01502 (between Korea and Canada) (Figure 1). A non-significant genetic differentiation at the level of *p* < 0.05 was observed only in the comparisons between Italy and Slovenia and between France and Slovenia.

**Figure 2:**
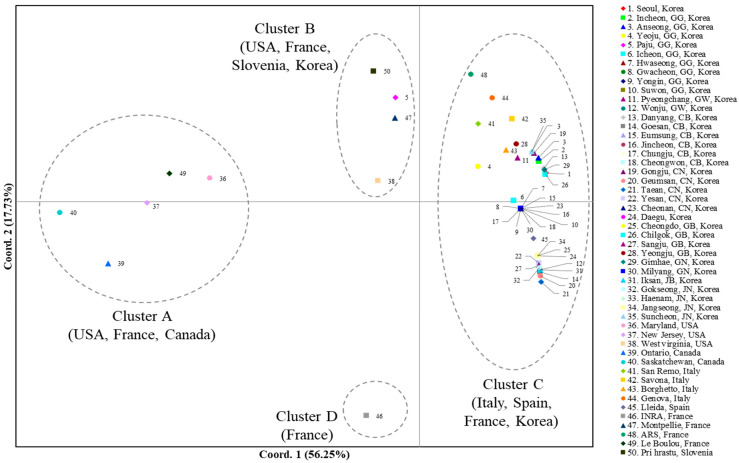
Results of the principal coordinate analysis (PCoA) based on collection localities using the worldwide *COI* data. The percentage variation explained by the first and second axes is indicated.

A PCoA using the worldwide *COI* data to scrutinize population relationships showed that the first and second components accounted for 56.25% and 17.73%, respectively, of the variation (Figure 2). Overall, four clusters (Clusters A, B, C, and D) that could be differentiated based on the two components were detected, although one cluster that stood alone for one French population (Cluster D) was differentiated only by the second component. Cluster A comprised two populations each from Canada (localities 39 and 40, Ontario and Saskatchewan, respectively) and the USA (localities 36 and 37, Maryland and New Jersey, respectively) and one from France, suggesting that one population in France is genetically close to a few North American populations. Cluster B comprised one population each from Slovenia (locality 50, Pri Hrastu), Korea (locality 5, Paju), France (locality 47, Montpellier), and the USA (locality 38, West Virginia), indicating a mixture of the populations from the USA, European countries (Slovenia and France), and Korea. Finally, Cluster C comprised all the populations from Korea (excluding locality 5, Paju), all from Italy, one from Spain (locality 45, Lleida), and one from France (locality 48, ARS). These results collectively indicate that the majority of the Korean populations are closer to the European populations, such as Italy, Spain, and France, rather than to the North American populations, but one USA population indicates genetic closeness to Korea (Cluster B).

BAPS analysis using the worldwide *COI* data to examine the likelihood scores from 10 replicate runs across *K*-values ranging from 1 to 10, indicated that the optimal *K*-value was seven, suggesting that the *M. pruinosa* individuals comprise seven haplotype clusters (hereafter referred to as haplogroups; Figure S2). The assignment results of *K* = 7 showed that all the seven haplogroups were found in the USA with varying degrees of frequency; moreover, we found three in France; two each in Canada, Korea, Italy, and Slovenia; and one in Spain. The USA, which showed the highest number of haplotypes (16), shared two entire haplogroups with those found in Korea (sky-blue and red), and all other countries also shared entire haplogroups with the USA. Among the three haplogroups, two entire haplogroups (sky-blue and red) found in Korea completely corresponded to haplogroups of both Italy and Slovenia (red and sky-blue), whereas only one haplogroup corresponded to that of Spain (sky-blue) and two corresponded to those of France (red and sky-blue). This haplogroup analysis indicates that the Korean populations have a certain level of genetic relationships with those of both the USA and all European countries, by sharing at least one haplogroup with the other countries.

Considering the analysis of worldwide *COI* data including haplotype distribution, PCoA, and BAPS together, the Korean populations have a closer genetic affinity to European countries; however, a certain level of genetic closeness to the USA is also detectable, in particular considering the finding of MPH03 only in the USA and in a Korean population (locality 4), the clustering of one USA population (West Virginia) together with one Korean population (locality 5) in PCoA, and the sharing of all Korean haplogroups with those of the USA in the BAPS analysis.

### 3.3. Variability in Region 5

To examine the domestic expansion pattern in Korea, a variable region was developed from two mitogenome sequences of *M. pruinosa* [20; GenBank accession numbers MK303326 and MN417319]. A preliminary test using a few geographic samples of *M. pruinosa* has shown that one region in the A + T-rich region (Region 5) has higher variability than that of the DNA barcoding region. The primers designed from these mitogenome sequences (Table S2) successfully amplified and sequenced Region 5 from 342 individuals (Table S1).

A total of 15 haplotypes were detected with a maximum sequence divergence of 2.34% (10 bp; Table S6). Twelve haplotypes were found in Korea, three in both Italy and Spain, and six in France (Table S7). Eight of the 15 haplotypes were unique to Korea, 0 in Italy, 1 in Spain, and 2 in France (Table S7). Among the 12 haplotypes found in Korea, MPR501, MPR502, and MPR503 were found in 14, 12, and 6 localities, respectively, whereas others were found in 1–3 localities, with MPR507, MPR511, and MPR512 represented by a single individual in a locality (Table S1).

### 3.4. Concatenated Sequences of the DNA Barcoding Region and Region 5

#### 3.4.1. Sequence Analysis

Comparison of the two mitochondrial regions showed that Region 5 provided higher variability than the DNA barcoding region in terms of variable nucleotide positions (14 vs. 3 positions, respectively), number of haplotypes (15 vs. 3, respectively), maximum sequence divergence (2.34% vs. 0.46%, respectively), number of populations with two or more haplotypes (20 vs. 13, respectively), and haplotype diversity (0.6504 vs. 0.1450, respectively) (Table S8). When the two sequence regions were concatenated (1082–1086 bp), the variable positions increased to 17, haplotype number to 20 (MPBR01–MPBR20), and nucleotide diversity to 0.6732 (Table S8). The 17 variable positions comprised 14 nucleotide substitutions, which consisted of 10 transitions (each seven T↔C and three G↔A) and 4 transversions (each two A↔C and A↔T) and 3 indels, but no amino acid substitution was invoked (Table S9).

#### 3.4.2. Haplotype Distribution

**Figure 3:**
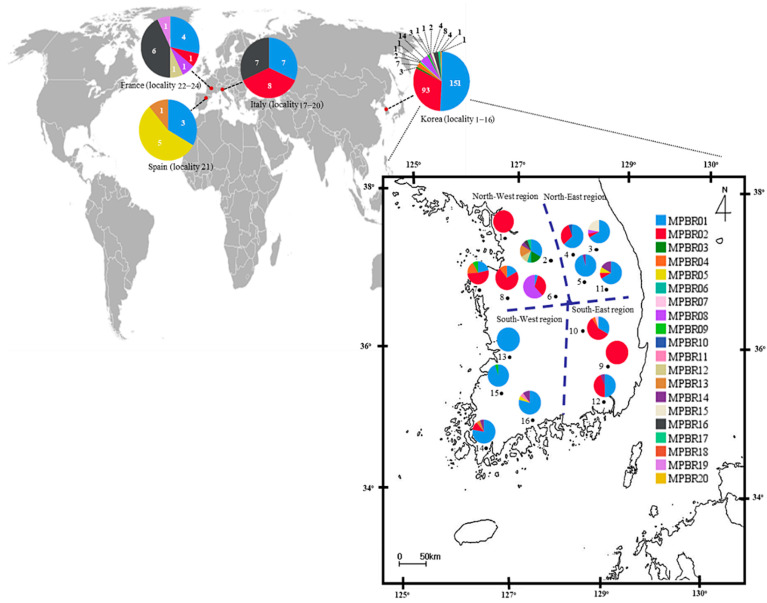
Distribution of the haplotypes defined based on the concatenated sequences of 658 bp fragments of the *COI* and Region 5 of *Metcalfa pruinosa*. The general locality names are as follows: 1, Incheon (MPBR02, 15 individuals); 2, Yeoju (03, 3; 06, 1; 14, 2; 15, 3; 16, 1; 18, 1; and 20, 1); 3, Pyeongchang (01, 12; 02, 1; 08, 1; and 17, 4); 4, Wonju (01, 12; 02, 6; and 10, 1); 5, Danyang (01, 21; and 16, 1); 6, Goesan (01, 1; 02, 7; and 08, 13); 7, Taean (01, 5; 02, 12; 04, 4; and 09, 2); 8, Yesan (01, 3; 02, 13; and 04, 2); 9, Cheongdo (02, 17); 10, Sangju (01, 7; 02, 12; 04, 1; and 07, 1); 11, Yeongju (01, 8; 02, 1; 05, 1; and 16, 2); 12, Gimhae (01, 8; 02, 7; and 16, 1); 13, Iksan (01, 20); 14, Haenam (01, 14; 02, 2; 15, 1; and 16, 1); 15, Jangseong (01, 19; and 09, 1); 16, Suncheon (01, 15; 05, 1; 11, 1; and 16, 2); 17−20, Italy (01, 7; 02, 8; and 16, 7); 21, Spain (01, 3; 05, 5; and 13, 1); and 22−24, France (01, 4; 02, 1; 08, 1; 12, 1; 16, 6; and 19, 1). The dotted lines on the map of Korea indicate the division into four regions for the population genetic analyses.

Among the 20 haplotypes, MPRB01 and MPRB02 each accounted for 48.25% (166 individuals) and 29.83% (102 individuals), respectively (Table S10; Figure 3). In the European countries, however, the frequency of MPRB01 and MPRB02 was 31.11% (14 in 45 individuals) and 20.0% (9 in 45 individuals), respectively, totaling 51.11%, whereas these two haplotypes in Korea accounted for 82.16% (244 in 297 individuals). MPBR16, which was detected at a frequency of 2.69% (eight individuals) in Korea, was detected as the haplotype with the second highest frequency at 28.89% in Europe (13 individuals), presenting a somewhat different frequency among the dominant haplotypes between Korea and Europe.

Among the 17 haplotypes found in Korea, MPBR01, MPBR02, and MPBR16 were the most widely distributed in 14, 11, and 6 localities, respectively; others were found in 1–3 localities (Figure 3; Table S11). Haplotype abundance was exceptionally high at eight in locality 2 (Yeoju), which is located in the North-West region, compared to that at any of the other localities in Korea and the next highest was four at localities 3, 7, 10, 11, 14, and 16 (Pyeongchang, Taean, Sangju, Yeongju, Haenam, and Suncheon, respectively), presenting a trend that the localities with the higher number of haplotypes are scattered throughout the Korean region (Figure 3; Table S11).

#### 3.4.3. Genetic Diversity

The estimation of within-country diversity using concatenated sequences indicated mid-to-high *H* (max = 1.0) and π per country, ranging from 0.6389 (Spain) to 0.7692 (France) in *H* and 0.000924 (Spain) to 0.004968 (France) in π, indicating that Spain was the lowest and France was the highest, although there was no substantial difference in both *H* and π among countries when standard errors were considered (Table 2). Korea ranked third, next to Italy, in both *H* and π, although the number of haplotypes, which may have been affected by the larger sample size, was the highest at 17.

The Korean region-based analysis, such as North-West (localities 1, 2, 6, 7, and 8), North-East (localities 3, 4, 5, and 11), South-West (localities 13, 14, 15, and 16), and South-East regions (localities 9, 10, and 12; Figure 3), indicates that *H* ranges from 0.2201 (South-West) to 0.7118 (North-West), and π from 0.000933 (South-West) to 0.002217 (North-West), with substantially higher *H* in the North-West region and a substantially lower *H* in the South-West region, but the differences observed in π were not substantial. This regional subdivision further indicates that the *H* in the North-West region is the second highest, next to that of France (Table 2), suggesting that the region-based analysis is informative and that there is an obvious regional difference in *H*, thereby necessitating an additional explanation.

#### 3.4.4. Genetic Distance

**Figure 4:**
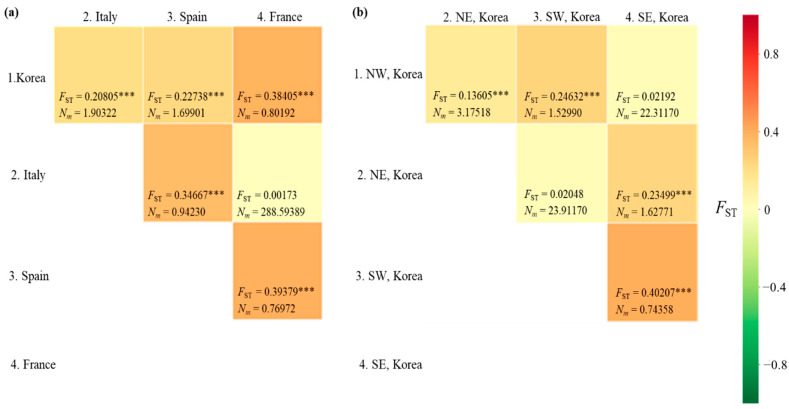
Matrix of pairwise *F*_ST_ and migration rate (*N_m_*) between pairs of countries/Korean regions. (**a**) Between countries. (**b**) Between Korean regions. NW, North−West region; NE, North−East region; SW, South−West region; SE, South−East region. *** *p* < 0.001.

The *F*_ST_ between pairs of countries ranged from 0.00173 (between Italy and France) to 0.39379 (between Spain and France), with a range of *N_m_* from 288.6 (between Italy and France) to 0.770 (between Spain and France) (Figure 4a). A significant genetic differentiation (*p* < 0.001) was observed in all country pairs, except for the comparisons between Italy and France. When the Korean localities were divided into the four regions, as we did for the diversity analysis, a significant *F*_ST_ (*p* < 0.001) was detected in four comparisons, but not in the comparisons between the North-East and South-West regions and between the North-West and South-East regions (Figure 4b). Between the North-East and South-West regions, the *N_m_* equaled 23.912. Similarly, in the comparison between the North-West and South-East regions, the *N_m_* equaled to 22.311, indicating a substantial gene flow between pairs across regions. These results indicate a higher gene flow between the regions located diagonally (between North-East and South-West regions and between North-West and South-East regions) than between those located adjacent to each other (between North and South regions).

#### 3.4.5. Structure

**Figure 5:**
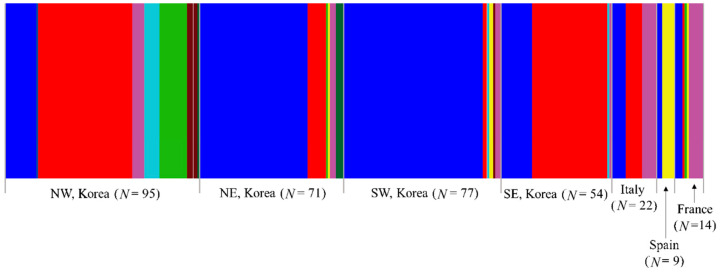
Clustering using Bayesian Analysis of Population Structure (BAPS) of the 342 individuals of *Metcalfa pruinosa* based on country/Korean region using concatenated sequences of the DNA barcoding region (658 bp) and Region 5. The optimal number of clusters (*K*) was eight.

BAPS analysis under optimal *K* = 8 showed that all eight haplogroups were found in Korea, three in Italy, two in Spain, and five in France (Figure 5). In the European countries, one haplogroup (blue), which is one of the two dominant haplogroups in Korea, was found everywhere. Another dominant haplogroup in Korea (red) was found in Italy and France, but not in Spain. The three haplogroups found in Italy were all present in France, but France also had additional haplogroups (green and yellow). These results collectively indicate that all the countries share a certain level of genetic groups, and a certain level of difference exists between the countries (Figure 5). Within Korea, seven haplogroups were found in the North-West region, six in both the North-East and South-West regions, and four in the South-East region, presenting the highest number of haplogroups in the North-West region. Among the regions, the North-West and South-East regions shared one (red) haplogroup to a great extent with each other, whereas the North-East and South-West regions shared another haplogroup extensively (blue), indicating a certain level of similarity between the North-West and South-East regions and between the North-East and South-West regions.

**Figure 6:**
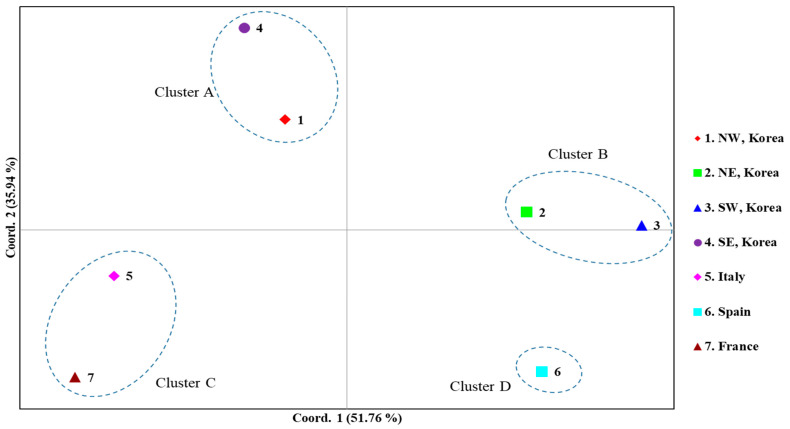
Results of principal coordinates analysis (PCoA) based on country/Korean region using the concatenated sequences of the DNA barcoding region (658 bp) and Region 5. The percentages of variation explained by the first and second axes are indicated.

A PCoA among the countries, dividing the Korean localities into four regional groups, was performed to further examine population relationships (Figure 6). The first and second components accounted for 51.76% and 35.94% of the variation, respectively. Overall, four clusters were detected: Cluster A, comprising the Korean North-West and South-East regions; Cluster B, comprising the Korean North-East and South-West regions; Cluster C, comprising Italy and France; and Cluster D, comprising solely Spain. These results are largely consistent with the *F*_ST_ estimates (Figure 4) and BAPS analysis (Figure 5) and suggest a certain level of similarity between the North-West and South-East regions and between the North-East and South-West regions in Korea.

## 4. Discussion

### 4.1. Origin and Point of Entry to Korea

To obtain further detailed inference on the origin of the Korean populations compared to previous studies [16,17], we extended our sample size for the DNA barcoding region (658 bp) to 536 individuals and the collection locality to 23 in Korea, along with eight localities in three European countries. Furthermore, the sequence data thus obtained were combined with GenBank-registered worldwide *COI* sequences (470 bp) from 294 individuals, totaling 830 individuals. However, we did not obtain any haplotypes from Korea in addition to those reported by Park et al. [17], thereby limiting the improved inference on the origin of Korean populations. Nevertheless, we were able to obtain MPH03 from a single individual collected from a North-West locality (locality 4, Yeoju; Figure S1). This haplotype had previously been detected by Park et al. [17] as three and one individual from a North-West locality (locality 4, Yeoju) and the USA, respectively, but they did not emphasize its importance, although it is shared only between the USA and Korea, and not with any European country (Table 1). Considering that an identical haplotype found commonly in the original distributional range and the introduced region could be one of the main lines of evidence that is considered as the source of introduction [32], MPH03 is a strong indicator that the USA is one of the likely sources for Korean populations.

Along with the detection of MPH03 in this location, the presence of MPH03 in that particular location in Korea implicates the point of entry to Korea. A South-East locality, Gimhae (locality 29; Figure S1), is reportedly the first location where *M. pruinosa* was detected in Korea [5]. This led to the assumption that this or the neighboring locations are likely the sole points of entry to Korea and no subsequent attempt to trace the possibility of other locations as points of entry was made. However, the presence of MPH03 in only a North-West locality suggests that the *M. pruinosa* accompanying MPH03, most likely from the USA, has been introduced through the North-West locality to Korea independently from Gimhae. Indeed, it is highly unlikely that a derived population can show evidence of a haplotype that is not found at the original point of entry, particularly considering the diffusional nature of the dispersal pattern of mitochondrial haplotypes [33]. Furthermore, our concatenated sequences showed that the diversity estimates, such as *H* and π (Table 2), and the haplogroups in BAPS (Figure 5), were the highest in the North-West region, further supporting this region as the point of entry, rather than solely a derived region.

After the first observation of *M. pruinosa* on persimmon trees in Gimhae in 2005 [5], additional nationwide monitoring was implemented [5,8,14]. In a survey in 2009–2010, *M. pruinosa* nymphs and adults were identified abundantly in Seoul and the Gyeonggi Province, which are located in the North-West region of Korea [14]. In a subsequent survey in 2011 and 2013, a more expanded distribution of *M. pruinosa* was detected in the North-West region, whereas the South-East region showed limited expansion after an earlier observation [8]. Furthermore, an absence of *M. pruinosa* was obvious in several places that are located between the North-West and South-East regions, showing a discontinuous distribution between the two regions [8,14]. These field observations, along with the finding of MPH03 only in a North-West locality, suggest that the introduction of *M. pruinosa* in Korea occurred independently to the North-West and South-East regions, and North-West introduction involved the *M. pruinosa* that originated from the USA.

The Busan Port in Korea (https://busanpa.com), which is located in the South-East region close to Gimhae, is the largest trading port in the country and handles an enormous quantity of agricultural products transported from diverse countries, including Europe and the USA. In addition, there is an international airport in Gimhae, but airplanes arriving there are only from a few Asian countries (https://airport.co.kr). In the North-West region, the Incheon (https://icpa.or.kr) and Pyeongtaek-Dangjin ports (https://gppc.or.kr) are the fourth and fifth largest ports, respectively. Similar to the Busan port, these ports handle similar quantities of agricultural produce and hardwood and cargo ships from several countries including the USA and Europe. In addition, the largest international airport is located in the northwest region of Korea. Moreover, nationwide monitoring results [5,8,14] and diversity estimates (Table 2) suggest that entry from the North-West was even more severe than that from the South-East.

Although a previous study indicated Spain as a possible source of introduction to Korea [16], the *F*_ST_ analysis with Spain in the current study was inconclusive because of the detection of only a single haplotype (MPTH01; Figure 1). Furthermore, the *F*_ST_ estimates obtained from the concatenated sequences showed a significant genetic distance between Korea and all the European countries, including Spain (Figure 4). Indeed, earlier surveys on countries exporting seedlings to Korea through the Busan port, which is close to Gimhae, did not list Spain, whereas North America, Italy, and France were listed [16]. These results collectively provide no direct evidence that would confidently identify the European countries as more potential origins of entry to Korea. Instead, we speculate that *M. pruinosa* originally detected at Gimhae may have originated from the USA rather than from European countries. However, we currently do not have direct evidence of origin from the USA into this region.

In contrast to the *F*_ST_ data, population structure analyses, such as BAPS and PCoA, both using the worldwide *COI* data (Figure S2 and Figure 2), and the concatenated sequences (Figure 5 and Figure 6) consistently indicate a certain level of genetic relationships of the Korean populations to European countries, implying European countries as the possible source of introduction to Korea. Although not conclusive, the similarity in population genetic characteristics between Korea and the European countries may be explained by the founder effect followed by population expansion, as predicted by the classical model [34,35,36]. The ancestral USA populations have diverse haplotypes; however, only one or two haplotypes (MPTH01 and MPTH02) constitute almost all the haplotypes introduced to the European countries (Table 1). Similarly, these two haplotypes are also the main ones found in Korea. Consequently, population genetic similarities among the introduced countries, such as Korea and the European countries, may be a consequence of the same origin and inevitable unless enough time has elapsed to accumulate independent genetic divergence. Such similarities may be particularly noticeable because of the low sequence divergence in the *COI* haplotypes that have been introduced to Korea and Europe (one bp difference between MPTH01 and MPTH02).

### 4.2. Expansion Characteristics in Korea

Previously, two patterns of dispersal responsible for the domestic spread of *M. pruinosa* have been proposed [5,37]. One is the short distance, active flight to the surrounding area, which facilitates the spread of the species by uninterrupted, dense belts of trees and shrubs, given the polyphagous nature of the species. The other is co-opting means of transport, such as road traffic, which is highly effective for rather longer dispersal [38,39,40,41].

Although the worldwide *COI* data did not allow the detailed investigation of the expansion dynamics of the Korean populations, the concatenation of the newly developed Region 5 to the DNA barcoding region was useful to infer expansion dynamics in Korea, and this approach allowed further thorough tracing of domestic expansion in Korea. The *F*_ST_ estimates between pairs of regions (North-East, South-East, North-West, and South-West regions) using the concatenated sequences revealed an interesting pattern. It showed a higher *N_m_* only between the North-West and South-East regions and between the North-East and South-West regions, providing non-significant *F*_ST_, even at a *p* < 0.05 level (Figure 4). Considering the geographical perspective and points of entry, which are located diagonally at opposite areas in Korea, it is extremely challenging to determine how geographically distant regions could have a higher genetic affinity than closer regions. For example, the North-West region, which includes one potential entry point to Korea, can be hypothesized to have a genetic affinity to localities in neighboring regions, such as the North-East or South-West, rather than the South-East region; however, the *F*_ST_ results did not support this hypothesis. Furthermore, the frequency of the major haplogroups was similar only between the North-East and South-West regions (blue) and between the North-West and South-East regions (red) (Figure 5). PCoA analysis also supported this clustering pattern (Figure 6).

**Figure 7:**
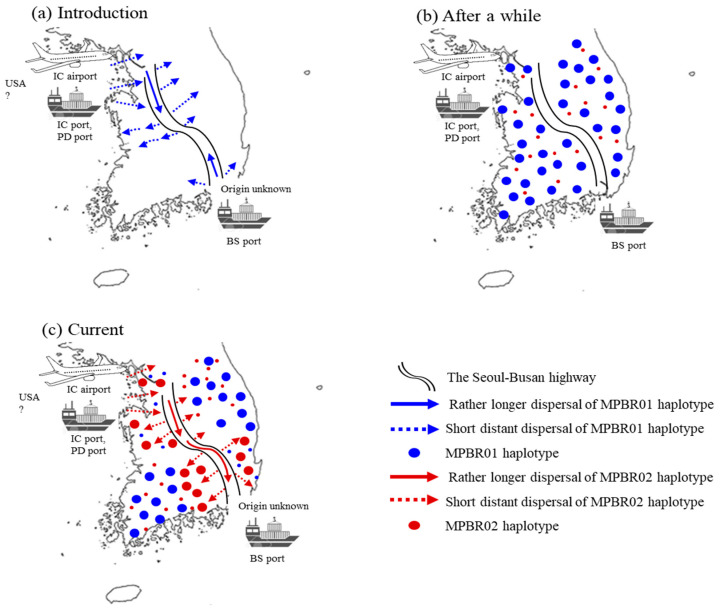
Scenarios of potential points of entry and expansion routes of *Metcalfa pruinosa* in Korea. (**a**) The initial introduction of *M. pruinosa* possessing one dominant haplotype (MPBR01) through port(s) and/or the airport located in the North-West region and Busan port located in the South-East region, and subsequent expansion primarily through the Seoul−Busan highway. (**b**) Eventual widespread distribution of MPBR01 occurs over all of Korea. (**c**) Introduction of *M. pruinosa* possessing another dominant haplotype (MPBR02) through port(s) and/or the airport located in the North-West region and widespread primarily in the North-West and South−East regions, but not yet to other locations. Incheon (IC), Pyeongtaek−Dangjin (PD), and Busan (BS).

The current *F*_ST_ data may be explained by two major independent introductions in the North-West region and subsequent dispersal through highway road traffic (Figure 7). Considering that nationwide surveys in 2011 and 2013 showed more extensive distribution of *M. pruinosa* in the North-West than that in the South-East region [5,8,14], introduction through the North-West region appears to be much more severe than through the South-East region. Alternatively, this observation could be explained by multiple introductions through the North-West region. However, a clear explanation between the two scenarios is inconclusive from the field data alone. The haplotype frequency data from the concatenated sequences were helpful and support the latter explanation. Two dominant haplotypes, MPBR01 and MPBR02, account for 52.9% and 31.3%, respectively (Table S10). The frequency of these two haplotypes in each region was concordant with the *F*_ST_ results, which showed a higher *N_m_* only between the South-East and North-West regions and between the North-East and South-West regions (Figure 3 and Figure 4). The detection of these two dominant haplotypes may indicate that each occasion of entry involved a different dominant haplotype. In one case, the introduction of *M. pruinosa* with a higher frequency of MPBR01 may have occurred; whereas another entry occasion involved *M. pruinosa* possessing MPBR02 with a higher frequency. Considering that *M. pruinosa* carrying MPBR01 has a higher frequency than MPBR02 and is distributed in a greater number of localities (Figure 3; Table S11), the earlier introduced MPBR01 became dominant throughout Korea and spread across all regions. This spread possibly occurred by two patterns of dispersal, i.e., short distance dispersal by active flight to the surrounding area and longer dispersal facilitated by road traffic [37]. *M. pruinosa* clinging on running vehicles is often observed, particularly at parking lots in highway rest areas with rich vegetation (personal observation). In an additional major introduction to the North-West region at a later time point, *M. pruinosa* bearing a higher frequency of MPBR02, which is the dominant haplotype currently in both the North-West (49.47%) and South-East (66.67%) regions, may have spread by these two patterns of dispersal. The Seoul–Busan highway that directly connects the North-West and South-East regions, may have played a major role in ensuring longer dispersal (Figure 7). This 416 km-long highway has the heaviest traffic (http://www.ex.co.kr/) as it connects Seoul, which is the capital of Korea and located in the North-West region, to Busan, which is the second largest city and located in the South-East region. The highway roughly bisects Korea diagonally into West and East regions (Figure 7). Because of this highway traffic, the rapid spread of MPBR02 may have been facilitated primarily between the North-West and South-East regions, providing a closer population genetic relationship. However, it has not yet substantially spread to the other regions. If this interpretation is accurate, MPBR02 might further expand to currently less occupied regions, such as the North-East and South-West regions, causing additional temporal changes in the haplotype frequency in Korea (Figure 7). Temporal variation in haplotype frequency in the given regions has also been reported in diverse organisms, including invasive species [42,43,44]. In contrast, the role of *M. pruinosa* entering through the South-East region is not obvious in the current population genetic structure, along with the origin. It is likely that earlier entry of *M. pruinosa* through the South-East region played a certain role in its spread to the neighboring localities, which were previously unoccupied, but our data do not show a definite role of *M. pruinosa* entering through this region.

## 5. Conclusions

The worldwide *COI* data suggests that the invasion of Korea by *M. pruinosa* occurred in two independent regions, the South-East and North-West, both of which harbor major ports and airports that import diverse agricultural produce. Introduction into the North-West region demonstrates that *M. pruinosa* likely originated from the USA because an identical haplotype was detected in Korea and the USA. However, the origin of entry to the South-East invasion is inconclusive, and further studies on populations from additional regions, including North America, are required. The concatenated sequences of the DNA barcoding region and Region 5 allowed inference of the expansion pattern within Korea, indicating that the current population genetic structure can be explained by two independent introductions into the North-West region. These introductions involved varying frequencies of the two dominant haplotypes at each time, longer dispersal facilitated via the Seoul–Busan highway, and resultant genetic connections only between diagonally located regions, such as between the North-West and South-East regions. As further samples from the introduced and native populations become available, improved inference regarding the origin and expansion pattern of *M. pruinosa* will be possible. This will be crucial for the prevention of additional invasions and quarantining of similar invasive species. This is essential considering the global biological invasion, despite the time that has elapsed since the first record of *M. pruinosa* in Korea.

## Figures and Tables

**Table 1:** Relative frequencies of worldwide mitochondrial *COI* haplotypes (470 bp) for 830 individuals of *Metcalfa pruinosa* obtained from the current study (536 individuals) and GenBank data (294 individuals).

Haplotype	Locality							
	1. Korea (659)	2. USA (36)	3. Canada (64)	4. Italy (32)	5. Spain (14)	6. France (21)	7. Slovenia (4)	Total
1. MPH01	0.943 (622)	0.028 (1)		0.781 (25)	1.000 (14)	0.476 (10)	0.500 (2)	0.812 (674)
2. MPH02	0.050 (33)	0.111 (4)		0.219 (7)		0.381 (8)	0.500 (2)	0.065 (54)
3. MPH03	0.006 (4)	0.028 (1)						0.006 (5)
4 MPH04		0.028 (1)						0.001 (1)
5. MPH05		0.056 (2)						0.002 (2)
6. MPH06		0.028 (1)						0.001 (1)
7. MPH07		0.056 (2)						0.002 (2)
8. MPH08		0.111 (4)						0.005 (4)
9. MPH09		0.028 (1)						0.001 (1)
10. MPH10		0.111 (4)	0.938 (60)					0.077 (64)
11. MPH11		0.028 (1)						0.001 (1)
12. MPH12						0.143 (3)		0.004 (3)
13. MPH13		0.028 (1)						0.001 (1)
14. MPH14		0.056 (2)						0.002 (2)
15. MPH15		0.139 (5)						0.006 (5)
16. MPH16		0.083 (3)						0.004 (3)
17. MPH17		0.083 (3)						0.004 (3)
18. MPH18			0.016 (1)					0.001 (1)
19. MPH19			0.031 (2)					0.002 (2)
20. MPH20			0.016 (1)					0.001 (1)
Total	0.794 (659)	0.043 (36)	0.077 (64)	0.039 (32)	0.019 (14)	0.025 (21)	0.005 (4)	

Numbers in parentheses indicate the sample size for each population.

**Table 2:** Within-country and Korean regional diversity estimates of *Metcalfa pruinosa* based on concatenated sequences of the DNA barcoding region and Region 5.

Locality	SS ^a^	NH ^b^	*H* ^c^	NP ^d^	MSD ^e^ (%)	MPD ^f^	π ^g^
1. Korea	297	17	0.6414 ± 0.0209	13	0.92	1.958367	0.001803 ± 0.001133
NW, Korea	95	12	0.7118 ± 0.0416	11	0.92	2.407615	0.002217 ± 0.001346
NE, Korea	71	7	0.4306 ± 0.0700	11	0.92	1.814085	0.001670 ± 0.001080
SW, Korea	77	7	0.2201 ± 0.0629	11	0.92	1.013671	0.000933 ± 0.000702
SE, Korea	54	5	0.4864 ± 0.0584	10	0.83	1.217331	0.001121 ± 0.000804
2. Italy	22	3	0.6970 ± 0.0324	9	0.83	4.090909	0.003767 ± 0.002177
3. Spain	9	3	0.6389 ± 0.1258	3	0.28	1.000000	0.000924 ± 0.000775
4. France	14	6	0.7692 ± 0.0895	13	0.92	5.395604	0.004968 ± 0.002860

^a^ Sample size; ^b^ Number of haplotypes; ^c^ Haplotype diversity with standard error; ^d^ Number of polymorphic sites; ^e^ Maximum sequence divergence; ^f^ Mean number of pairwise differences; ^g^ Nucleotide diversity with standard error; NW, North-West region; NE, North-East region; SW, South-West region; and SE, South East region. Figure 3 shows regional information.

## Supplementary Materials

The following are available online at https://www.mdpi.com/2075-4450/12/1/4/s1, Figure S1: Sampling locations of *Metcalfa pruinosa*. General locality names are as follows: Korea (1, Seoul; 2, Incheon; 3, Anseong; 4, Yeoju; 5, Paju; 6, Incheon; 7, Hwaseong; 8, Gwacheon; 9, Yongun; 10, Suwon; 11, Pyeongchang; 12, Wonju; 13, Danyang; 14, Goesan; 15, Eumsung; 16, Jincheon; 17, Chungju; 18, Cheongwon; 19, Gongju; 20, Geumsan; 21, Taean; 22, Yesan; 23, Cheonan; 24, Daegu; 25, Cheongdo; 26, Chilgok; 27, Sangju; 28, Yeongju; 29, Gimhae; 30, Miryang; 31, Iksan; 32, Gokseong; 33, Haenam; 34, Jangseong; and 35, Suncheon), USA (36, Maryland; 37, New Jersey; and 38, West Virginia), Canada (39, Ontario; and 40, Saskatchewan), Italy (41, San Remo; 42, Savona; 43, Borghetto; and 44, Genova), Spain (45, Lleida), France [46, INRA (The French National Institute for Agricultural Research); 47, Montpellier; 48, ARS (Ars-sur-Formans); and 49, Le Boulou], and Slovenia (50, Pri Hrastu), Figure S2: Clustering with Bayesian Analysis of Population Structure (BAPS) of *Metcalfa pruinosa* based on country using the worldwide *COI* data. The optimal number of clusters (*K*) was seven, Table S1: List of *Metcalfa pruinosa* samples sequenced for a DNA barcoding region (658 bp) and Region 5 (428 bp), Table S2: List of primers used to amplify the variable regions detected during the comparison between two mitochondrial genomes of *Metcalfa pruinose*, Table S3: List of worldwide *COI* sequences of *Metcalfa pruinosa* (830 individuals), including those obtained in this study, Table S4: Relative frequencies of the DNA barcoding region (658 bp)-based haplotypes for 536 individuals of *Metcalfa pruinosa* collected in Korea and the three European countries, Table S5: Within-country diversity estimates of *Metcalfa pruinosa* based on worldwide *COI* sequences, Table S6: Pairwise comparisons among 15 Region 5 haplotypes of *Metcalfa pruinose*, Table S7: Relative frequencies of Region 5 haplotypes for 342 individuals of *Metcalfa pruinosa* collected in Korea and the three European countries, Table S8: Comparison of the three types of marker regions, Table S9: Variable positions in the concatenated sequences of the DNA barcoding region and Region 5 in *Metcalfa pruinosa,* Table S10: Country-based relative frequencies of the concatenated sequence-based haplotypes (658 bp of the DNA barcoding region and Region 5) for 342 individuals of *Metcalfa pruinosa* collected in Korea and the three European countries, Table S11: Locality-based relative frequencies of the concatenated sequence-based haplotypes (658 bp of the DNA barcoding region and Region 5) for 342 individuals of *Metcalfa pruinosa* collected in Korea and the European countries.
