# Supplementary material for: Tracing the Invasion and Expansion Characteristics of the Flatid Planthopper, Metcalfa pruinosa (Hemiptera: Flatidae), in Korea Using Mitochondrial DNA Sequences
Source: Insects. 2020 Dec 23;12(1):4. doi: 10.3390/insects12010004 (PMC7822484; doi:10.3390/insects12010004)
Supplement: Supplementary file 1 [file insects-12-00004-s001.zip › supplementary-xml/All Sup. Tables 1-11.pdf]

**Table S1.** List of *Metcalfa pruinosa* samples sequenced for a DNA barcoding region (658 bp) and Region 5 (428 bp)

| Locality (collection date/ sample size) | GenBank animal number | Sample voucher | COI haplotype* | GenBank no. | Region 5 haplotype | GenBank no. | COI + Region 5 haplotype |
|---|---|---|---|---|---|---|---|
| 1. Seoul, Korea (Oct. 09, 2013/ 24) | SSW01 | 7978 | MPBAR01 | MK303248 | - | - | - |
| | SSW02 | 7979 | MPBAR01 | MK303249 | - | - | - |
| | SSW03 | 7980 | MPBAR01 | MK303250 | - | - | - |
| | SSW04 | 7981 | MPBAR01 | MK303251 | - | - | - |
| | SSW05 | 7982 | MPBAR01 | MK303252 | - | - | - |
| | SSW06 | 7983 | MPBAR01 | MK303253 | - | - | - |
| | SSW07 | 7984 | MPBAR01 | MK303254 | - | - | - |
| | SSW08 | 7985 | MPBAR01 | MK303255 | - | - | - |
| | SSW10 | 7986 | MPBAR01 | MK303256 | - | - | - |
| | SSW12 | 7987 | MPBAR01 | MK303257 | - | - | - |
| | SSW14 | 7988 | MPBAR01 | MK303258 | - | - | - |
| | SSW15 | 7989 | MPBAR01 | MK303259 | - | - | - |
| | SSW16 | 7990 | MPBAR01 | MK303260 | - | - | - |
| | SSW17 | 7991 | MPBAR02 | MK303261 | - | - | - |
| | SSW18 | 7992 | MPBAR01 | MK303262 | - | - | - |
| | SSW19 | 7993 | MPBAR01 | MK303263 | - | - | - |
| | SSW20 | 7994 | MPBAR02 | MK303264 | - | - | - |
| | SSW23 | 7995 | MPBAR01 | MK303265 | - | - | - |
| | SSW25 | 7996 | MPBAR01 | MK303266 | - | - | - |
| | SSW27 | 7997 | MPBAR01 | MK303267 | - | - | - |
| | SSW28 | 7998 | MPBAR01 | MK303268 | - | - | - |
| | SSW29 | 7999 | MPBAR01 | MK303269 | - | - | - |
| | SSW30 | 8000 | MPBAR01 | MK303270 | - | - | - |
| | SSW31 | 8001 | MPBAR01 | MK303271 | - | - | - |
| 2. Incheon, GG, Korea (Jun. 20, 2016/ 20) | IC06 | 7853 | MPBAR01 | MK303123 | MPR502 | MN790241 | MPBR02 |
| | IC07 | 7854 | MPBAR01 | MK303124 | MPR502 | MN790242 | MPBR02 |
| | IC08 | 7855 | MPBAR01 | MK303125 | MPR502 | MN790243 | MPBR02 |
| | IC09 | 7856 | MPBAR01 | MK303126 | MPR502 | MN790244 | MPBR02 |
| | IC10 | 7857 | MPBAR01 | MK303127 | - | - | - |
| | IC11 | 7858 | MPBAR01 | MK303128 | MPR502 | MN790245 | MPBR02 |
| | IC12 | 7859 | MPBAR01 | MK303129 | MPR502 | MN790246 | MPBR02 |
| | IC13 | 7860 | MPBAR01 | MK303130 | MPR502 | MN790247 | MPBR02 |
| | IC14 | 7861 | MPBAR01 | MK303131 | MPR502 | MN790248 | MPBR02 |
| | IC15 | 7862 | MPBAR01 | MK303132 | MPR502 | MN790249 | MPBR02 |
| | IC16 | 7863 | MPBAR01 | MK303133 | MPR502 | MN790250 | MPBR02 |
| | IC17 | 7864 | MPBAR01 | MK303134 | MPR502 | MN790251 | MPBR02 |
| | IC18 | 7865 | MPBAR01 | MK303135 | MPR502 | MN790252 | MPBR02 |
| | IC21 | 7866 | MPBAR01 | MK303136 | MPR502 | MN790253 | MPBR02 |
| | IC22 | 7867 | MPBAR01 | MK303137 | MPR502 | MN790254 | MPBR02 |
| | IC24 | 7868 | MPBAR01 | MK303138 | MPR502 | MN790255 | MPBR02 |
| | IC26 | 7869 | MPBAR01 | MK303139 | - | - | - |
| | IC27 | 7870 | MPBAR01 | MK303140 | - | - | - |
| | IC28 | 7871 | MPBAR01 | MK303141 | - | - | - |
| | IC29 | 7872 | MPBAR01 | MK303142 | - | - | - |
| 3. Anseong, GG, Korea (Jun. 26, 2013/ 21) | GAS01 | 7707 | MPBAR01 | MK302977 | - | - | - |
| | GAS05 | 7708 | MPBAR01 | MK302978 | - | - | - |
| | GAS06 | 7709 | MPBAR01 | MK302979 | - | - | - |
| | GAS08 | 7710 | MPBAR01 | MK302980 | - | - | - |
| | GAS09 | 7711 | MPBAR01 | MK302981 | - | - | - |
| | GAS10 | 7712 | MPBAR02 | MK302982 | - | - | - |
| | GAS11 | 7713 | MPBAR01 | MK302983 | - | - | - |
| | GAS12 | 7714 | MPBAR01 | MK302984 | - | - | - |
| | GAS13 | 7715 | MPBAR02 | MK302985 | - | - | - |
| | GAS14 | 7716 | MPBAR01 | MK302986 | - | - | - |
| | GAS15 | 7717 | MPBAR01 | MK302987 | - | - | - |
| | GAS17 | 7718 | MPBAR01 | MK302988 | - | - | - |

| | | | | | | | |
|---|---|---|---|---|---|---|---|
| | GAS18 | 7719 | MPBAR01 | MK302989 | - | - | - |
| | GAS19 | 7720 | MPBAR01 | MK302990 | - | - | - |
| | GAS20 | 7721 | MPBAR01 | MK302991 | - | - | - |
| | GAS24 | 7722 | MPBAR01 | MK302992 | - | - | - |
| | GAS25 | 7723 | MPBAR01 | MK302993 | - | - | - |
| | GAS26 | 7724 | MPBAR01 | MK302994 | - | - | - |
| | GAS28 | 7725 | MPBAR01 | MK302995 | - | - | - |
| | GAS29 | 7726 | MPBAR01 | MK302996 | - | - | - |
| | GAS30 | 7727 | MPBAR01 | MK302997 | - | - | - |
| 4. Yeoju, GG, Korea (Jun. 25, 2013/ 22) | GYJ02 | 10605 | MPBAR01 | MN790203 | MPR501 | MN790256 | MPBR01 |
| | GYJ04 | 10606 | MPBAR01 | MN790204 | MPR501 | MN790257 | MPBR01 |
| | GYJ06 | 7833 | MPBAR01 | MK303103 | MPR501 | MN790258 | MPBR01 |
| | GYJ07 | 7834 | MPBAR02 | MK303104 | - | - | - |
| | GYJ08 | 7835 | MPBAR01 | MK303105 | MPR501 | MN790259 | MPBR01 |
| | GYJ10 | 7836 | MPBAR01 | MK303106 | MPR503 | MN790260 | MPBR03 |
| | GYJ11 | 7837 | MPBAR01 | MK303107 | MPR506 | MN790261 | MPBR06 |
| | GYJ12 | 7838 | MPBAR01 | MK303108 | MPR501 | MN790262 | MPBR01 |
| | GYJ13 | 7839 | MPBAR02 | MK303109 | MPR501 | MN790263 | MPBR14 |
| | GYJ14 | 7840 | MPBAR02 | MK303110 | MPR510 | MN790264 | MPBR18 |
| | GYJ15 | 7841 | MPBAR02 | MK303111 | MPR501 | MN790265 | MPBR14 |
| | GYJ16 | 7842 | MPBAR01 | MK303112 | MPR503 | MN790266 | MPBR03 |
| | GYJ17 | 7843 | MPBAR01 | MK303113 | - | - | - |
| | GYJ18 | 7844 | MPBAR03 | MK303114 | MPR510 | MN790267 | MPBR20 |
| | GYJ19 | 7845 | MPBAR02 | MK303115 | MPR502 | MN790268 | MPBR15 |
| | GYJ20 | 7846 | MPBAR01 | MK303116 | MPR501 | MN790269 | MPBR01 |
| | GYJ21 | 7847 | MPBAR02 | MK303117 | MPR502 | MN790270 | MPBR15 |
| | GYJ23 | 7848 | MPBAR01 | MK303118 | MPR503 | MN790271 | MPBR03 |
| | GYJ24 | 7849 | MPBAR02 | MK303119 | MPR503 | MN790272 | MPBR16 |
| | GYJ25 | 7850 | MPBAR02 | MK303120 | MPR502 | MN790273 | MPBR15 |
| | GYJ26 | 7851 | MPBAR01 | MK303121 | - | - | - |
| | GYJ29 | 7852 | MPBAR01 | MK303122 | - | - | - |
| 5. Pyeongchang, GW, Korea (Jul. 30, 2018/ 20) | GWPC01 | 7793 | MPBAR01 | MK303063 | MPR501 | MN790274 | MPBR01 |
| | GWPC02 | 7794 | MPBAR01 | MK303064 | MPR501 | MN790275 | MPBR01 |
| | GWPC03 | 7795 | MPBAR01 | MK303065 | MPR501 | MN790276 | MPBR01 |
| | GWPC04 | 7796 | MPBAR01 | MK303066 | MPR501 | MN790277 | MPBR01 |
| | GWPC05 | 7797 | MPBAR02 | MK303067 | MPR506 | MN790278 | MPBR17 |
| | GWPC06 | 7798 | MPBAR02 | MK303068 | MPR506 | MN790279 | MPBR17 |
| | GWPC07 | 7799 | MPBAR01 | MK303069 | MPR502 | MN790280 | MPBR02 |
| | GWPC08 | 7800 | MPBAR01 | MK303070 | MPR501 | MN790281 | MPBR01 |
| | GWPC09 | 7801 | MPBAR01 | MK303071 | MPR501 | MN790282 | MPBR01 |
| | GWPC10 | 7802 | MPBAR02 | MK303072 | MPR506 | MN790283 | MPBR17 |
| | GWPC11 | 7803 | MPBAR01 | MK303073 | MPR501 | MN790284 | MPBR01 |
| | GWPC12 | 7804 | MPBAR02 | MK303074 | MPR506 | MN790285 | MPBR17 |
| | GWPC13 | 7805 | MPBAR01 | MK303075 | - | - | - |
| | GWPC14 | 7806 | MPBAR01 | MK303076 | MPR501 | MN790286 | MPBR01 |
| | GWPC15 | 7807 | MPBAR01 | MK303077 | MPR501 | MN790287 | MPBR01 |
| | GWPC16 | 7808 | MPBAR01 | MK303078 | MPR508 | MN790288 | MPBR08 |
| | GWPC17 | 7809 | MPBAR01 | MK303079 | MPR501 | MN790289 | MPBR01 |
| | GWPC18 | 7810 | MPBAR01 | MK303080 | - | - | - |
| | GWPC19 | 7811 | MPBAR01 | MK303081 | MPR501 | MN790290 | MPBR01 |
| | GWPC20 | 7812 | MPBAR01 | MK303082 | MPR501 | MN790291 | MPBR01 |
| 6. Wonju, GW, Korea (Jul. 19, 2018/ 20) | GWWJ01 | 7813 | MPBAR01 | MK303083 | MPR502 | MN790292 | MPBR02 |
| | GWWJ02 | 7814 | MPBAR01 | MK303084 | MPR501 | MN790293 | MPBR01 |
| | GWWJ03 | 7815 | MPBAR01 | MK303085 | MPR511 | MN790294 | MPBR10 |
| | GWWJ04 | 7816 | MPBAR01 | MK303086 | MPR501 | MN790295 | MPBR01 |
| | GWWJ05 | 7817 | MPBAR01 | MK303087 | MPR502 | MN790296 | MPBR02 |
| | GWWJ06 | 7818 | MPBAR01 | MK303088 | MPR502 | MN790297 | MPBR02 |
| | GWWJ07 | 7819 | MPBAR01 | MK303089 | MPR502 | MN790298 | MPBR02 |
| | GWWJ08 | 7820 | MPBAR01 | MK303090 | MPR502 | MN790299 | MPBR02 |
| | GWWJ09 | 7821 | MPBAR01 | MK303091 | MPR502 | MN790300 | MPBR02 |
| | GWWJ10 | 7822 | MPBAR01 | MK303092 | MPR501 | MN790301 | MPBR01 |

| | | | | | | | |
|---|---|---|---|---|---|---|---|
| | GWWJ11 | 7823 | MPBAR01 | MK303093 | MPR501 | MN790302 | MPBR01 |
| | GWWJ12 | 7824 | MPBAR01 | MK303094 | - | - | - |
| | GWWJ13 | 7825 | MPBAR01 | MK303095 | MPR501 | MN790303 | MPBR01 |
| | GWWJ14 | 7826 | MPBAR01 | MK303096 | MPR501 | MN790304 | MPBR01 |
| | GWWJ15 | 7827 | MPBAR01 | MK303097 | MPR501 | MN790305 | MPBR01 |
| | GWWJ16 | 7828 | MPBAR01 | MK303098 | MPR501 | MN790306 | MPBR01 |
| | GWWJ17 | 7829 | MPBAR01 | MK303099 | MPR501 | MN790307 | MPBR01 |
| | GWWJ18 | 7830 | MPBAR01 | MK303100 | MPR501 | MN790308 | MPBR01 |
| | GWWJ19 | 7831 | MPBAR01 | MK303101 | MPR501 | MN790309 | MPBR01 |
| | GWWJ20 | 7832 | MPBAR01 | MK303102 | MPR501 | MN790310 | MPBR01 |
| 7. Danyang, CB, Korea (Jun. 25, 2016/ 23) | CBDY01 | 7549 | MPBAR01 | MK302819 | MPR501 | MN790311 | MPBR01 |
| | CBDY02 | 7550 | MPBAR01 | MK302820 | - | - | - |
| | CBDY03 | 7551 | MPBAR01 | MK302821 | MPR501 | MN790312 | MPBR01 |
| | CBDY05 | 7552 | MPBAR01 | MK302822 | MPR501 | MN790313 | MPBR01 |
| | CBDY06 | 7553 | MPBAR01 | MK302823 | MPR501 | MN790314 | MPBR01 |
| | CBDY07 | 7554 | MPBAR02 | MK302824 | MPR503 | MN790315 | MPBR16 |
| | CBDY08 | 7555 | MPBAR01 | MK302825 | MPR501 | MN790316 | MPBR01 |
| | CBDY09 | 7556 | MPBAR01 | MK302826 | MPR501 | MN790317 | MPBR01 |
| | CBDY10 | 7557 | MPBAR01 | MK302827 | MPR501 | MN790318 | MPBR01 |
| | CBDY11 | 7558 | MPBAR01 | MK302828 | MPR501 | MN790319 | MPBR01 |
| | CBDY13 | 7559 | MPBAR01 | MK302829 | MPR501 | MN790320 | MPBR01 |
| | CBDY14 | 7560 | MPBAR01 | MK302830 | MPR501 | MN790321 | MPBR01 |
| | CBDY16 | 7561 | MPBAR01 | MK302831 | MPR501 | MN790322 | MPBR01 |
| | CBDY17 | 7562 | MPBAR01 | MK302832 | MPR501 | MN790323 | MPBR01 |
| | CBDY19 | 7563 | MPBAR01 | MK302833 | MPR501 | MN790324 | MPBR01 |
| | CBDY20 | 7564 | MPBAR01 | MK302834 | MPR501 | MN790325 | MPBR01 |
| | CBDY22 | 7565 | MPBAR01 | MK302835 | MPR501 | MN790326 | MPBR01 |
| | CBDY23 | 7566 | MPBAR01 | MK302836 | MPR501 | MN790327 | MPBR01 |
| | CBDY25 | 7567 | MPBAR01 | MK302837 | MPR501 | MN790328 | MPBR01 |
| | CBDY26 | 7568 | MPBAR01 | MK302838 | MPR501 | MN790329 | MPBR01 |
| | CBDY27 | 7569 | MPBAR01 | MK302839 | MPR501 | MN790330 | MPBR01 |
| | CBDY30 | 7570 | MPBAR01 | MK302840 | MPR501 | MN790331 | MPBR01 |
| | CBDY31 | 7571 | MPBAR01 | MK302841 | MPR501 | MN790332 | MPBR01 |
| 8. Goesan, CB, Korea (Sep. 26, 2013/ 21) | CBGS01 | 7572 | MPBAR01 | MK302842 | MPR508 | MN790333 | MPBR08 |
| | CBGS02 | 7573 | MPBAR01 | MK302843 | MPR502 | MN790334 | MPBR02 |
| | CBGS03 | 7574 | MPBAR01 | MK302844 | MPR508 | MN790335 | MPBR08 |
| | CBGS04 | 7575 | MPBAR01 | MK302845 | MPR502 | MN790336 | MPBR02 |
| | CBGS06 | 7576 | MPBAR01 | MK302846 | MPR508 | MN790337 | MPBR08 |
| | CBGS09 | 7577 | MPBAR01 | MK302847 | MPR502 | MN790338 | MPBR02 |
| | CBGS10 | 7578 | MPBAR01 | MK302848 | MPR502 | MN790339 | MPBR02 |
| | CBGS11 | 7579 | MPBAR01 | MK302849 | MPR502 | MN790340 | MPBR02 |
| | CBGS14 | 7580 | MPBAR01 | MK302850 | MPR508 | MN790341 | MPBR08 |
| | CBGS17 | 7581 | MPBAR01 | MK302851 | MPR508 | MN790342 | MPBR08 |
| | CBGS18 | 7582 | MPBAR01 | MK302852 | MPR508 | MN790343 | MPBR08 |
| | CBGS19 | 7583 | MPBAR01 | MK302853 | MPR502 | MN790344 | MPBR02 |
| | CBGS20 | 7584 | MPBAR01 | MK302854 | MPR508 | MN790345 | MPBR08 |
| | CBGS22 | 7585 | MPBAR01 | MK302855 | MPR501 | MN790346 | MPBR01 |
| | CBGS23 | 7586 | MPBAR01 | MK302856 | MPR508 | MN790347 | MPBR08 |
| | CBGS24 | 7587 | MPBAR01 | MK302857 | MPR502 | MN790348 | MPBR02 |
| | CBGS25 | 7588 | MPBAR01 | MK302858 | MPR508 | MN790349 | MPBR08 |
| | CBGS26 | 7589 | MPBAR01 | MK302859 | MPR508 | MN790350 | MPBR08 |
| | CBGS27 | 7590 | MPBAR01 | MK302860 | MPR508 | MN790351 | MPBR08 |
| | CBGS29 | 7591 | MPBAR01 | MK302861 | MPR508 | MN790352 | MPBR08 |
| | CBGS32 | 7592 | MPBAR01 | MK302862 | MPR508 | MN790353 | MPBR08 |
| 9. Gongju, CN, Korea (Sep. 01, 2013/ 22) | CNGJ01 | 7593 | MPBAR02 | MK302863 | - | - | - |
| | CNGJ02 | 7594 | MPBAR02 | MK302864 | - | - | - |
| | CNGJ03 | 7595 | MPBAR01 | MK302865 | - | - | - |
| | CNGJ04 | 7596 | MPBAR01 | MK302866 | - | - | - |
| | CNGJ05 | 7597 | MPBAR01 | MK302867 | - | - | - |
| | CNGJ06 | 7598 | MPBAR01 | MK302868 | - | - | - |
| | CNGJ09 | 7599 | MPBAR01 | MK302869 | - | - | - |

| | | | | | | | |
|---|---|---|---|---|---|---|---|
| | CNGJ10 | 7600 | MPBAR01 | MK302870 | - | - | - |
| | CNGJ11 | 7601 | MPBAR01 | MK302871 | - | - | - |
| | CNGJ12 | 7602 | MPBAR01 | MK302872 | - | - | - |
| | CNGJ13 | 7603 | MPBAR01 | MK302873 | - | - | - |
| | CNGJ14 | 7604 | MPBAR01 | MK302874 | - | - | - |
| | CNGJ15 | 7605 | MPBAR01 | MK302875 | - | - | - |
| | CNGJ16 | 7606 | MPBAR01 | MK302876 | - | - | - |
| | CNGJ17 | 7607 | MPBAR01 | MK302877 | - | - | - |
| | CNGJ18 | 7608 | MPBAR01 | MK302878 | - | - | - |
| | CNGJ19 | 7609 | MPBAR01 | MK302879 | - | - | - |
| | CNGJ20 | 7610 | MPBAR01 | MK302880 | - | - | - |
| | CNGJ21 | 7611 | MPBAR01 | MK302881 | - | - | - |
| | CNGJ22 | 7612 | MPBAR01 | MK302882 | - | - | - |
| | CNGJ23 | 7613 | MPBAR01 | MK302883 | - | - | - |
| | CNGJ24 | 7614 | MPBAR01 | MK302884 | - | - | - |
| 10. Geumsan, CN, Korea (Jul. 15, 2013/ 23) | CNGS01 | 7615 | MPBAR01 | MK302885 | - | - | - |
| | CNGS04 | 7616 | MPBAR01 | MK302886 | - | - | - |
| | CNGS07 | 7617 | MPBAR01 | MK302887 | - | - | - |
| | CNGS08 | 7618 | MPBAR01 | MK302888 | - | - | - |
| | CNGS09 | 7619 | MPBAR01 | MK302889 | - | - | - |
| | CNGS10 | 7620 | MPBAR01 | MK302890 | - | - | - |
| | CNGS11 | 7621 | MPBAR01 | MK302891 | - | - | - |
| | CNGS12 | 7622 | MPBAR01 | MK302892 | - | - | - |
| | CNGS13 | 7623 | MPBAR01 | MK302893 | - | - | - |
| | CNGS14 | 7624 | MPBAR01 | MK302894 | - | - | - |
| | CNGS15 | 7625 | MPBAR01 | MK302895 | - | - | - |
| | CNGS16 | 7626 | MPBAR01 | MK302896 | - | - | - |
| | CNGS17 | 7627 | MPBAR01 | MK302897 | - | - | - |
| | CNGS19 | 7628 | MPBAR01 | MK302898 | - | - | - |
| | CNGS20 | 7629 | MPBAR01 | MK302899 | - | - | - |
| | CNGS22 | 7630 | MPBAR01 | MK302900 | - | - | - |
| | CNGS23 | 7631 | MPBAR01 | MK302901 | - | - | - |
| | CNGS24 | 7632 | MPBAR01 | MK302902 | - | - | - |
| | CNGS25 | 7633 | MPBAR01 | MK302903 | - | - | - |
| | CNGS27 | 7634 | MPBAR01 | MK302904 | - | - | - |
| | CNGS28 | 7635 | MPBAR01 | MK302905 | - | - | - |
| | CNGS29 | 7636 | MPBAR01 | MK302906 | - | - | - |
| | CNGS30 | 7637 | MPBAR01 | MK302907 | - | - | - |
| 11. Taean, CN, Korea (Jun. 20, 2013/ 26) | CNTA01 | 7638 | MPBAR01 | MK302908 | MPR502 | MN790354 | MPBR02 |
| | CNTA02 | 7639 | MPBAR01 | MK302909 | MPR509 | MN790355 | MPBR09 |
| | CNTA03 | 7640 | MPBAR01 | MK302910 | MPR501 | MN790356 | MPBR01 |
| | CNTA04 | 7641 | MPBAR01 | MK302911 | - | - | - |
| | CNTA05 | 7642 | MPBAR01 | MK302912 | MPR504 | MN790357 | MPBR04 |
| | CNTA06 | 7643 | MPBAR01 | MK302913 | MPR504 | MN790358 | MPBR04 |
| | CNTA07 | 7644 | MPBAR01 | MK302914 | MPR504 | MN790359 | MPBR04 |
| | CNTA08 | 7645 | MPBAR01 | MK302915 | MPR502 | MN790360 | MPBR02 |
| | CNTA09 | 7646 | MPBAR01 | MK302916 | MPR502 | MN790361 | MPBR02 |
| | CNTA21 | 7647 | MPBAR01 | MK302917 | - | - | - |
| | CNTA23 | 7648 | MPBAR01 | MK302918 | MPR509 | MN790362 | MPBR09 |
| | CNTA24 | 7649 | MPBAR01 | MK302919 | MPR504 | MN790363 | MPBR04 |
| | CNTA25 | 7650 | MPBAR01 | MK302920 | MPR502 | MN790364 | MPBR02 |
| | CNTA26 | 7651 | MPBAR01 | MK302921 | - | - | - |
| | CNTA28 | 7652 | MPBAR01 | MK302922 | MPR501 | MN790365 | MPBR01 |
| | CNTA29 | 7653 | MPBAR01 | MK302923 | MPR502 | MN790366 | MPBR02 |
| | CNTA31 | 7654 | MPBAR01 | MK302924 | MPR502 | MN790367 | MPBR02 |
| | CNTA32 | 7655 | MPBAR01 | MK302925 | MPR502 | MN790368 | MPBR02 |
| | CNTA33 | 7656 | MPBAR01 | MK302926 | MPR502 | MN790369 | MPBR02 |
| | CNTA34 | 7657 | MPBAR01 | MK302927 | MPR502 | MN790370 | MPBR02 |
| | CNTA35 | 7658 | MPBAR01 | MK302928 | MPR502 | MN790371 | MPBR02 |
| | CNTA36 | 7659 | MPBAR01 | MK302929 | MPR502 | MN790372 | MPBR02 |
| | CNTA37 | 7660 | MPBAR01 | MK302930 | MPR501 | MN790373 | MPBR01 |

| | | | | | | | |
|---|---|---|---|---|---|---|---|
| | CNTA38 | 7661 | MPBAR01 | MK302931 | MPR501 | MN790374 | MPBR01 |
| | CNTA39 | 7662 | MPBAR01 | MK302932 | MPR502 | MN790375 | MPBR02 |
| | CNTA40 | 7663 | MPBAR01 | MK302933 | MPR501 | MN790376 | MPBR01 |
| 12. Yesan, CN, Korea (Sep. 14, 2018/ 23) | CNYS01 | 7664 | MPBAR01 | MK302934 | MPR501 | MN790377 | MPBR01 |
| | CNYS02 | 7665 | MPBAR01 | MK302935 | MPR501 | MN790378 | MPBR01 |
| | CNYS03 | 7666 | MPBAR01 | MK302936 | - | - | - |
| | CNYS04 | 7667 | MPBAR01 | MK302937 | MPR502 | MN790379 | MPBR02 |
| | CNYS05 | 7668 | MPBAR01 | MK302938 | MPR502 | MN790380 | MPBR02 |
| | CNYS06 | 7669 | MPBAR01 | MK302939 | - | - | - |
| | CNYS07 | 7670 | MPBAR01 | MK302940 | MPR502 | MN790381 | MPBR02 |
| | CNYS08 | 7671 | MPBAR01 | MK302941 | MPR502 | MN790382 | MPBR02 |
| | CNYS09 | 7672 | MPBAR01 | MK302942 | MPR504 | MN790383 | MPBR04 |
| | CNYS10 | 7673 | MPBAR01 | MK302943 | MPR502 | MN790384 | MPBR02 |
| | CNYS11 | 7674 | MPBAR01 | MK302944 | MPR502 | MN790385 | MPBR02 |
| | CNYS12 | 7675 | MPBAR01 | MK302945 | - | - | - |
| | CNYS13 | 7676 | MPBAR01 | MK302946 | MPR504 | MN790386 | MPBR04 |
| | CNYS14 | 7677 | MPBAR01 | MK302947 | MPR502 | MN790387 | MPBR02 |
| | CNYS15 | 7678 | MPBAR01 | MK302948 | MPR502 | MN790388 | MPBR02 |
| | CNYS16 | 7679 | MPBAR01 | MK302949 | MPR502 | MN790389 | MPBR02 |
| | CNYS17 | 7680 | MPBAR01 | MK302950 | MPR502 | MN790390 | MPBR02 |
| | CNYS18 | 7681 | MPBAR01 | MK302951 | MPR502 | MN790391 | MPBR02 |
| | CNYS19 | 7682 | MPBAR01 | MK302952 | MPR502 | MN790392 | MPBR02 |
| | CNYS20 | 7683 | MPBAR01 | MK302953 | - | - | - |
| | CNYS22 | 7684 | MPBAR01 | MK302954 | MPR501 | MN790393 | MPBR01 |
| | CNYS28 | 7685 | MPBAR01 | MK302955 | MPR502 | MN790394 | MPBR02 |
| | CNYS30 | 7686 | MPBAR01 | MK302956 | - | - | - |
| 13. Daegu, Korea (Jul. 12, 2018/ 20) | DG02 | 7687 | MPBAR01 | MK302957 | - | - | - |
| | DG03 | 7688 | MPBAR01 | MK302958 | - | - | - |
| | DG04 | 7689 | MPBAR01 | MK302959 | - | - | - |
| | DG05 | 7690 | MPBAR01 | MK302960 | - | - | - |
| | DG06 | 7691 | MPBAR01 | MK302961 | - | - | - |
| | DG07 | 7692 | MPBAR01 | MK302962 | - | - | - |
| | DG08 | 7693 | MPBAR01 | MK302963 | - | - | - |
| | DG09 | 7694 | MPBAR01 | MK302964 | - | - | - |
| | DG11 | 7695 | MPBAR01 | MK302965 | - | - | - |
| | DG12 | 7696 | MPBAR01 | MK302966 | - | - | - |
| | DG13 | 7697 | MPBAR01 | MK302967 | - | - | - |
| | DG14 | 7698 | MPBAR01 | MK302968 | - | - | - |
| | DG15 | 7699 | MPBAR01 | MK302969 | - | - | - |
| | DG16 | 7700 | MPBAR01 | MK302970 | - | - | - |
| | DG17 | 7701 | MPBAR01 | MK302971 | - | - | - |
| | DG18 | 7702 | MPBAR01 | MK302972 | - | - | - |
| | DG19 | 7703 | MPBAR01 | MK302973 | - | - | - |
| | DG20 | 7704 | MPBAR01 | MK302974 | - | - | - |
| | DG21 | 7705 | MPBAR01 | MK302975 | - | - | - |
| | DG22 | 7706 | MPBAR01 | MK302976 | - | - | - |
| 14. Cheongdo, GB, Korea (Jul. 09, 2018/ 20) | GBCD01 | 7728 | MPBAR01 | MK302998 | MPR502 | MN790395 | MPBR02 |
| | GBCD03 | 7729 | MPBAR01 | MK302999 | MPR502 | MN790396 | MPBR02 |
| | GBCD04 | 7730 | MPBAR01 | MK303000 | MPR502 | MN790397 | MPBR02 |
| | GBCD05 | 7731 | MPBAR01 | MK303001 | - | - | - |
| | GBCD06 | 7732 | MPBAR01 | MK303002 | MPR502 | MN790398 | MPBR02 |
| | GBCD07 | 7733 | MPBAR01 | MK303003 | MPR502 | MN790399 | MPBR02 |
| | GBCD08 | 7734 | MPBAR01 | MK303004 | - | - | - |
| | GBCD09 | 7735 | MPBAR01 | MK303005 | MPR502 | MN790400 | MPBR02 |
| | GBCD10 | 7736 | MPBAR01 | MK303006 | MPR502 | MN790401 | MPBR02 |
| | GBCD11 | 7737 | MPBAR01 | MK303007 | MPR502 | MN790402 | MPBR02 |
| | GBCD12 | 7738 | MPBAR01 | MK303008 | MPR502 | MN790403 | MPBR02 |
| | GBCD13 | 7739 | MPBAR01 | MK303009 | MPR502 | MN790404 | MPBR02 |
| | GBCD14 | 7740 | MPBAR01 | MK303010 | MPR502 | MN790405 | MPBR02 |
| | GBCD15 | 7741 | MPBAR01 | MK303011 | MPR502 | MN790406 | MPBR02 |
| | GBCD16 | 7742 | MPBAR01 | MK303012 | MPR502 | MN790407 | MPBR02 |

| | | | | | | | |
|---|---|---|---|---|---|---|---|
| | GBCD17 | 7743 | MPBAR01 | MK303013 | MPR502 | MN790408 | MPBR02 |
| | GBCD18 | 7744 | MPBAR01 | MK303014 | - | - | - |
| | GBCD19 | 7745 | MPBAR01 | MK303015 | MPR502 | MN790409 | MPBR02 |
| | GBCD21 | 7746 | MPBAR01 | MK303016 | MPR502 | MN790410 | MPBR02 |
| | GBCD22 | 7747 | MPBAR01 | MK303017 | MPR502 | MN790411 | MPBR02 |
| 15. Chilgok, GB, Korea (GBCGA03 – GBCGA21, Jun. 28, 2013/ 7; GBCGB01 – GBCGB37, Jul. 12, 2018/ 20) | GBCGA03 | 7748 | MPBAR01 | MK303018 | - | - | - |
| | GBCGA04 | 7749 | MPBAR01 | MK303019 | - | - | - |
| | GBCGA05 | 7750 | MPBAR01 | MK303020 | - | - | - |
| | GBCGA06 | 7751 | MPBAR01 | MK303021 | - | - | - |
| | GBCGA07 | 7752 | MPBAR02 | MK303022 | - | - | - |
| | GBCGA11 | 7753 | MPBAR01 | MK303023 | - | - | - |
| | GBCGA21 | 7754 | MPBAR01 | MK303024 | - | - | - |
| | GBCGB01 | 7755 | MPBAR01 | MK303025 | - | - | - |
| | GBCGB02 | 7756 | MPBAR01 | MK303026 | - | - | - |
| | GBCGB03 | 7757 | MPBAR01 | MK303027 | - | - | - |
| | GBCGB04 | 7758 | MPBAR01 | MK303028 | - | - | - |
| | GBCGB09 | 7759 | MPBAR01 | MK303029 | - | - | - |
| | GBCGB14 | 7760 | MPBAR01 | MK303030 | - | - | - |
| | GBCGB15 | 7761 | MPBAR01 | MK303031 | - | - | - |
| | GBCGB16 | 7762 | MPBAR01 | MK303032 | - | - | - |
| | GBCGB17 | 7763 | MPBAR01 | MK303033 | - | - | - |
| | GBCGB18 | 7764 | MPBAR01 | MK303034 | - | - | - |
| | GBCGB19 | 7765 | MPBAR01 | MK303035 | - | - | - |
| | GBCGB21 | 7766 | MPBAR01 | MK303036 | - | - | - |
| | GBCGB22 | 7767 | MPBAR01 | MK303037 | - | - | - |
| | GBCGB23 | 7768 | MPBAR01 | MK303038 | - | - | - |
| | GBCGB27 | 7769 | MPBAR01 | MK303039 | - | - | - |
| | GBCGB30 | 7770 | MPBAR01 | MK303040 | - | - | - |
| | GBCGB33 | 7771 | MPBAR01 | MK303041 | - | - | - |
| | GBCGB35 | 7772 | MPBAR01 | MK303042 | - | - | - |
| | GBCGB36 | 7773 | MPBAR01 | MK303043 | - | - | - |
| | GBCGB37 | 7774 | MPBAR01 | MK303044 | - | - | - |
| 16. Sangju, GB, Korea (Jul. 24, 2018/ 24) | GBSJ01 | 10619 | MPBAR01 | MN790205 | MPR502 | MN790412 | MPBR02 |
| | GBSJ02 | 10620 | MPBAR01 | MN790206 | MPR502 | MN790413 | MPBR02 |
| | GBSJ03 | 10621 | MPBAR01 | MN790207 | MPR501 | MN790414 | MPBR01 |
| | GBSJ04 | 10622 | MPBAR01 | MN790208 | MPR502 | MN790415 | MPBR02 |
| | GBSJ05 | 10623 | MPBAR01 | MN790209 | MPR501 | MN790416 | MPBR01 |
| | GBSJ06 | 10624 | MPBAR01 | MN790210 | MPR504 | MN790417 | MPBR04 |
| | GBSJ07 | 10625 | MPBAR01 | MN790211 | MPR502 | MN790418 | MPBR02 |
| | GBSJ08 | 10626 | MPBAR01 | MN790212 | MPR502 | MN790419 | MPBR02 |
| | GBSJ09 | 10627 | MPBAR01 | MN790213 | MPR507 | MN790420 | MPBR07 |
| | GBSJ10 | 10628 | MPBAR01 | MN790214 | MPR501 | MN790421 | MPBR01 |
| | GBSJ11 | 10629 | MPBAR01 | MN790215 | MPR501 | MN790422 | MPBR01 |
| | GBSJ12 | 10630 | MPBAR01 | MN790216 | MPR502 | MN790423 | MPBR02 |
| | GBSJ13 | 9900 | MPBAR01 | MN790217 | MPR502 | MN790424 | MPBR02 |
| | GBSJ14 | 9901 | MPBAR01 | MN790218 | MPR502 | MN790425 | MPBR02 |
| | GBSJ15 | 9902 | MPBAR01 | MN790219 | - | - | - |
| | GBSJ16 | 9903 | MPBAR01 | MN790220 | MPR502 | MN790426 | MPBR02 |
| | GBSJ17 | 9904 | MPBAR01 | MN790221 | - | - | - |
| | GBSJ18 | 9905 | MPBAR01 | MN790222 | MPR501 | MN790427 | MPBR01 |
| | GBSJ19 | 9906 | MPBAR01 | MN790223 | MPR502 | MN790428 | MPBR02 |
| | GBSJ20 | 9907 | MPBAR01 | MN790224 | - | - | - |
| | GBSJ21 | 9908 | MPBAR01 | MN790225 | MPR501 | MN790429 | MPBR01 |
| | GBSJ22 | 9909 | MPBAR01 | MN790226 | MPR501 | MN790430 | MPBR01 |
| | GBSJ23 | 9910 | MPBAR01 | MN790227 | MPR502 | MN790431 | MPBR02 |
| | GBSJ24 | 9911 | MPBAR01 | MN790228 | MPR502 | MN790432 | MPBR02 |
| 17. Yeongju, GB, Korea (Jul. 31, 2018/ 12) | GBYJ01 | 10607 | MPBAR01 | MN790229 | MPR501 | MN790433 | MPBR01 |
| | GBYJ02 | 10608 | MPBAR01 | MN790230 | MPR501 | MN790434 | MPBR01 |
| | GBYJ03 | 10609 | MPBAR01 | MN790231 | MPR501 | MN790435 | MPBR01 |
| | GBYJ04 | 10610 | MPBAR01 | MN790232 | MPR502 | MN790436 | MPBR02 |
| | GBYJ05 | 10611 | MPBAR01 | MN790233 | MPR501 | MN790437 | MPBR01 |

| | | | | | | | |
|---|---|---|---|---|---|---|---|
| | GBYJ06 | 10612 | MPBAR02 | MN790234 | MPR503 | MN790438 | MPBR16 |
| | GBYJ07 | 10613 | MPBAR02 | MN790235 | MPR503 | MN790439 | MPBR16 |
| | GBYJ08 | 10614 | MPBAR01 | MN790236 | MPR501 | MN790440 | MPBR01 |
| | GBYJ09 | 10615 | MPBAR01 | MN790237 | MPR505 | MN790441 | MPBR05 |
| | GBYJ10 | 10616 | MPBAR01 | MN790238 | MPR501 | MN790442 | MPBR01 |
| | GBYJ11 | 10617 | MPBAR01 | MN790239 | MPR501 | MN790443 | MPBR01 |
| | GBYJ12 | 10618 | MPBAR01 | MN790240 | MPR501 | MN790444 | MPBR01 |
| 18. Gimhae, GN, Korea (Jun. 24, 2018/ 18) | GNGHB02 | 7775 | MPBAR01 | MK303045 | MPR502 | MN790445 | MPBR02 |
| | GNGHB03 | 7776 | MPBAR01 | MK303046 | - | - | - |
| | GNGHB05 | 7777 | MPBAR01 | MK303047 | MPR502 | MN790446 | MPBR02 |
| | GNGHB06 | 7778 | MPBAR01 | MK303048 | MPR502 | MN790447 | MPBR02 |
| | GNGHB07 | 7779 | MPBAR01 | MK303049 | - | - | - |
| | GNGHB08 | 7780 | MPBAR01 | MK303050 | MPR502 | MN790448 | MPBR02 |
| | GNGHB09 | 7781 | MPBAR01 | MK303051 | MPR501 | MN790449 | MPBR01 |
| | GNGHB10 | 7782 | MPBAR01 | MK303052 | MPR502 | MN790450 | MPBR02 |
| | GNGHB11 | 7783 | MPBAR02 | MK303053 | MPR503 | MN790451 | MPBR16 |
| | GNGHB12 | 7784 | MPBAR01 | MK303054 | MPR502 | MN790452 | MPBR02 |
| | GNGHB13 | 7785 | MPBAR01 | MK303055 | MPR501 | MN790453 | MPBR01 |
| | GNGHB14 | 7786 | MPBAR01 | MK303056 | MPR501 | MN790454 | MPBR01 |
| | GNGHB15 | 7787 | MPBAR01 | MK303057 | MPR501 | MN790455 | MPBR01 |
| | GNGHB16 | 7788 | MPBAR01 | MK303058 | MPR501 | MN790456 | MPBR01 |
| | GNGHB17 | 7789 | MPBAR01 | MK303059 | MPR502 | MN790457 | MPBR02 |
| | GNGHB18 | 7790 | MPBAR01 | MK303060 | MPR501 | MN790458 | MPBR01 |
| | GNGHB19 | 7791 | MPBAR01 | MK303061 | MPR501 | MN790459 | MPBR01 |
| | GNGHB20 | 7792 | MPBAR01 | MK303062 | MPR501 | MN790460 | MPBR01 |
| 19. Iksan, JB, Korea (Jun. 18, 2013/ 20) | JBYS01 | 7873 | MPBAR01 | MK303143 | MPR501 | MN790461 | MPBR01 |
| | JBYS02 | 7874 | MPBAR01 | MK303144 | MPR501 | MN790462 | MPBR01 |
| | JBYS03 | 7875 | MPBAR01 | MK303145 | MPR501 | MN790463 | MPBR01 |
| | JBYS04 | 7876 | MPBAR01 | MK303146 | MPR501 | MN790464 | MPBR01 |
| | JBYS05 | 7877 | MPBAR01 | MK303147 | MPR501 | MN790465 | MPBR01 |
| | JBYS06 | 7878 | MPBAR01 | MK303148 | MPR501 | MN790466 | MPBR01 |
| | JBYS07 | 7879 | MPBAR01 | MK303149 | MPR501 | MN790467 | MPBR01 |
| | JBYS08 | 7880 | MPBAR01 | MK303150 | MPR501 | MN790468 | MPBR01 |
| | JBYS09 | 7881 | MPBAR01 | MK303151 | MPR501 | MN790469 | MPBR01 |
| | JBYS10 | 7882 | MPBAR01 | MK303152 | MPR501 | MN790470 | MPBR01 |
| | JBYS11 | 7883 | MPBAR01 | MK303153 | MPR501 | MN790471 | MPBR01 |
| | JBYS12 | 7884 | MPBAR01 | MK303154 | MPR501 | MN790472 | MPBR01 |
| | JBYS13 | 7885 | MPBAR01 | MK303155 | MPR501 | MN790473 | MPBR01 |
| | JBYS14 | 7886 | MPBAR01 | MK303156 | MPR501 | MN790474 | MPBR01 |
| | JBYS15 | 7887 | MPBAR01 | MK303157 | MPR501 | MN790475 | MPBR01 |
| | JBYS16 | 7888 | MPBAR01 | MK303158 | MPR501 | MN790476 | MPBR01 |
| | JBYS17 | 7889 | MPBAR01 | MK303159 | MPR501 | MN790477 | MPBR01 |
| | JBYS18 | 7890 | MPBAR01 | MK303160 | MPR501 | MN790478 | MPBR01 |
| | JBYS19 | 7891 | MPBAR01 | MK303161 | MPR501 | MN790479 | MPBR01 |
| | JBYS20 | 7892 | MPBAR01 | MK303162 | MPR501 | MN790480 | MPBR01 |
| 20. Gokseong, JN, Korea (Jul. 13, 2018/ 25) | JNGS01 | 7893 | MPBAR01 | MK303163 | - | - | - |
| | JNGS02 | 7894 | MPBAR01 | MK303164 | - | - | - |
| | JNGS03 | 7895 | MPBAR01 | MK303165 | - | - | - |
| | JNGS04 | 7896 | MPBAR01 | MK303166 | - | - | - |
| | JNGS05 | 7897 | MPBAR01 | MK303167 | - | - | - |
| | JNGS06 | 7898 | MPBAR01 | MK303168 | - | - | - |
| | JNGS07 | 7899 | MPBAR01 | MK303169 | - | - | - |
| | JNGS08 | 7900 | MPBAR01 | MK303170 | - | - | - |
| | JNGS10 | 7901 | MPBAR01 | MK303171 | - | - | - |
| | JNGS11 | 7902 | MPBAR01 | MK303172 | - | - | - |
| | JNGS12 | 7903 | MPBAR01 | MK303173 | - | - | - |
| | JNGS13 | 7904 | MPBAR01 | MK303174 | - | - | - |
| | JNGS14 | 7905 | MPBAR01 | MK303175 | - | - | - |
| | JNGS15 | 7906 | MPBAR01 | MK303176 | - | - | - |
| | JNGS16 | 7907 | MPBAR01 | MK303177 | - | - | - |
| | JNGS18 | 7908 | MPBAR01 | MK303178 | - | - | - |

| | | | | | | | |
|---|---|---|---|---|---|---|---|
| | JNGS19 | 7909 | MPBAR01 | MK303179 | - | - | - |
| | JNGS20 | 7910 | MPBAR01 | MK303180 | - | - | - |
| | JNGS21 | 7911 | MPBAR01 | MK303181 | - | - | - |
| | JNGS23 | 7912 | MPBAR01 | MK303182 | - | - | - |
| | JNGS24 | 7913 | MPBAR01 | MK303183 | - | - | - |
| | JNGS32 | 7914 | MPBAR01 | MK303184 | - | - | - |
| | JNGS33 | 7915 | MPBAR01 | MK303185 | - | - | - |
| | JNGS34 | 7916 | MPBAR01 | MK303186 | - | - | - |
| | JNGS35 | 7917 | MPBAR01 | MK303187 | - | - | - |
| 21. Haenam, JN, Korea (Jul. 24, 2018/ 20) | JNHN01 | 7918 | MPBAR01 | MK303188 | - | - | - |
| | JNHN02 | 7919 | MPBAR01 | MK303189 | MPR501 | MN790481 | MPBR01 |
| | JNHN03 | 7920 | MPBAR01 | MK303190 | MPR501 | MN790482 | MPBR01 |
| | JNHN04 | 7921 | MPBAR01 | MK303191 | MPR501 | MN790483 | MPBR01 |
| | JNHN05 | 7922 | MPBAR02 | MK303192 | MPR502 | MN790484 | MPBR15 |
| | JNHN06 | 7923 | MPBAR01 | MK303193 | MPR502 | MN790485 | MPBR02 |
| | JNHN07 | 7924 | MPBAR01 | MK303194 | MPR501 | MN790486 | MPBR01 |
| | JNHN08 | 7925 | MPBAR01 | MK303195 | MPR501 | MN790487 | MPBR01 |
| | JNHN09 | 7926 | MPBAR01 | MK303196 | MPR501 | MN790488 | MPBR01 |
| | JNHN10 | 7927 | MPBAR01 | MK303197 | MPR501 | MN790489 | MPBR01 |
| | JNHN11 | 7928 | MPBAR01 | MK303198 | MPR501 | MN790490 | MPBR01 |
| | JNHN12 | 7929 | MPBAR01 | MK303199 | MPR501 | MN790491 | MPBR01 |
| | JNHN13 | 7930 | MPBAR01 | MK303200 | MPR501 | MN790492 | MPBR01 |
| | JNHN14 | 7931 | MPBAR01 | MK303201 | MPR502 | MN790493 | MPBR02 |
| | JNHN15 | 7932 | MPBAR02 | MK303202 | MPR503 | MN790494 | MPBR16 |
| | JNHN16 | 7933 | MPBAR01 | MK303203 | MPR501 | MN790495 | MPBR01 |
| | JNHN17 | 7934 | MPBAR01 | MK303204 | - | - | - |
| | JNHN18 | 7935 | MPBAR01 | MK303205 | MPR501 | MN790496 | MPBR01 |
| | JNHN19 | 7936 | MPBAR01 | MK303206 | MPR501 | MN790497 | MPBR01 |
| | JNHN20 | 7937 | MPBAR01 | MK303207 | MPR501 | MN790498 | MPBR01 |
| 22. Jangseong, JN, Korea (Jul. 22, 2018/ 20) | JNJS01 | 7938 | MPBAR01 | MK303208 | MPR501 | MN790499 | MPBR01 |
| | JNJS02 | 7939 | MPBAR01 | MK303209 | MPR501 | MN790500 | MPBR01 |
| | JNJS03 | 7940 | MPBAR01 | MK303210 | MPR509 | MN790501 | MPBR09 |
| | JNJS04 | 7941 | MPBAR01 | MK303211 | MPR501 | MN790502 | MPBR01 |
| | JNJS05 | 7942 | MPBAR01 | MK303212 | MPR501 | MN790503 | MPBR01 |
| | JNJS06 | 7943 | MPBAR01 | MK303213 | MPR501 | MN790504 | MPBR01 |
| | JNJS07 | 7944 | MPBAR01 | MK303214 | MPR501 | MN790505 | MPBR01 |
| | JNJS08 | 7945 | MPBAR01 | MK303215 | MPR501 | MN790506 | MPBR01 |
| | JNJS09 | 7946 | MPBAR01 | MK303216 | MPR501 | MN790507 | MPBR01 |
| | JNJS10 | 7947 | MPBAR01 | MK303217 | MPR501 | MN790508 | MPBR01 |
| | JNJS11 | 7948 | MPBAR01 | MK303218 | MPR501 | MN790509 | MPBR01 |
| | JNJS12 | 7949 | MPBAR01 | MK303219 | MPR501 | MN790510 | MPBR01 |
| | JNJS13 | 7950 | MPBAR01 | MK303220 | MPR501 | MN790511 | MPBR01 |
| | JNJS14 | 7951 | MPBAR01 | MK303221 | MPR501 | MN790512 | MPBR01 |
| | JNJS15 | 7952 | MPBAR01 | MK303222 | MPR501 | MN790513 | MPBR01 |
| | JNJS16 | 7953 | MPBAR01 | MK303223 | MPR501 | MN790514 | MPBR01 |
| | JNJS17 | 7954 | MPBAR01 | MK303224 | MPR501 | MN790515 | MPBR01 |
| | JNJS18 | 7955 | MPBAR01 | MK303225 | MPR501 | MN790516 | MPBR01 |
| | JNJS19 | 7956 | MPBAR01 | MK303226 | MPR501 | MN790517 | MPBR01 |
| | JNJS20 | 7957 | MPBAR01 | MK303227 | MPR501 | MN790518 | MPBR01 |
| 23. Suncheon, JN, Korea (Jul. 26, 2018/ 20) | JNSC01 | 7958 | MPBAR01 | MK303228 | MPR501 | MN790519 | MPBR01 |
| | JNSC02 | 7959 | MPBAR02 | MK303229 | MPR503 | MN790520 | MPBR16 |
| | JNSC03 | 7960 | MPBAR01 | MK303230 | MPR501 | MN790521 | MPBR01 |
| | JNSC04 | 7961 | MPBAR01 | MK303231 | MPR501 | MN790522 | MPBR01 |
| | JNSC05 | 7962 | MPBAR01 | MK303232 | MPR501 | MN790523 | MPBR01 |
| | JNSC06 | 7963 | MPBAR01 | MK303233 | MPR501 | MN790524 | MPBR01 |
| | JNSC07 | 7964 | MPBAR01 | MK303234 | MPR501 | MN790525 | MPBR01 |
| | JNSC08 | 7965 | MPBAR01 | MK303235 | MPR501 | MN790526 | MPBR01 |
| | JNSC09 | 7966 | MPBAR02 | MK303236 | MPR503 | MN790527 | MPBR16 |
| | JNSC10 | 7967 | MPBAR01 | MK303237 | MPR501 | MN790528 | MPBR01 |
| | JNSC11 | 7968 | MPBAR01 | MK303238 | MPR501 | MN790529 | MPBR01 |
| | JNSC12 | 7969 | MPBAR01 | MK303239 | - | - | - |

| | | | | | | | |
|---|---|---|---|---|---|---|---|
| | JNSC13 | 7970 | MPBAR01 | MK303240 | MPR501 | MN790530 | MPBR01 |
| | JNSC14 | 7971 | MPBAR01 | MK303241 | MPR501 | MN790531 | MPBR01 |
| | JNSC15 | 7972 | MPBAR01 | MK303242 | MPR505 | MN790532 | MPBR05 |
| | JNSC16 | 7973 | MPBAR01 | MK303243 | MPR501 | MN790533 | MPBR01 |
| | JNSC17 | 7974 | MPBAR01 | MK303244 | MPR501 | MN790534 | MPBR01 |
| | JNSC18 | 7975 | MPBAR01 | MK303245 | MPR512 | MN790535 | MPBR11 |
| | JNSC19 | 7976 | MPBAR01 | MK303246 | MPR501 | MN790536 | MPBR01 |
| | JNSC20 | 7977 | MPBAR01 | MK303247 | MPR501 | MN790537 | MPBR01 |
| 24. San Remo, Italy (Aug. 09, 2012/ 4) | ITA02 | 8002 | MPBAR01 | MK303272 | MPR502 | MN790538 | MPBR02 |
| | ITA03 | 8003 | MPBAR02 | MK303273 | MPR503 | MN790539 | MPBR16 |
| | ITA07 | 8004 | MPBAR01 | MK303274 | MPR501 | MN790540 | MPBR01 |
| | ITA08 | 8005 | MPBAR02 | MK303275 | MPR503 | MN790541 | MPBR16 |
| 25. Savona, Italy (Aug. 10, 2012/ 3) | ITA10 | 8006 | MPBAR01 | MK303276 | MPR501 | MN790542 | MPBR01 |
| | ITA11 | 8007 | MPBAR01 | MK303277 | MPR501 | MN790543 | MPBR01 |
| | ITA12 | 8008 | MPBAR02 | MK303278 | MPR503 | MN790544 | MPBR16 |
| 26. Borghetto, Italy (Aug. 10, 2012/ 10) | ITA13 | 8009 | MPBAR01 | MK303279 | MPR501 | MN790545 | MPBR01 |
| | ITA14 | 8010 | MPBAR01 | MK303280 | MPR501 | MN790546 | MPBR01 |
| | ITA15 | 8011 | MPBAR01 | MK303281 | MPR501 | MN790547 | MPBR01 |
| | ITA16 | 8012 | MPBAR01 | MK303282 | MPR501 | MN790548 | MPBR01 |
| | ITA17 | 8013 | MPBAR01 | MK303283 | MPR502 | MN790549 | MPBR02 |
| | ITA18 | 8014 | MPBAR01 | MK303284 | MPR502 | MN790550 | MPBR02 |
| | ITA19 | 8015 | MPBAR02 | MK303285 | MPR503 | MN790551 | MPBR16 |
| | ITA20 | 8016 | MPBAR01 | MK303286 | MPR502 | MN790552 | MPBR02 |
| | ITA21 | 8017 | MPBAR02 | MK303287 | MPR503 | MN790553 | MPBR16 |
| | ITA22 | 8018 | MPBAR02 | MK303288 | MPR503 | MN790554 | MPBR16 |
| 27. Genova, Italy (Aug. 10, 2012/ 5) | ITA23 | 8019 | MPBAR01 | MK303289 | MPR502 | MN790555 | MPBR02 |
| | ITA24 | 8020 | MPBAR01 | MK303290 | MPR502 | MN790556 | MPBR02 |
| | ITA25 | 8021 | MPBAR01 | MK303291 | MPR502 | MN790557 | MPBR02 |
| | ITA26 | 8022 | MPBAR01 | MK303292 | MPR502 | MN790558 | MPBR02 |
| | ITA27 | 8023 | MPBAR02 | MK303293 | MPR503 | MN790559 | MPBR16 |
| 28. Lleida, Spain (Aug. 24, 2011/ 9) | SPA02 | 8024 | MPBAR01 | MK303294 | MPR502 | MN790560 | MPBR02 |
| | SPA04 | 8025 | MPBAR01 | MK303295 | MPR502 | MN790561 | MPBR02 |
| | SPA06 | 8026 | MPBAR01 | MK303296 | MPR501 | MN790562 | MPBR01 |
| | SPA07 | 8027 | MPBAR01 | MK303297 | MPR502 | MN790563 | MPBR02 |
| | SPA08 | 8028 | MPBAR01 | MK303298 | MPR501 | MN790564 | MPBR01 |
| | SPA10 | 8029 | MPBAR01 | MK303299 | MPR502 | MN790565 | MPBR02 |
| | SPA13 | 8030 | MPBAR01 | MK303300 | MPR502 | MN790566 | MPBR02 |
| | SPA14 | 8031 | MPBAR01 | MK303301 | MPR501 | MN790567 | MPBR01 |
| | SPA15 | 8032 | MPBAR01 | MK303302 | MPR514 | MN790568 | MPBR13 |
| 29. INRA, France (Aug. 10, 2012/ 1) | FRA21 | 8033 | MPBAR01 | MK303303 | MPR501 | MN790569 | MPBR01 |
| 30. Montpellie, France (FRA27 – FRA30, Aug. 10, 2012/ 3; FRA31 – FRA37, Aug. 10, 2012/ 6) | FRA27 | 8034 | MPBAR02 | MK303304 | MPR503 | MN790570 | MPBR16 |
| | FRA29 | 8036 | MPBAR02 | MK303306 | MPR503 | MN790571 | MPBR16 |
| | FRA30 | 8037 | MPBAR02 | MK303307 | MPR503 | MN790572 | MPBR16 |
| | FRA31 | 8038 | MPBAR02 | MK303308 | MPR503 | MN790573 | MPBR16 |
| | FRA32 | 8039 | MPBAR02 | MK303309 | MPR503 | MN790574 | MPBR16 |
| | FRA33 | 8040 | MPBAR01 | MK303310 | MPR501 | MN790575 | MPBR01 |
| | FRA34 | 8041 | MPBAR01 | MK303311 | MPR508 | MN790576 | MPBR08 |
| | FRA36 | 8042 | MPBAR01 | MK303312 | MPR502 | MN790577 | MPBR02 |
| | FRA37 | 8043 | MPBAR02 | MK303313 | MPR503 | MN790578 | MPBR16 |
| 31. ARS, France (Aug. 10, 2012/ 4) | FRA38 | 8044 | MPBAR01 | MK303314 | MPR513 | MN790579 | MPBR12 |
| | FRA39 | 8045 | MPBAR01 | MK303315 | MPR501 | MN790580 | MPBR01 |
| | FRA40 | 8046 | MPBAR02 | MK303316 | MPR515 | MN790581 | MPBR19 |
| | FRA41 | 8047 | MPBAR01 | MK303317 | MPR501 | MN790582 | MPBR01 |

GG, Gyeonggi-do Province; GW, Gangwon-do Province; CB, Chungcheongbuk-do Province; CN, Chungcheongnam-do Province; GB, Gyeongsangbuk-do Province; GN, Gyeongsangnam-do Province; JB, Jeollabuk-do Province; and JN, Jeollanam-do Province.
INRA, The French National Institute for Agricultural Research; and ARS, Ars-sur-Formans.
-, DNA barcoding region was analyzed; however, Region 5 was not.
*The haplotypes MPBAR01, MPBAR02, and MPBAR03 were defined based on 658 bp of the DNA barcoding region in this study corresponding to the haplotypes H1, H3, and H9, respectively, which were defined based on 472 bp of the DNA barcoding region in Park et al. (2016)

**Table S2.** List of primers used to amplify the variable regions detected during the comparison between two mitochondrial genomes of *Metcalfa pruinosa*

| Name | Location | Primer name | Direction[a] | Sequence (5'-3') |
|---|---|---|---|---|
| Region 1 | *ND2– COI*[b] | Region01-F1 | F | TTATGAGATTTGTTTACATC |
| | | Region01-F2 | F | CAATCAATTACACCAGTATC |
| | | Region01-R1 | R | ACGTTGATAATGAGGTGGCTG |
| | | Region01-R2 | R | TAAAATACAGAGTTCCGATGTC |
| Region 2 | *COI* | Region02-F1 | F | TTTCACATATTATTATACACG |
| | | Region02-F2 | F | TAATTTACGCTATAATCT |
| | | Region02-R1 | R | TAAAAATACAAATCCTATTGATC |
| | | Region02-R2 | R | ACATCAATTGATGAGTTTGC |
| Region 3 | *ATP6* | Region03-F1 | F | AACTTTACAGCTACTAGTCA |
| | | Region03-F2 | F | CATTAGCTCTACCAACATGACT |
| | | Region03-R1 | R | CTTGAATAATTGAGATTGA |
| | | Region03-R2 | R | AGTGTTGAAAATACATATGC |
| Region 4 | *ND5* | Region08-F1 | F | TACTTTAGTAACTTCCGGTG |
| | | Region08-F2 | F | GGTGTTTATTTACTTATTCGA |
| | | Region08-R1 | R | ATTGACTTAAAAATAGCATG |
| | | Region08-R2 | R | AGAAAGTCCACCCATATAACG |
| Region 5 | A+T–rich region | Region11-F1 | F | ATTAATTAAAATGCGTTGATC |
| | | Region11-F2 | F | TAGTTAATAATTATTAATACTG |
| | | Region11-R1 | R | CAATATATAATCTAAGTTATAC |
| | | Region11-R2 | R | AGAAATCATATTTTAATTATCAG |

[a]F and R, forward and reverse direction of transcription, respectively.

[b]Gap between *ND2* and *trnW*

**Table 3.** List of worldwide *COI* sequences of *Metcalfa pruinosa* (830 individuals), including those obtained in this study

| Locality | GenBank animal number | Sample number | *COI* haplotype (470 bp) | GenBank no. | Reference |
|---|---|---|---|---|---|
| 1. Seoul, Korea (42) | SSW01 | 7978 | MPH01 | MK303248 | This study |
| | SSW02 | 7979 | MPH01 | MK303249 | This study |
| | SSW03 | 7980 | MPH01 | MK303250 | This study |
| | SSW04 | 7981 | MPH01 | MK303251 | This study |
| | SSW05 | 7982 | MPH01 | MK303252 | This study |
| | SSW06 | 7983 | MPH01 | MK303253 | This study |
| | SSW07 | 7984 | MPH01 | MK303254 | This study |
| | SSW08 | 7985 | MPH01 | MK303255 | This study |
| | SSW10 | 7986 | MPH01 | MK303256 | This study |
| | SSW12 | 7987 | MPH01 | MK303257 | This study |
| | SSW14 | 7988 | MPH01 | MK303258 | This study |
| | SSW15 | 7989 | MPH01 | MK303259 | This study |
| | SSW16 | 7990 | MPH01 | MK303260 | This study |
| | SSW17 | 7991 | MPH02 | MK303261 | This study |
| | SSW18 | 7992 | MPH01 | MK303262 | This study |
| | SSW19 | 7993 | MPH01 | MK303263 | This study |
| | SSW20 | 7994 | MPH02 | MK303264 | This study |
| | SSW23 | 7995 | MPH01 | MK303265 | This study |
| | SSW25 | 7996 | MPH01 | MK303266 | This study |
| | SSW27 | 7997 | MPH01 | MK303267 | This study |
| | SSW28 | 7998 | MPH01 | MK303268 | This study |
| | SSW29 | 7999 | MPH01 | MK303269 | This study |
| | SSW30 | 8000 | MPH01 | MK303270 | This study |
| | SSW31 | 8001 | MPH01 | MK303271 | This study |
| | 13-Seoul-01 | | MPH01 | KT382644 | Park et al. (2016) |
| | 13-Seoul-02 | | MPH01 | KT382645 | Park et al. (2016) |
| | 13-Seoul-03 | | MPH01 | KT382646 | Park et al. (2016) |
| | 13-Seoul-04 | | MPH01 | KT382647 | Park et al. (2016) |
| | 13-Seoul-05 | | MPH01 | KT382648 | Park et al. (2016) |
| | 13-Seoul-06 | | MPH01 | KT382649 | Park et al. (2016) |
| | 13-Seoul-07 | | MPH01 | KT382650 | Park et al. (2016) |
| | 13-Seoul-08 | | MPH01 | KT382651 | Park et al. (2016) |
| | 13-Seoul-09 | | MPH01 | KT382652 | Park et al. (2016) |
| | 13-Seoul-10 | | MPH01 | KT382653 | Park et al. (2016) |
| | 13-Seoul-11 | | MPH01 | KT382654 | Park et al. (2016) |
| | 13-Seoul-12 | | MPH01 | KT382655 | Park et al. (2016) |
| | 13-Seoul-13 | | MPH01 | KT382656 | Park et al. (2016) |
| | 13-Seoul-14 | | MPH01 | KT382657 | Park et al. (2016) |
| | 13-Seoul-15 | | MPH01 | KT382658 | Park et al. (2016) |
| | 13-Seocho-01 | | MPH01 | KT382617 | Park et al. (2016) |
| | 13-Seocho-02 | | MPH01 | KT382622 | Park et al. (2016) |
| | 13-Seocho-03 | | MPH01 | KT382627 | Park et al. (2016) |
| 2. Incheon, GG, Korea (34) | IC06 | 7853 | MPH01 | MK303123 | This study |
| | IC07 | 7854 | MPH01 | MK303124 | This study |
| | IC08 | 7855 | MPH01 | MK303125 | This study |
| | IC09 | 7856 | MPH01 | MK303126 | This study |
| | IC10 | 7857 | MPH01 | MK303127 | This study |
| | IC11 | 7858 | MPH01 | MK303128 | This study |
| | IC12 | 7859 | MPH01 | MK303129 | This study |
| | IC13 | 7860 | MPH01 | MK303130 | This study |
| | IC14 | 7861 | MPH01 | MK303131 | This study |
| | IC15 | 7862 | MPH01 | MK303132 | This study |
| | IC16 | 7863 | MPH01 | MK303133 | This study |
| | IC17 | 7864 | MPH01 | MK303134 | This study |
| | IC18 | 7865 | MPH01 | MK303135 | This study |
| | IC21 | 7866 | MPH01 | MK303136 | This study |
| | IC22 | 7867 | MPH01 | MK303137 | This study |
| | IC24 | 7868 | MPH01 | MK303138 | This study |
| | IC26 | 7869 | MPH01 | MK303139 | This study |
| | IC27 | 7870 | MPH01 | MK303140 | This study |
| | IC28 | 7871 | MPH01 | MK303141 | This study |
| | IC29 | 7872 | MPH01 | MK303142 | This study |
| | 13-Incheon-01 | | MPH01 | KT382581 | Park et al. (2016) |
| | 13-Incheon-02 | | MPH01 | KT382582 | Park et al. (2016) |
| | 13-Incheon-03 | | MPH01 | KT382585 | Park et al. (2016) |
| | 13-Incheon-04 | | MPH02 | KT382586 | Park et al. (2016) |
| | 13-Incheon-05 | | MPH01 | KT382589 | Park et al. (2016) |
| | 13-Incheon-06 | | MPH01 | KT382590 | Park et al. (2016) |

| | | | | | |
|---|---|---|---|---|---|
| | 13-Incheon-07 | | MPH01 | KT382595 | Park et al. (2016) |
| | 13-Incheon-08 | | MPH02 | KT382596 | Park et al. (2016) |
| | 13-Incheon-09 | | MPH01 | KT382599 | Park et al. (2016) |
| | 13-Incheon-10 | | MPH01 | KT382600 | Park et al. (2016) |
| | 13-Incheon-11 | | MPH01 | KT382605 | Park et al. (2016) |
| | 13-Incheon-12 | | MPH02 | KT382606 | Park et al. (2016) |
| | 13-Incheon-13 | | MPH01 | KT382609 | Park et al. (2016) |
| | 13-Incheon-14 | | MPH01 | KT382610 | Park et al. (2016) |
| 3. Anseong, GG, Korea (27) | GAS01 | 7707 | MPH01 | MK302977 | This study |
| | GAS05 | 7708 | MPH01 | MK302978 | This study |
| | GAS06 | 7709 | MPH01 | MK302979 | This study |
| | GAS08 | 7710 | MPH01 | MK302980 | This study |
| | GAS09 | 7711 | MPH01 | MK302981 | This study |
| | GAS10 | 7712 | MPH02 | MK302982 | This study |
| | GAS11 | 7713 | MPH01 | MK302983 | This study |
| | GAS12 | 7714 | MPH01 | MK302984 | This study |
| | GAS13 | 7715 | MPH02 | MK302985 | This study |
| | GAS14 | 7716 | MPH01 | MK302986 | This study |
| | GAS15 | 7717 | MPH01 | MK302987 | This study |
| | GAS17 | 7718 | MPH01 | MK302988 | This study |
| | GAS18 | 7719 | MPH01 | MK302989 | This study |
| | GAS19 | 7720 | MPH01 | MK302990 | This study |
| | GAS20 | 7721 | MPH01 | MK302991 | This study |
| | GAS24 | 7722 | MPH01 | MK302992 | This study |
| | GAS25 | 7723 | MPH01 | MK302993 | This study |
| | GAS26 | 7724 | MPH01 | MK302994 | This study |
| | GAS28 | 7725 | MPH01 | MK302995 | This study |
| | GAS29 | 7726 | MPH01 | MK302996 | This study |
| | GAS30 | 7727 | MPH01 | MK302997 | This study |
| | 13-Anseong-01 | | MPH01 | KT382620 | Park et al. (2016) |
| | 13-Anseong-02 | | MPH01 | KT382621 | Park et al. (2016) |
| | 13-Anseong-03 | | MPH01 | KT382625 | Park et al. (2016) |
| | 13-Anseong-04 | | MPH01 | KT382626 | Park et al. (2016) |
| | 13-Anseong-05 | | MPH01 | KT382630 | Park et al. (2016) |
| | 13-Anseong-06 | | MPH01 | KT382631 | Park et al. (2016) |
| 4. Yeoju, GG, Korea (28) | GYJ02 | 10605 | MPH01 | MN790203 | This study |
| | GYJ04 | 10606 | MPH01 | MN790204 | This study |
| | GYJ06 | 7833 | MPH01 | MK303103 | This study |
| | GYJ07 | 7834 | MPH02 | MK303104 | This study |
| | GYJ08 | 7835 | MPH01 | MK303105 | This study |
| | GYJ10 | 7836 | MPH01 | MK303106 | This study |
| | GYJ11 | 7837 | MPH01 | MK303107 | This study |
| | GYJ12 | 7838 | MPH01 | MK303108 | This study |
| | GYJ13 | 7839 | MPH02 | MK303109 | This study |
| | GYJ14 | 7840 | MPH02 | MK303110 | This study |
| | GYJ15 | 7841 | MPH02 | MK303111 | This study |
| | GYJ16 | 7842 | MPH01 | MK303112 | This study |
| | GYJ17 | 7843 | MPH01 | MK303113 | This study |
| | GYJ18 | 7844 | MPH03 | MK303114 | This study |
| | GYJ19 | 7845 | MPH02 | MK303115 | This study |
| | GYJ20 | 7846 | MPH01 | MK303116 | This study |
| | GYJ21 | 7847 | MPH02 | MK303117 | This study |
| | GYJ23 | 7848 | MPH01 | MK303118 | This study |
| | GYJ24 | 7849 | MPH02 | MK303119 | This study |
| | GYJ25 | 7850 | MPH02 | MK303120 | This study |
| | GYJ26 | 7851 | MPH01 | MK303121 | This study |
| | GYJ29 | 7852 | MPH01 | MK303122 | This study |
| | 13-Yeoju-01 | | MPH01 | KT382583 | Park et al. (2016) |
| | 13-Yeoju-02 | | MPH03 | KT382584 | Park et al. (2016) |
| | 13-Yeoju-03 | | MPH01 | KT382593 | Park et al. (2016) |
| | 13-Yeoju-04 | | MPH03 | KT382594 | Park et al. (2016) |
| | 13-Yeoju-05 | | MPH01 | KT382603 | Park et al. (2016) |
| | 13-Yeoju-06 | | MPH03 | KT382604 | Park et al. (2016) |
| 5. Paju, GG, Korea (6) | 13-Paju-01 | | MPH01 | KT382587 | Park et al. (2016) |
| | 13-Paju-02 | | MPH02 | KT382588 | Park et al. (2016) |
| | 13-Paju-03 | | MPH01 | KT382597 | Park et al. (2016) |
| | 13-Paju-04 | | MPH02 | KT382598 | Park et al. (2016) |
| | 13-Paju-05 | | MPH01 | KT382607 | Park et al. (2016) |
| | 13-Paju-06 | | MPH02 | KT382608 | Park et al. (2016) |
| 6. Icheon, GG, Korea (4) | 13-Icheon-01 | | MPH01 | KT382591 | Park et al. (2016) |
| | 13-Icheon-02 | | MPH01 | KT382592 | Park et al. (2016) |
| | 13-Ichoen-03 | | MPH01 | KT382601 | Park et al. (2016) |

| | | | | | |
|---|---|---|---|---|---|
| | 13-Ichoen-04 | | MPH01 | KT382602 | Park et al. (2016) |
| 7. Hwaseong, GG, Korea (6) | 13-Hwaseong-01 | | MPH01 | KT382611 | Park et al. (2016) |
| | 13-Hwaseong-02 | | MPH01 | KT382612 | Park et al. (2016) |
| | 13-Hwaseong-03 | | MPH01 | KT382613 | Park et al. (2016) |
| | 13-Hwaseong-04 | | MPH01 | KT382614 | Park et al. (2016) |
| | 13-Hwaseong-05 | | MPH01 | KT382615 | Park et al. (2016) |
| | 13-Hwaseong-06 | | MPH01 | KT382616 | Park et al. (2016) |
| 8. Gwacheon, GG, Korea (6) | 13-Gwacheon-01 | | MPH01 | KT382618 | Park et al. (2016) |
| | 13-Gwacheon-02 | | MPH01 | KT382619 | Park et al. (2016) |
| | 13-Gwacheon-03 | | MPH01 | KT382623 | Park et al. (2016) |
| | 13-Gwacheon-04 | | MPH01 | KT382624 | Park et al. (2016) |
| | 13-Gwacheon-05 | | MPH01 | KT382628 | Park et al. (2016) |
| | 13-Gwacheon-06 | | MPH01 | KT382629 | Park et al. (2016) |
| 9. Yongin, GG, Korea (6) | 13-Yongin-01 | | MPH01 | KT382632 | Park et al. (2016) |
| | 13-Yongin-02 | | MPH01 | KT382633 | Park et al. (2016) |
| | 13-Yongin-03 | | MPH01 | KT382636 | Park et al. (2016) |
| | 13-Yongin-04 | | MPH01 | KT382637 | Park et al. (2016) |
| | 13-Yongin-05 | | MPH01 | KT382640 | Park et al. (2016) |
| | 13-Yongin-06 | | MPH01 | KT382641 | Park et al. (2016) |
| 10. Suwon, GG, Korea (6) | 13-Suwon-01 | | MPH01 | KT382634 | Park et al. (2016) |
| | 13-Suwon-02 | | MPH01 | KT382635 | Park et al. (2016) |
| | 13-Suwon-03 | | MPH01 | KT382638 | Park et al. (2016) |
| | 13-Suwon-04 | | MPH01 | KT382639 | Park et al. (2016) |
| | 13-Suwon-05 | | MPH01 | KT382642 | Park et al. (2016) |
| | 13-Suwon-06 | | MPH01 | KT382643 | Park et al. (2016) |
| 11. Pyeongchang, GW, Korea (20) | GWPC01 | 7793 | MPH01 | MK303063 | This study |
| | GWPC02 | 7794 | MPH01 | MK303064 | This study |
| | GWPC03 | 7795 | MPH01 | MK303065 | This study |
| | GWPC04 | 7796 | MPH01 | MK303066 | This study |
| | GWPC05 | 7797 | MPH02 | MK303067 | This study |
| | GWPC06 | 7798 | MPH02 | MK303068 | This study |
| | GWPC07 | 7799 | MPH01 | MK303069 | This study |
| | GWPC08 | 7800 | MPH01 | MK303070 | This study |
| | GWPC09 | 7801 | MPH01 | MK303071 | This study |
| | GWPC10 | 7802 | MPH02 | MK303072 | This study |
| | GWPC11 | 7803 | MPH01 | MK303073 | This study |
| | GWPC12 | 7804 | MPH02 | MK303074 | This study |
| | GWPC13 | 7805 | MPH01 | MK303075 | This study |
| | GWPC14 | 7806 | MPH01 | MK303076 | This study |
| | GWPC15 | 7807 | MPH01 | MK303077 | This study |
| | GWPC16 | 7808 | MPH01 | MK303078 | This study |
| | GWPC17 | 7809 | MPH01 | MK303079 | This study |
| | GWPC18 | 7810 | MPH01 | MK303080 | This study |
| | GWPC19 | 7811 | MPH01 | MK303081 | This study |
| | GWPC20 | 7812 | MPH01 | MK303082 | This study |
| 12. Wonju, GW, Korea (26) | GWWJ01 | 7813 | MPH01 | MK303083 | This study |
| | GWWJ02 | 7814 | MPH01 | MK303084 | This study |
| | GWWJ03 | 7815 | MPH01 | MK303085 | This study |
| | GWWJ04 | 7816 | MPH01 | MK303086 | This study |
| | GWWJ05 | 7817 | MPH01 | MK303087 | This study |
| | GWWJ06 | 7818 | MPH01 | MK303088 | This study |
| | GWWJ07 | 7819 | MPH01 | MK303089 | This study |
| | GWWJ08 | 7820 | MPH01 | MK303090 | This study |
| | GWWJ09 | 7821 | MPH01 | MK303091 | This study |
| | GWWJ10 | 7822 | MPH01 | MK303092 | This study |
| | GWWJ11 | 7823 | MPH01 | MK303093 | This study |
| | GWWJ12 | 7824 | MPH01 | MK303094 | This study |
| | GWWJ13 | 7825 | MPH01 | MK303095 | This study |
| | GWWJ14 | 7826 | MPH01 | MK303096 | This study |
| | GWWJ15 | 7827 | MPH01 | MK303097 | This study |
| | GWWJ16 | 7828 | MPH01 | MK303098 | This study |
| | GWWJ17 | 7829 | MPH01 | MK303099 | This study |
| | GWWJ18 | 7830 | MPH01 | MK303100 | This study |
| | GWWJ19 | 7831 | MPH01 | MK303101 | This study |
| | GWWJ20 | 7832 | MPH01 | MK303102 | This study |
| | 1-Wonju-01 | | MPH01 | KT382659 | Park et al. (2016) |
| | 1-Wonju-02 | | MPH01 | KT382660 | Park et al. (2016) |
| | 1-Wonju-03 | | MPH01 | KT382661 | Park et al. (2016) |
| | 1-Wonju-04 | | MPH01 | KT382662 | Park et al. (2016) |
| | 1-Wonju-05 | | MPH01 | KT382663 | Park et al. (2016) |
| | 1-Wonju-06 | | MPH01 | KT382664 | Park et al. (2016) |
| 13. Danyang, CB, Korea (29) | CBDY01 | 7549 | MPH01 | MK302819 | This study |

| | | | | | |
|---|---|---|---|---|---|
| | CBDY02 | 7550 | MPH01 | MK302820 | This study |
| | CBDY03 | 7551 | MPH01 | MK302821 | This study |
| | CBDY05 | 7552 | MPH01 | MK302822 | This study |
| | CBDY06 | 7553 | MPH01 | MK302823 | This study |
| | CBDY07 | 7554 | MPH02 | MK302824 | This study |
| | CBDY08 | 7555 | MPH01 | MK302825 | This study |
| | CBDY09 | 7556 | MPH01 | MK302826 | This study |
| | CBDY10 | 7557 | MPH01 | MK302827 | This study |
| | CBDY11 | 7558 | MPH01 | MK302828 | This study |
| | CBDY13 | 7559 | MPH01 | MK302829 | This study |
| | CBDY14 | 7560 | MPH01 | MK302830 | This study |
| | CBDY16 | 7561 | MPH01 | MK302831 | This study |
| | CBDY17 | 7562 | MPH01 | MK302832 | This study |
| | CBDY19 | 7563 | MPH01 | MK302833 | This study |
| | CBDY20 | 7564 | MPH01 | MK302834 | This study |
| | CBDY22 | 7565 | MPH01 | MK302835 | This study |
| | CBDY23 | 7566 | MPH01 | MK302836 | This study |
| | CBDY25 | 7567 | MPH01 | MK302837 | This study |
| | CBDY26 | 7568 | MPH01 | MK302838 | This study |
| | CBDY27 | 7569 | MPH01 | MK302839 | This study |
| | CBDY30 | 7570 | MPH01 | MK302840 | This study |
| | CBDY31 | 7571 | MPH01 | MK302841 | This study |
| | 13-Danyang-01 | | MPH01 | KT382665 | Park et al. (2016) |
| | 13-Danyang-02 | | MPH01 | KT382666 | Park et al. (2016) |
| | 13-Danyang-03 | | MPH01 | KT382671 | Park et al. (2016) |
| | 13-Danyang-04 | | MPH01 | KT382672 | Park et al. (2016) |
| | 13-Danyang-05 | | MPH01 | KT382677 | Park et al. (2016) |
| | 13-Danyang-06 | | MPH01 | KT382678 | Park et al. (2016) |
| 14. Goesan, CB, Korea (27) | CBGS01 | 7572 | MPH01 | MK302842 | This study |
| | CBGS02 | 7573 | MPH01 | MK302843 | This study |
| | CBGS03 | 7574 | MPH01 | MK302844 | This study |
| | CBGS04 | 7575 | MPH01 | MK302845 | This study |
| | CBGS06 | 7576 | MPH01 | MK302846 | This study |
| | CBGS09 | 7577 | MPH01 | MK302847 | This study |
| | CBGS10 | 7578 | MPH01 | MK302848 | This study |
| | CBGS11 | 7579 | MPH01 | MK302849 | This study |
| | CBGS14 | 7580 | MPH01 | MK302850 | This study |
| | CBGS17 | 7581 | MPH01 | MK302851 | This study |
| | CBGS18 | 7582 | MPH01 | MK302852 | This study |
| | CBGS19 | 7583 | MPH01 | MK302853 | This study |
| | CBGS20 | 7584 | MPH01 | MK302854 | This study |
| | CBGS22 | 7585 | MPH01 | MK302855 | This study |
| | CBGS23 | 7586 | MPH01 | MK302856 | This study |
| | CBGS24 | 7587 | MPH01 | MK302857 | This study |
| | CBGS25 | 7588 | MPH01 | MK302858 | This study |
| | CBGS26 | 7589 | MPH01 | MK302859 | This study |
| | CBGS27 | 7590 | MPH01 | MK302860 | This study |
| | CBGS29 | 7591 | MPH01 | MK302861 | This study |
| | CBGS32 | 7592 | MPH01 | MK302862 | This study |
| | 13-Goesan-01 | | MPH01 | KT382687 | Park et al. (2016) |
| | 13-Goesan-02 | | MPH01 | KT382688 | Park et al. (2016) |
| | 13-Goesan-03 | | MPH01 | KT382693 | Park et al. (2016) |
| | 13-Goesan-04 | | MPH01 | KT382694 | Park et al. (2016) |
| | 13-Goesan-05 | | MPH01 | KT382699 | Park et al. (2016) |
| | 13-Goesan-06 | | MPH01 | KT382700 | Park et al. (2016) |
| 15. Eumsung, CB, Korea (6) | 13-Eumsung-01 | | MPH01 | KT382667 | Park et al. (2016) |
| | 13-Eumsung-02 | | MPH01 | KT382668 | Park et al. (2016) |
| | 13-Eumsung-03 | | MPH01 | KT382673 | Park et al. (2016) |
| | 13-Eumsung-04 | | MPH01 | KT382674 | Park et al. (2016) |
| | 13-Eumsung-05 | | MPH01 | KT382679 | Park et al. (2016) |
| | 13-Eumsung-06 | | MPH01 | KT382680 | Park et al. (2016) |
| 16. Jincheon, CB, Korea (6) | 13-Jincheon-01 | | MPH01 | KT382669 | Park et al. (2016) |
| | 13-Jincheon-02 | | MPH01 | KT382670 | Park et al. (2016) |
| | 13-Jincheon-03 | | MPH01 | KT382675 | Park et al. (2016) |
| | 13-Jincheon-04 | | MPH01 | KT382676 | Park et al. (2016) |
| | 13-Jincheon-05 | | MPH01 | KT382681 | Park et al. (2016) |
| | 13-Jincheon-06 | | MPH01 | KT382682 | Park et al. (2016) |
| 17. Chungju, CB, Korea (6) | 13-Chungju-01 | | MPH01 | KT382683 | Park et al. (2016) |
| | 13-Chungju-02 | | MPH01 | KT382684 | Park et al. (2016) |
| | 13-Chungju-03 | | MPH01 | KT382689 | Park et al. (2016) |
| | 13-Chungju-04 | | MPH01 | KT382690 | Park et al. (2016) |
| | 13-Chungju-05 | | MPH01 | KT382695 | Park et al. (2016) |
| | 13-Chungju-06 | | MPH01 | KT382696 | Park et al. (2016) |

| | | | | |
|---|---|---|---|---|
| 18. Cheongwon, CB, Korea (6) | 13-Cheongwon-01 | | MPH01 | KT382685 | Park et al. (2016) |
| | 13-Cheongwon-02 | | MPH01 | KT382686 | Park et al. (2016) |
| | 13-Cheongwon-03 | | MPH01 | KT382691 | Park et al. (2016) |
| | 13-Cheongwon-04 | | MPH01 | KT382692 | Park et al. (2016) |
| | 13-Cheongwon-05 | | MPH01 | KT382697 | Park et al. (2016) |
| | 13-Cheongwon-06 | | MPH01 | KT382698 | Park et al. (2016) |
| 19. Gongju, CN, Korea (22) | CNGJ01 | 7593 | MPH02 | MK302863 | This study |
| | CNGJ02 | 7594 | MPH02 | MK302864 | This study |
| | CNGJ03 | 7595 | MPH01 | MK302865 | This study |
| | CNGJ04 | 7596 | MPH01 | MK302866 | This study |
| | CNGJ05 | 7597 | MPH01 | MK302867 | This study |
| | CNGJ06 | 7598 | MPH01 | MK302868 | This study |
| | CNGJ09 | 7599 | MPH01 | MK302869 | This study |
| | CNGJ10 | 7600 | MPH01 | MK302870 | This study |
| | CNGJ11 | 7601 | MPH01 | MK302871 | This study |
| | CNGJ12 | 7602 | MPH01 | MK302872 | This study |
| | CNGJ13 | 7603 | MPH01 | MK302873 | This study |
| | CNGJ14 | 7604 | MPH01 | MK302874 | This study |
| | CNGJ15 | 7605 | MPH01 | MK302875 | This study |
| | CNGJ16 | 7606 | MPH01 | MK302876 | This study |
| | CNGJ17 | 7607 | MPH01 | MK302877 | This study |
| | CNGJ18 | 7608 | MPH01 | MK302878 | This study |
| | CNGJ19 | 7609 | MPH01 | MK302879 | This study |
| | CNGJ20 | 7610 | MPH01 | MK302880 | This study |
| | CNGJ21 | 7611 | MPH01 | MK302881 | This study |
| | CNGJ22 | 7612 | MPH01 | MK302882 | This study |
| | CNGJ23 | 7613 | MPH01 | MK302883 | This study |
| | CNGJ24 | 7614 | MPH01 | MK302884 | This study |
| 20. Geumsan, CN, Korea (29) | CNGS01 | 7615 | MPH01 | MK302885 | This study |
| | CNGS04 | 7616 | MPH01 | MK302886 | This study |
| | CNGS07 | 7617 | MPH01 | MK302887 | This study |
| | CNGS08 | 7618 | MPH01 | MK302888 | This study |
| | CNGS09 | 7619 | MPH01 | MK302889 | This study |
| | CNGS10 | 7620 | MPH01 | MK302890 | This study |
| | CNGS11 | 7621 | MPH01 | MK302891 | This study |
| | CNGS12 | 7622 | MPH01 | MK302892 | This study |
| | CNGS13 | 7623 | MPH01 | MK302893 | This study |
| | CNGS14 | 7624 | MPH01 | MK302894 | This study |
| | CNGS15 | 7625 | MPH01 | MK302895 | This study |
| | CNGS16 | 7626 | MPH01 | MK302896 | This study |
| | CNGS17 | 7627 | MPH01 | MK302897 | This study |
| | CNGS19 | 7628 | MPH01 | MK302898 | This study |
| | CNGS20 | 7629 | MPH01 | MK302899 | This study |
| | CNGS22 | 7630 | MPH01 | MK302900 | This study |
| | CNGS23 | 7631 | MPH01 | MK302901 | This study |
| | CNGS24 | 7632 | MPH01 | MK302902 | This study |
| | CNGS25 | 7633 | MPH01 | MK302903 | This study |
| | CNGS27 | 7634 | MPH01 | MK302904 | This study |
| | CNGS28 | 7635 | MPH01 | MK302905 | This study |
| | CNGS29 | 7636 | MPH01 | MK302906 | This study |
| | CNGS30 | 7637 | MPH01 | MK302907 | This study |
| | 13-Geumsan-01 | | MPH01 | KT382557 | Park et al. (2016) |
| | 13-Geumsan-02 | | MPH01 | KT382558 | Park et al. (2016) |
| | 13-Geumsan-03 | | MPH01 | KT382559 | Park et al. (2016) |
| | 13-Geumsan-04 | | MPH01 | KT382560 | Park et al. (2016) |
| | 13-Geumsan-05 | | MPH01 | KT382561 | Park et al. (2016) |
| | 13-Geumsan-06 | | MPH01 | KT382562 | Park et al. (2016) |
| 21. Taean, CN, Korea (32) | CNTA01 | 7638 | MPH01 | MK302908 | This study |
| | CNTA02 | 7639 | MPH01 | MK302909 | This study |
| | CNTA03 | 7640 | MPH01 | MK302910 | This study |
| | CNTA04 | 7641 | MPH01 | MK302911 | This study |
| | CNTA05 | 7642 | MPH01 | MK302912 | This study |
| | CNTA06 | 7643 | MPH01 | MK302913 | This study |
| | CNTA07 | 7644 | MPH01 | MK302914 | This study |
| | CNTA08 | 7645 | MPH01 | MK302915 | This study |
| | CNTA09 | 7646 | MPH01 | MK302916 | This study |
| | CNTA21 | 7647 | MPH01 | MK302917 | This study |
| | CNTA23 | 7648 | MPH01 | MK302918 | This study |
| | CNTA24 | 7649 | MPH01 | MK302919 | This study |
| | CNTA25 | 7650 | MPH01 | MK302920 | This study |
| | CNTA26 | 7651 | MPH01 | MK302921 | This study |
| | CNTA28 | 7652 | MPH01 | MK302922 | This study |
| | CNTA29 | 7653 | MPH01 | MK302923 | This study |

| | | | | | |
|---|---|---|---|---|---|
| | CNTA31 | 7654 | MPH01 | MK302924 | This study |
| | CNTA32 | 7655 | MPH01 | MK302925 | This study |
| | CNTA33 | 7656 | MPH01 | MK302926 | This study |
| | CNTA34 | 7657 | MPH01 | MK302927 | This study |
| | CNTA35 | 7658 | MPH01 | MK302928 | This study |
| | CNTA36 | 7659 | MPH01 | MK302929 | This study |
| | CNTA37 | 7660 | MPH01 | MK302930 | This study |
| | CNTA38 | 7661 | MPH01 | MK302931 | This study |
| | CNTA39 | 7662 | MPH01 | MK302932 | This study |
| | CNTA40 | 7663 | MPH01 | MK302933 | This study |
| | 13-Taean-01 | | MPH01 | KT382569 | Park et al. (2016) |
| | 13-Taean-02 | | MPH01 | KT382570 | Park et al. (2016) |
| | 13-Taean-03 | | MPH01 | KT382571 | Park et al. (2016) |
| | 13-Taean-04 | | MPH01 | KT382572 | Park et al. (2016) |
| | 13-Taean-05 | | MPH01 | KT382573 | Park et al. (2016) |
| | 13-Taean-06 | | MPH01 | KT382574 | Park et al. (2016) |
| 22. Yesan, CN, Korea (23) | CNYS01 | 7664 | MPH01 | MK302934 | This study |
| | CNYS02 | 7665 | MPH01 | MK302935 | This study |
| | CNYS03 | 7666 | MPH01 | MK302936 | This study |
| | CNYS04 | 7667 | MPH01 | MK302937 | This study |
| | CNYS05 | 7668 | MPH01 | MK302938 | This study |
| | CNYS06 | 7669 | MPH01 | MK302939 | This study |
| | CNYS07 | 7670 | MPH01 | MK302940 | This study |
| | CNYS08 | 7671 | MPH01 | MK302941 | This study |
| | CNYS09 | 7672 | MPH01 | MK302942 | This study |
| | CNYS10 | 7673 | MPH01 | MK302943 | This study |
| | CNYS11 | 7674 | MPH01 | MK302944 | This study |
| | CNYS12 | 7675 | MPH01 | MK302945 | This study |
| | CNYS13 | 7676 | MPH01 | MK302946 | This study |
| | CNYS14 | 7677 | MPH01 | MK302947 | This study |
| | CNYS15 | 7678 | MPH01 | MK302948 | This study |
| | CNYS16 | 7679 | MPH01 | MK302949 | This study |
| | CNYS17 | 7680 | MPH01 | MK302950 | This study |
| | CNYS18 | 7681 | MPH01 | MK302951 | This study |
| | CNYS19 | 7682 | MPH01 | MK302952 | This study |
| | CNYS20 | 7683 | MPH01 | MK302953 | This study |
| | CNYS22 | 7684 | MPH01 | MK302954 | This study |
| | CNYS28 | 7685 | MPH01 | MK302955 | This study |
| | CNYS30 | 7686 | MPH01 | MK302956 | This study |
| 23. Cheonan, CN, Korea (6) | 13-Cheonan-01 | | MPH01 | KT382563 | Park et al. (2016) |
| | 13-Cheonan-02 | | MPH01 | KT382564 | Park et al. (2016) |
| | 13-Cheonan-03 | | MPH01 | KT382565 | Park et al. (2016) |
| | 13-Cheonan-04 | | MPH01 | KT382566 | Park et al. (2016) |
| | 13-Cheonan-05 | | MPH01 | KT382567 | Park et al. (2016) |
| | 13-Cheonan-06 | | MPH01 | KT382568 | Park et al. (2016) |
| 24. Daegu, Korea (20) | DG02 | 7687 | MPH01 | MK302957 | This study |
| | DG03 | 7688 | MPH01 | MK302958 | This study |
| | DG04 | 7689 | MPH01 | MK302959 | This study |
| | DG05 | 7690 | MPH01 | MK302960 | This study |
| | DG06 | 7691 | MPH01 | MK302961 | This study |
| | DG07 | 7692 | MPH01 | MK302962 | This study |
| | DG08 | 7693 | MPH01 | MK302963 | This study |
| | DG09 | 7694 | MPH01 | MK302964 | This study |
| | DG11 | 7695 | MPH01 | MK302965 | This study |
| | DG12 | 7696 | MPH01 | MK302966 | This study |
| | DG13 | 7697 | MPH01 | MK302967 | This study |
| | DG14 | 7698 | MPH01 | MK302968 | This study |
| | DG15 | 7699 | MPH01 | MK302969 | This study |
| | DG16 | 7700 | MPH01 | MK302970 | This study |
| | DG17 | 7701 | MPH01 | MK302971 | This study |
| | DG18 | 7702 | MPH01 | MK302972 | This study |
| | DG19 | 7703 | MPH01 | MK302973 | This study |
| | DG20 | 7704 | MPH01 | MK302974 | This study |
| | DG21 | 7705 | MPH01 | MK302975 | This study |
| | DG22 | 7706 | MPH01 | MK302976 | This study |
| 25. Cheongdo, GB, Korea (20) | GBCD01 | 7728 | MPH01 | MK302998 | This study |
| | GBCD03 | 7729 | MPH01 | MK302999 | This study |
| | GBCD04 | 7730 | MPH01 | MK303000 | This study |
| | GBCD05 | 7731 | MPH01 | MK303001 | This study |
| | GBCD06 | 7732 | MPH01 | MK303002 | This study |
| | GBCD07 | 7733 | MPH01 | MK303003 | This study |
| | GBCD08 | 7734 | MPH01 | MK303004 | This study |
| | GBCD09 | 7735 | MPH01 | MK303005 | This study |

| | | | | | |
|---|---|---|---|---|---|
| | GBCD10 | 7736 | MPH01 | MK303006 | This study |
| | GBCD11 | 7737 | MPH01 | MK303007 | This study |
| | GBCD12 | 7738 | MPH01 | MK303008 | This study |
| | GBCD13 | 7739 | MPH01 | MK303009 | This study |
| | GBCD14 | 7740 | MPH01 | MK303010 | This study |
| | GBCD15 | 7741 | MPH01 | MK303011 | This study |
| | GBCD16 | 7742 | MPH01 | MK303012 | This study |
| | GBCD17 | 7743 | MPH01 | MK303013 | This study |
| | GBCD18 | 7744 | MPH01 | MK303014 | This study |
| | GBCD19 | 7745 | MPH01 | MK303015 | This study |
| | GBCD21 | 7746 | MPH01 | MK303016 | This study |
| | GBCD22 | 7747 | MPH01 | MK303017 | This study |
| 26. Chilgok, GB, Korea (33) | GBCGA03 | 7748 | MPH01 | MK303018 | This study |
| | GBCGA04 | 7749 | MPH01 | MK303019 | This study |
| | GBCGA05 | 7750 | MPH01 | MK303020 | This study |
| | GBCGA06 | 7751 | MPH01 | MK303021 | This study |
| | GBCGA07 | 7752 | MPH02 | MK303022 | This study |
| | GBCGA11 | 7753 | MPH01 | MK303023 | This study |
| | GBCGA21 | 7754 | MPH01 | MK303024 | This study |
| | GBCGB01 | 7755 | MPH01 | MK303025 | This study |
| | GBCGB02 | 7756 | MPH01 | MK303026 | This study |
| | GBCGB03 | 7757 | MPH01 | MK303027 | This study |
| | GBCGB04 | 7758 | MPH01 | MK303028 | This study |
| | GBCGB09 | 7759 | MPH01 | MK303029 | This study |
| | GBCGB14 | 7760 | MPH01 | MK303030 | This study |
| | GBCGB15 | 7761 | MPH01 | MK303031 | This study |
| | GBCGB16 | 7762 | MPH01 | MK303032 | This study |
| | GBCGB17 | 7763 | MPH01 | MK303033 | This study |
| | GBCGB18 | 7764 | MPH01 | MK303034 | This study |
| | GBCGB19 | 7765 | MPH01 | MK303035 | This study |
| | GBCGB21 | 7766 | MPH01 | MK303036 | This study |
| | GBCGB22 | 7767 | MPH01 | MK303037 | This study |
| | GBCGB23 | 7768 | MPH01 | MK303038 | This study |
| | GBCGB27 | 7769 | MPH01 | MK303039 | This study |
| | GBCGB30 | 7770 | MPH01 | MK303040 | This study |
| | GBCGB33 | 7771 | MPH01 | MK303041 | This study |
| | GBCGB35 | 7772 | MPH01 | MK303042 | This study |
| | GBCGB36 | 7773 | MPH01 | MK303043 | This study |
| | GBCGB37 | 7774 | MPH01 | MK303044 | This study |
| | 13-Chilgok-01 | | MPH01 | KT382701 | Park et al. (2016) |
| | 13-Chilgok-02 | | MPH01 | KT382702 | Park et al. (2016) |
| | 13-Chilgok-03 | | MPH01 | KT382703 | Park et al. (2016) |
| | 13-Chilgok-04 | | MPH01 | KT382704 | Park et al. (2016) |
| | 13-Chilgok-05 | | MPH01 | KT382705 | Park et al. (2016) |
| | 13-Chilgok-06 | | MPH01 | KT382706 | Park et al. (2016) |
| 27. Sangju, GB, Korea (24) | GBSJ01 | 10619 | MPH01 | MN790205 | This study |
| | GBSJ02 | 10620 | MPH01 | MN790206 | This study |
| | GBSJ03 | 10621 | MPH01 | MN790207 | This study |
| | GBSJ04 | 10622 | MPH01 | MN790208 | This study |
| | GBSJ05 | 10623 | MPH01 | MN790209 | This study |
| | GBSJ06 | 10624 | MPH01 | MN790210 | This study |
| | GBSJ07 | 10625 | MPH01 | MN790211 | This study |
| | GBSJ08 | 10626 | MPH01 | MN790212 | This study |
| | GBSJ09 | 10627 | MPH01 | MN790213 | This study |
| | GBSJ10 | 10628 | MPH01 | MN790214 | This study |
| | GBSJ11 | 10629 | MPH01 | MN790215 | This study |
| | GBSJ12 | 10630 | MPH01 | MN790216 | This study |
| | GBSJ13 | 9900 | MPH01 | MN790217 | This study |
| | GBSJ14 | 9901 | MPH01 | MN790218 | This study |
| | GBSJ15 | 9902 | MPH01 | MN790219 | This study |
| | GBSJ16 | 9903 | MPH01 | MN790220 | This study |
| | GBSJ17 | 9904 | MPH01 | MN790221 | This study |
| | GBSJ18 | 9905 | MPH01 | MN790222 | This study |
| | GBSJ19 | 9906 | MPH01 | MN790223 | This study |
| | GBSJ20 | 9907 | MPH01 | MN790224 | This study |
| | GBSJ21 | 9908 | MPH01 | MN790225 | This study |
| | GBSJ22 | 9909 | MPH01 | MN790226 | This study |
| | GBSJ23 | 9910 | MPH01 | MN790227 | This study |
| | GBSJ24 | 9911 | MPH01 | MN790228 | This study |
| 28. Yeongju, GB, Korea (12) | GBYJ01 | 10607 | MPH01 | MN790229 | This study |
| | GBYJ02 | 10608 | MPH01 | MN790230 | This study |
| | GBYJ03 | 10609 | MPH01 | MN790231 | This study |
| | GBYJ04 | 10610 | MPH01 | MN790232 | This study |
| | GBYJ05 | 10611 | MPH01 | MN790233 | This study |

| | | | | | |
|---|---|---|---|---|---|
| | GBYJ06 | 10612 | MPH02 | MN790234 | This study |
| | GBYJ07 | 10613 | MPH02 | MN790235 | This study |
| | GBYJ08 | 10614 | MPH01 | MN790236 | This study |
| | GBYJ09 | 10615 | MPH01 | MN790237 | This study |
| | GBYJ10 | 10616 | MPH01 | MN790238 | This study |
| | GBYJ11 | 10617 | MPH01 | MN790239 | This study |
| | GBYJ12 | 10618 | MPH01 | MN790240 | This study |
| 29. Gimhae, GN, Korea (30) | GNGHB02 | 7775 | MPH01 | MK303045 | This study |
| | GNGHB03 | 7776 | MPH01 | MK303046 | This study |
| | GNGHB05 | 7777 | MPH01 | MK303047 | This study |
| | GNGHB06 | 7778 | MPH01 | MK303048 | This study |
| | GNGHB07 | 7779 | MPH01 | MK303049 | This study |
| | GNGHB08 | 7780 | MPH01 | MK303050 | This study |
| | GNGHB09 | 7781 | MPH01 | MK303051 | This study |
| | GNGHB10 | 7782 | MPH01 | MK303052 | This study |
| | GNGHB11 | 7783 | MPH02 | MK303053 | This study |
| | GNGHB12 | 7784 | MPH01 | MK303054 | This study |
| | GNGHB13 | 7785 | MPH01 | MK303055 | This study |
| | GNGHB14 | 7786 | MPH01 | MK303056 | This study |
| | GNGHB15 | 7787 | MPH01 | MK303057 | This study |
| | GNGHB16 | 7788 | MPH01 | MK303058 | This study |
| | GNGHB17 | 7789 | MPH01 | MK303059 | This study |
| | GNGHB18 | 7790 | MPH01 | MK303060 | This study |
| | GNGHB19 | 7791 | MPH01 | MK303061 | This study |
| | GNGHB20 | 7792 | MPH01 | MK303062 | This study |
| | KR_GN_36_5 | | MPH01 | KJ412919 | Kwon et al. (2015) |
| | KR_GN_36_6 | | MPH01 | KJ412920 | Kwon et al. (2015) |
| | KR_GN_36_7 | | MPH01 | KJ412921 | Kwon et al. (2015) |
| | KR_GN_36_8 | | MPH01 | KJ412922 | Kwon et al. (2015) |
| | KR_GN_36_9 | | MPH01 | KJ412923 | Kwon et al. (2015) |
| | KR_GN_36_10 | | MPH01 | KJ412924 | Kwon et al. (2015) |
| | 13-Gimhae-01 | | MPH01 | KT382545 | Park et al. (2016) |
| | 13-Gimhae-02 | | MPH01 | KT382546 | Park et al. (2016) |
| | 13-Gimhae-03 | | MPH01 | KT382547 | Park et al. (2016) |
| | 13-Gimhae-04 | | MPH01 | KT382548 | Park et al. (2016) |
| | 13-Gimhae-05 | | MPH01 | KT382549 | Park et al. (2016) |
| | 13-Gimhae-06 | | MPH01 | KT382550 | Park et al. (2016) |
| 30. Miryang, GN, Korea (6) | 13-Milyang-01 | | MPH01 | KT382551 | Park et al. (2016) |
| | 13-Milyang-02 | | MPH01 | KT382552 | Park et al. (2016) |
| | 13-Milyang-03 | | MPH01 | KT382553 | Park et al. (2016) |
| | 13-Milyang-04 | | MPH01 | KT382554 | Park et al. (2016) |
| | 13-Milyang-05 | | MPH01 | KT382555 | Park et al. (2016) |
| | 13-Milyang-06 | | MPH01 | KT382556 | Park et al. (2016) |
| 31. Iksan, JB, Korea (26) | JBYS01 | 7873 | MPH01 | MK303143 | This study |
| | JBYS02 | 7874 | MPH01 | MK303144 | This study |
| | JBYS03 | 7875 | MPH01 | MK303145 | This study |
| | JBYS04 | 7876 | MPH01 | MK303146 | This study |
| | JBYS05 | 7877 | MPH01 | MK303147 | This study |
| | JBYS06 | 7878 | MPH01 | MK303148 | This study |
| | JBYS07 | 7879 | MPH01 | MK303149 | This study |
| | JBYS08 | 7880 | MPH01 | MK303150 | This study |
| | JBYS09 | 7881 | MPH01 | MK303151 | This study |
| | JBYS10 | 7882 | MPH01 | MK303152 | This study |
| | JBYS11 | 7883 | MPH01 | MK303153 | This study |
| | JBYS12 | 7884 | MPH01 | MK303154 | This study |
| | JBYS13 | 7885 | MPH01 | MK303155 | This study |
| | JBYS14 | 7886 | MPH01 | MK303156 | This study |
| | JBYS15 | 7887 | MPH01 | MK303157 | This study |
| | JBYS16 | 7888 | MPH01 | MK303158 | This study |
| | JBYS17 | 7889 | MPH01 | MK303159 | This study |
| | JBYS18 | 7890 | MPH01 | MK303160 | This study |
| | JBYS19 | 7891 | MPH01 | MK303161 | This study |
| | JBYS20 | 7892 | MPH01 | MK303162 | This study |
| | 13-Iksan-01 | | MPH01 | KT382575 | Park et al. (2016) |
| | 13-Iksan-02 | | MPH01 | KT382576 | Park et al. (2016) |
| | 13-Iksan-03 | | MPH01 | KT382577 | Park et al. (2016) |
| | 13-Iksan-04 | | MPH01 | KT382578 | Park et al. (2016) |
| | 13-Iksan-05 | | MPH01 | KT382579 | Park et al. (2016) |
| | 13-Iksan-06 | | MPH01 | KT382580 | Park et al. (2016) |
| 32. Gokseong, JN, Korea (25) | JNGS01 | 7893 | MPH01 | MK303163 | This study |
| | JNGS02 | 7894 | MPH01 | MK303164 | This study |
| | JNGS03 | 7895 | MPH01 | MK303165 | This study |
| | JNGS04 | 7896 | MPH01 | MK303166 | This study |

| | | | | |
|---|---|---|---|---|
| | JNGS05 | 7897 | MPH01 | MK303167 | This study |
| | JNGS06 | 7898 | MPH01 | MK303168 | This study |
| | JNGS07 | 7899 | MPH01 | MK303169 | This study |
| | JNGS08 | 7900 | MPH01 | MK303170 | This study |
| | JNGS10 | 7901 | MPH01 | MK303171 | This study |
| | JNGS11 | 7902 | MPH01 | MK303172 | This study |
| | JNGS12 | 7903 | MPH01 | MK303173 | This study |
| | JNGS13 | 7904 | MPH01 | MK303174 | This study |
| | JNGS14 | 7905 | MPH01 | MK303175 | This study |
| | JNGS15 | 7906 | MPH01 | MK303176 | This study |
| | JNGS16 | 7907 | MPH01 | MK303177 | This study |
| | JNGS18 | 7908 | MPH01 | MK303178 | This study |
| | JNGS19 | 7909 | MPH01 | MK303179 | This study |
| | JNGS20 | 7910 | MPH01 | MK303180 | This study |
| | JNGS21 | 7911 | MPH01 | MK303181 | This study |
| | JNGS23 | 7912 | MPH01 | MK303182 | This study |
| | JNGS24 | 7913 | MPH01 | MK303183 | This study |
| | JNGS32 | 7914 | MPH01 | MK303184 | This study |
| | JNGS33 | 7915 | MPH01 | MK303185 | This study |
| | JNGS34 | 7916 | MPH01 | MK303186 | This study |
| | JNGS35 | 7917 | MPH01 | MK303187 | This study |
| 33. Haenam, JN, Korea (20) | JNHN01 | 7918 | MPH01 | MK303188 | This study |
| | JNHN02 | 7919 | MPH01 | MK303189 | This study |
| | JNHN03 | 7920 | MPH01 | MK303190 | This study |
| | JNHN04 | 7921 | MPH01 | MK303191 | This study |
| | JNHN05 | 7922 | MPH02 | MK303192 | This study |
| | JNHN06 | 7923 | MPH01 | MK303193 | This study |
| | JNHN07 | 7924 | MPH01 | MK303194 | This study |
| | JNHN08 | 7925 | MPH01 | MK303195 | This study |
| | JNHN09 | 7926 | MPH01 | MK303196 | This study |
| | JNHN10 | 7927 | MPH01 | MK303197 | This study |
| | JNHN11 | 7928 | MPH01 | MK303198 | This study |
| | JNHN12 | 7929 | MPH01 | MK303199 | This study |
| | JNHN13 | 7930 | MPH01 | MK303200 | This study |
| | JNHN14 | 7931 | MPH01 | MK303201 | This study |
| | JNHN15 | 7932 | MPH02 | MK303202 | This study |
| | JNHN16 | 7933 | MPH01 | MK303203 | This study |
| | JNHN17 | 7934 | MPH01 | MK303204 | This study |
| | JNHN18 | 7935 | MPH01 | MK303205 | This study |
| | JNHN19 | 7936 | MPH01 | MK303206 | This study |
| | JNHN20 | 7937 | MPH01 | MK303207 | This study |
| 34. Jangseong, JN, Korea (20) | JNJS01 | 7938 | MPH01 | MK303208 | This study |
| | JNJS02 | 7939 | MPH01 | MK303209 | This study |
| | JNJS03 | 7940 | MPH01 | MK303210 | This study |
| | JNJS04 | 7941 | MPH01 | MK303211 | This study |
| | JNJS05 | 7942 | MPH01 | MK303212 | This study |
| | JNJS06 | 7943 | MPH01 | MK303213 | This study |
| | JNJS07 | 7944 | MPH01 | MK303214 | This study |
| | JNJS08 | 7945 | MPH01 | MK303215 | This study |
| | JNJS09 | 7946 | MPH01 | MK303216 | This study |
| | JNJS10 | 7947 | MPH01 | MK303217 | This study |
| | JNJS11 | 7948 | MPH01 | MK303218 | This study |
| | JNJS12 | 7949 | MPH01 | MK303219 | This study |
| | JNJS13 | 7950 | MPH01 | MK303220 | This study |
| | JNJS14 | 7951 | MPH01 | MK303221 | This study |
| | JNJS15 | 7952 | MPH01 | MK303222 | This study |
| | JNJS16 | 7953 | MPH01 | MK303223 | This study |
| | JNJS17 | 7954 | MPH01 | MK303224 | This study |
| | JNJS18 | 7955 | MPH01 | MK303225 | This study |
| | JNJS19 | 7956 | MPH01 | MK303226 | This study |
| | JNJS20 | 7957 | MPH01 | MK303227 | This study |
| 35. Suncheon, JN, Korea (20) | JNSC01 | 7958 | MPH01 | MK303228 | This study |
| | JNSC02 | 7959 | MPH02 | MK303229 | This study |
| | JNSC03 | 7960 | MPH01 | MK303230 | This study |
| | JNSC04 | 7961 | MPH01 | MK303231 | This study |
| | JNSC05 | 7962 | MPH01 | MK303232 | This study |
| | JNSC06 | 7963 | MPH01 | MK303233 | This study |
| | JNSC07 | 7964 | MPH01 | MK303234 | This study |
| | JNSC08 | 7965 | MPH01 | MK303235 | This study |
| | JNSC09 | 7966 | MPH02 | MK303236 | This study |
| | JNSC10 | 7967 | MPH01 | MK303237 | This study |
| | JNSC11 | 7968 | MPH01 | MK303238 | This study |

| | | | | | |
|---|---|---|---|---|---|
| | JNSC12 | 7969 | MPH01 | MK303239 | This study |
| | JNSC13 | 7970 | MPH01 | MK303240 | This study |
| | JNSC14 | 7971 | MPH01 | MK303241 | This study |
| | JNSC15 | 7972 | MPH01 | MK303242 | This study |
| | JNSC16 | 7973 | MPH01 | MK303243 | This study |
| | JNSC17 | 7974 | MPH01 | MK303244 | This study |
| | JNSC18 | 7975 | MPH01 | MK303245 | This study |
| | JNSC19 | 7976 | MPH01 | MK303246 | This study |
| | JNSC20 | 7977 | MPH01 | MK303247 | This study |
| 36. Maryland, USA (4) | USA_MD_60_6 | | MPH04 | KJ412927 | Kwon et al. (2015), Park et al. (2016) |
| | USA_MD_60_8 | | MPH05 | KJ412928 | Kwon et al. (2015), Park et al. (2016) |
| | USA_MD_60_9 | | MPH05 | KJ412929 | Kwon et al. (2015), Park et al. (2016) |
| | USA_MD_60_10 | | MPH06 | KJ412930 | Kwon et al. (2015), Park et al. (2016) |
| 37. New Jersey, USA (10) | USA_NJ_94_1 | | MPH07 | KJ412931 | Kwon et al. (2015), Park et al. (2016) |
| | USA_NJ_94_2 | | MPH08 | KJ412932 | Kwon et al. (2015), Park et al. (2016) |
| | USA_NJ_94_3 | | MPH09 | KJ412933 | Kwon et al. (2015), Park et al. (2016) |
| | USA_NJ_94_4 | | MPH08 | KJ412934 | Kwon et al. (2015), Park et al. (2016) |
| | USA_NJ_94_5 | | MPH10 | KJ412935 | Kwon et al. (2015), Park et al. (2016) |
| | USA_NJ_94_6 | | MPH07 | KJ412936 | Kwon et al. (2015), Park et al. (2016) |
| | USA_NJ_94_7 | | MPH10 | KJ412937 | Kwon et al. (2015), Park et al. (2016) |
| | USA_NJ_94_8 | | MPH11 | KJ412938 | Kwon et al. (2015), Park et al. (2016) |
| | USA_NJ_94_9 | | MPH08 | KJ412939 | Kwon et al. (2015), Park et al. (2016) |
| | USA_NJ_94_10 | | MPH08 | KJ412940 | Kwon et al. (2015), Park et al. (2016) |
| 38. West virginia, USA (22) | 14-WV-01 | | MPH01 | KT382707 | Park et al. (2016) |
| | 14-WV-02 | | MPH15 | KT382708 | Park et al. (2016) |
| | 14-WV-03 | | MPH14 | KT382709 | Park et al. (2016) |
| | 14-WV-04 | | MPH02 | KT382710 | Park et al. (2016) |
| | 14-WV-05 | | MPH15 | KT382711 | Park et al. (2016) |
| | 14-WV-06 | | MPH13 | KT382712 | Park et al. (2016) |
| | 14-WV-07 | | MPH02 | KT382713 | Park et al. (2016) |
| | 14-WV-08 | | MPH15 | KT382714 | Park et al. (2016) |
| | 14-WV-09 | | MPH16 | KT382715 | Park et al. (2016) |
| | 14-WV-10 | | MPH17 | KT382716 | Park et al. (2016) |
| | 14-WV-11 | | MPH17 | KT382717 | Park et al. (2016) |
| | 14-WV-12 | | MPH10 | KT382718 | Park et al. (2016) |
| | 14-WV-13 | | MPH10 | KT382719 | Park et al. (2016) |
| | 14-WV-14 | | MPH16 | KT382720 | Park et al. (2016) |
| | 14-WV-15 | | MPH17 | KT382721 | Park et al. (2016) |
| | 14-WV-16 | | MPH16 | KT382722 | Park et al. (2016) |
| | 14-WV-17 | | MPH15 | KT382723 | Park et al. (2016) |
| | 14-WV-18 | | MPH02 | KT382724 | Park et al. (2016) |
| | 14-WV-19 | | MPH14 | KT382725 | Park et al. (2016) |
| | 14-WV-20 | | MPH02 | KT382726 | Park et al. (2016) |
| | 14-WV-21 | | MPH15 | KT382727 | Park et al. (2016) |
| | 14-WV-22 | | MPH03 | KT382728 | Park et al. (2016) |
| 39. Ontario, Canada (58) | BIOUG02381-E12 | | MPH10 | KJ444676 | Unpublished, |
| | BIOUG02381-F06 | | MPH10 | KJ209286 | Unpublished, |
| | BIOUG03516-H11 | | MPH10 | KJ167570 | Unpublished, |
| | BIOUG02381-E04 | | MPH10 | KJ164858 | Unpublished, |
| | BIOUG03516-G07 | | MPH10 | KJ092562 | Unpublished, |
| | BIOUG06799-G01 | | MPH10 | KR030519 | Gwiazdowski et al. (2015) |
| | HLC-17466 | | MPH10 | KR030936 | Gwiazdowski et al. (2015) |
| | HLC-17469 | | MPH10 | KR031026 | Gwiazdowski et al. (2015) |
| | BIOUG01019-D09 | | MPH10 | KR032036 | Gwiazdowski et al. (2015) |
| | BIOUG02749-E04 | | MPH10 | KR033160 | Gwiazdowski et al. (2015) |
| | HLC-17482 | | MPH10 | KR033498 | Gwiazdowski et al. (2015) |
| | BIOUG02749-C05 | | MPH10 | KR033899 | Gwiazdowski et al. (2015) |
| | BIOUG01019-C10 | | MPH10 | KR034098 | Gwiazdowski et al. (2015) |
| | BIOUG02749-C03 | | MPH10 | KR034489 | Gwiazdowski et al. (2015) |

| | | | | | |
|---|---|---|---|---|---|
| | BIOUG02749-E06 | | MPH10 | KR034917 | Gwiazdowski et al. (2015) |
| | BIOUG00856-C04 | | MPH10 | KR034927 | Gwiazdowski et al. (2015) |
| | BIOUG00856-C05 | | MPH10 | KR035057 | Gwiazdowski et al. (2015) |
| | BIOUG02749-E05 | | MPH10 | KR035119 | Gwiazdowski et al. (2015) |
| | BIOUG06799-H02 | | MPH10 | KR035123 | Gwiazdowski et al. (2015) |
| | HLC-17467 | | MPH10 | KR035475 | Gwiazdowski et al. (2015) |
| | HLC-17451 | | MPH10 | KR035890 | Gwiazdowski et al. (2015) |
| | BIOUG03716-G05 | | MPH10 | KR036478 | Gwiazdowski et al. (2015) |
| | BIOUG03716-H10 | | MPH10 | KR037454 | Gwiazdowski et al. (2015) |
| | HLC-17479 | | MPH10 | KR037535 | Gwiazdowski et al. (2015) |
| | BIOUG03852-A08 | | MPH10 | KR037966 | Gwiazdowski et al. (2015) |
| | BIOUG01019-C12 | | MPH10 | KR038049 | Gwiazdowski et al. (2015) |
| | HLC-17468 | | MPH10 | KR039262 | Gwiazdowski et al. (2015) |
| | BIOUG02749-B02 | | MPH10 | KR039315 | Gwiazdowski et al. (2015) |
| | BIOUG02749-E02 | | MPH10 | KR039771 | Gwiazdowski et al. (2015) |
| | BIOUG01019-E03 | | MPH10 | KR039955 | Gwiazdowski et al. (2015) |
| | BIOUG01807-D07 | | MPH10 | KR040575 | Gwiazdowski et al. (2015) |
| | HLC-17447 | | MPH10 | KR043317 | Gwiazdowski et al. (2015) |
| | BIOUG01019-C11 | | MPH18 | KR043727 | Gwiazdowski et al. (2015) |
| | BIOUG00856-F09 | | MPH10 | KR044426 | Gwiazdowski et al. (2015) |
| | BIOUG01594-E07 | | MPH10 | KR045216 | Gwiazdowski et al. (2015) |
| | BIOUG09125-G04 | | MPH10 | KR344413 | Gwiazdowski et al. (2015) |
| | BIOUG11729-B01 | | MPH10 | KR345234 | Unpublished |
| | BIOUG09125-C12 | | MPH19 | KR345765 | Unpublished |
| | BIOUG09125-F12 | | MPH10 | KR345776 | Unpublished |
| | BIOUG10062-B05 | | MPH10 | KR346314 | Unpublished |
| | BIOUG09125-C11 | | MPH10 | KR346668 | Unpublished |
| | BIOUG10062-A01 | | MPH10 | KR346749 | Unpublished |
| | BIOUG09125-D09 | | MPH19 | KR342124 | Unpublished |
| | BIOUG10062-A08 | | MPH10 | KR343104 | Unpublished |
| | BIOUG09125-D10 | | MPH10 | KR343336 | Unpublished |
| | BIOUG08453-H03 | | MPH10 | KR343513 | Unpublished |
| | BIOUG09364-H03 | | MPH10 | KR343732 | Unpublished |
| | BIOUG08686-D06 | | MPH10 | KR563162 | Hebert et al. (2016) |
| | BIOUG10861-E05 | | MPH10 | KR583538 | Hebert et al. (2016) |
| | BIOUG10778-E06 | | MPH10 | KR584387 | Hebert et al. (2016) |
| | HEMI 0104.02 | | MPH10 | KR574709 | Hebert et al. (2016) |
| | BIOUG31067-F10 | | MPH10 | MG397590 | Unpublished |
| | BIOUG31061-C09 | | MPH10 | MG400707 | Unpublished |
| | BIOUG31030-G09 | | MPH10 | MG405117 | Unpublished |
| | BIOUG27096-E02 | | MPH20 | MF932798 | Unpublished |
| | BIOUG20301-H07 | | MPH10 | MF929818 | Unpublished |
| | BIOUG27096-E07 | | MPH10 | MF937118 | Unpublished |
| | BIOUG26203-H09 | | MPH10 | MF938242 | Unpublished |
| 40. Saskatchewan, Canada (6) | BIOUG02749-F06 | | MPH10 | KR030677 | Gwiazdowski et al. (2015) |
| | BIOUG02749-F04 | | MPH10 | KR033782 | Gwiazdowski et al. (2015) |
| | BIOUG02749-E08 | | MPH10 | KR036440 | Gwiazdowski et al. (2015) |
| | BIOUG02749-F03 | | MPH10 | KR037211 | Gwiazdowski et al. (2015) |
| | BIOUG02749-F05 | | MPH10 | KR038441 | Gwiazdowski et al. (2015) |
| | BIOUG02749-E10 | | MPH10 | KR039828 | Gwiazdowski et al. (2015) |
| 41. San Remo, Italy (7) | ITA02 | 8002 | MPH01 | MK303272 | This study |
| | ITA03 | 8003 | MPH02 | MK303273 | This study |
| | ITA07 | 8004 | MPH01 | MK303274 | This study |
| | ITA08 | 8005 | MPH02 | MK303275 | This study |
| | IT_SR_87_1 | | MPH01 | KJ412962 | Kwon et al. (2015), Park et al. (2016) |
| | IT_SR_87_2 | | MPH01 | KJ412963 | Kwon et al. (2015), Park et al. (2016) |
| | IT_SR_87_4 | | MPH01 | KJ412965 | Kwon et al. (2015), Park et al. (2016) |
| 42. Savona, Italy (7) | ITA10 | 8006 | MPH01 | MK303276 | This study |
| | ITA11 | 8007 | MPH01 | MK303277 | This study |
| | ITA12 | 8008 | MPH02 | MK303278 | This study |
| | IT_Sv_89_1 | | MPH01 | KJ412966 | Kwon et al. (2015), Park et al. (2016) |
| | IT_Sv_89_2 | | MPH01 | KJ412967 | Kwon et al. (2015), Park et al. (2016) |
| | IT_Sv_89_3 | | MPH01 | KJ412968 | Kwon et al. (2015), Park et al. (2016) |
| | IT_Sv_89_4 | | MPH01 | KJ412969 | Kwon et al. (2015), Park et al. (2016) |
| 43. Borghetto, Italy (13) | ITA13 | 8009 | MPH01 | MK303279 | This study |
| | ITA14 | 8010 | MPH01 | MK303280 | This study |
| | ITA15 | 8011 | MPH01 | MK303281 | This study |

| | | | | | |
|---|---|---|---|---|---|
| | ITA16 | 8012 | MPH01 | MK303282 | This study |
| | ITA17 | 8013 | MPH01 | MK303283 | This study |
| | ITA18 | 8014 | MPH01 | MK303284 | This study |
| | ITA19 | 8015 | MPH02 | MK303285 | This study |
| | ITA20 | 8016 | MPH01 | MK303286 | This study |
| | ITA21 | 8017 | MPH02 | MK303287 | This study |
| | ITA22 | 8018 | MPH02 | MK303288 | This study |
| | IT_Bg_90_1 | | MPH01 | KJ412970 | Kwon et al. (2015), Park et al. (2016) |
| | IT_Bg_90_2 | | MPH01 | KJ412971 | Kwon et al. (2015), Park et al. (2016) |
| | IT_Bg_90_3 | | MPH01 | KJ412972 | Kwon et al. (2015), Park et al. (2016) |
| 44. Genova, Italy (5) | ITA23 | 8019 | MPH01 | MK303289 | This study |
| | ITA24 | 8020 | MPH01 | MK303290 | This study |
| | ITA25 | 8021 | MPH01 | MK303291 | This study |
| | ITA26 | 8022 | MPH01 | MK303292 | This study |
| | ITA27 | 8023 | MPH02 | MK303293 | This study |
| 45. Lleida, Spain (14) | SPA02 | 8024 | MPH01 | MK303294 | This study |
| | SPA04 | 8025 | MPH01 | MK303295 | This study |
| | SPA06 | 8026 | MPH01 | MK303296 | This study |
| | SPA07 | 8027 | MPH01 | MK303297 | This study |
| | SPA08 | 8028 | MPH01 | MK303298 | This study |
| | SPA10 | 8029 | MPH01 | MK303299 | This study |
| | SPA13 | 8030 | MPH01 | MK303300 | This study |
| | SPA14 | 8031 | MPH01 | MK303301 | This study |
| | SPA15 | 8032 | MPH01 | MK303302 | This study |
| | ES_LM_64_7 | | MPH01 | KJ412956 | Kwon et al. (2015), Park et al. (2016) |
| | ES_LM_64_8 | | MPH01 | KJ412957 | Kwon et al. (2015), Park et al. (2016) |
| | ES_LM_64_10 | | MPH01 | KJ412959 | Kwon et al. (2015), Park et al. (2016) |
| | ES_LM_64_11 | | MPH01 | KJ412960 | Kwon et al. (2015), Park et al. (2016) |
| | ES_LM_64_12 | | MPH01 | KJ412961 | Kwon et al. (2015), Park et al. (2016) |
| 46. INRA, France (1) | FRA21 (INRA_6_5) | 8033 | MPH01 | MK303303 | This study |
| 47. Montpellie, France (12) | FRA27 | 8034 | MPH02 | MK303304 | This study |
| | FRA29 | 8036 | MPH02 | MK303306 | This study |
| | FRA30 | 8037 | MPH02 | MK303307 | This study |
| | FR_Mp_83_1 | | MPH01 | KJ412941 | Kwon et al. (2015), Park et al. (2016) |
| | FR_Mp_83_3 | | MPH01 | KJ412943 | Kwon et al. (2015), Park et al. (2016) |
| | FR_Mp_83_4 | | MPH02 | KJ412944 | Kwon et al. (2015), Park et al. (2016) |
| | FRA31 | 8038 | MPH02 | MK303308 | This study |
| | FRA32 | 8039 | MPH02 | MK303309 | This study |
| | FRA33 | 8040 | MPH01 | MK303310 | This study |
| | FRA34 | 8041 | MPH01 | MK303311 | This study |
| | FRA36 | 8042 | MPH01 | MK303312 | This study |
| | FRA37 | 8043 | MPH02 | MK303313 | This study |
| 48. ARS, France (4) | FRA38 | 8044 | MPH01 | MK303314 | This study |
| | FRA39 | 8045 | MPH01 | MK303315 | This study |
| | FRA40 | 8046 | MPH02 | MK303316 | This study |
| | FRA41 | 8047 | MPH01 | MK303317 | This study |
| 49. Le Boulou, France (4) | FR_LB_93_1 | | MPH12 | KJ412945 | Kwon et al. (2015), Park et al. (2016) |
| | FR_LB_93_2 | | MPH12 | KJ412946 | Kwon et al. (2015), Park et al. (2016) |
| | FR_LB_93_3 | | MPH12 | KJ412947 | Kwon et al. (2015), Park et al. (2016) |
| | FR_LB_93_4 | | MPH01 | KJ412948 | Kwon et al. (2015), Park et al. (2016) |
| 50. Pri Hrastu, Slovenia (4) | SI_Ph_66_6 | | MPH01 | KJ412952 | Kwon et al. (2015), Park et al. (2016) |
| | SI_Ph_66_7 | | MPH01 | KJ412953 | Kwon et al. (2015), Park et al. (2016) |
| | SI_Ph_66_10 | | MPH02 | KJ412954 | Kwon et al. (2015), Park et al. (2016) |
| | SI_Ph_66_11 | | MPH02 | KJ412955 | Kwon et al. (2015), Park et al. (2016) |

GG, Gyeonggi-do Province; GW, Gangwon-do Province; CB, Chungcheongbuk-do Province; CN, Chungcheongnam-do Province; GB, Gyeongsangbuk-do Province; GN, Gyeongsangnam-do Province; JB, Jeollabuk-do Province; and JN, Jeollanam-do Province. INRA, The French National Institute for Agricultural Research; and ARS, Ars-sur-Formans

**Table S4.** Relative frequencies of the DNA barcoding region (658 bp)-based haplotypes for 536 individuals of *Metcalfa pruinosa* collected in Korea and three European countries

| Country | Locality | Haplotype | | |
|---|---|---|---|---|
| | | MPBAR01 | MPBAR02 | MPBAR03 |
| | Total (536) | 0.922 (494) | 0.076 (41) | 0.002 (1) |
| Korea | 1. Seoul, Korea (23) | 0.917 (22) | 0.083 (1) | |
| | 2. Incheon, Korea (20) | 1.000 (20) | | |
| | 3. Anseong, Korea (21) | 0.905 (19) | 0.095 (2) | |
| | 4. Yeoju, Korea (22) | 0.591 (13) | 0.364 (8) | 0.045 (1) |
| | 5. Pyeongchang, Korea (20) | 0.800 (16) | 0.200 (4) | |
| | 6. Wonju, Korea (20) | 1.000 (20) | | |
| | 7. Danyang, Korea (23) | 0.957 (22) | 0.043 (1) | |
| | 8. Goesan, Korea (21) | 1.000 (21) | | |
| | 9. Gongju, Korea (22) | 0.909 (20) | 0.091 (2) | |
| | 10. Geumsan, Korea (23) | 1.000 (23) | | |
| | 11. Taean, Korea (26) | 1.000 (26) | | |
| | 12. Yesan, Korea (23) | 1.000 (23) | | |
| | 13. Daegu, Korea (20) | 1.000 (20) | | |
| | 14. Cheongdo, Korea (20) | 1.000 (20) | | |
| | 15. Chilgok, Korea (27) | 0.963 (26) | 0.037 (1) | |
| | 16. Sangju, Korea (24) | 1.000 (24) | | |
| | 17. Yeongju, Korea (12) | 0.833 (10) | 0.167 (2) | |
| | 18. Gimhae, Korea (18) | 0.944 (17) | 0.056 (1) | |
| | 19. Iksan, Korea (20) | 1.000 (20) | | |
| | 20. Gokseong, Korea (25) | 1.000 (25) | | |
| | 21. Haenam, Korea (20) | 0.900 (18) | 0.100 (2) | |
| | 22. Jangseong, Korea (20) | 1.000 (20) | | |
| | 23. Suncheon, Korea (20) | 0.900 (18) | 0.100 (2) | |
| | Total (491) | 0.943 (463) | 0.055 (27) | 0.002 (1) |
| Italy | 24. San Remo, Italy (4) | 0.500 (2) | 0.500 (2) | |
| | 25. Savona, Italy (3) | 0.667 (2) | 0.333 (1) | |
| | 26. Borghetto, Italy (10) | 0.700 (7) | 0.300 (3) | |
| | 27. Genova, Italy (5) | 0.800 (4) | 0.200 (1) | |
| | Total (22) | 0.682 (15) | 0.318 (7) | |
| Spain | 28. Lleida, Spain (9) | 1.000 (9) | | |
| | Total (9) | 1.000 (9) | | |
| France | 29. INRA, France (1) | 1.000 (1) | | |
| | 30. Montpellie, France (9) | 0.333 (3) | 0.667 (6) | |
| | 31. ARS, France (4) | 0.750 (3) | 0.250 (1) | |
| | Total (14) | 0.500 (7) | 0.500 (7) | |

Numbers in parentheses indicate number of individuals

**Table S5.** Within-country diversity estimates of *Metcalfa pruinosa* based on worldwide *COI* sequences

| Locality | SS[a] | NH[b] | *H*[c] | NP[d] | MSD[e] (%) | MPD[f] | π[g] |
|---|---|---|---|---|---|---|---|
| 1. Korea | 659 | 3 | 0.1068 ± 0.0161 | 3 | 0.64 | 0.224380 | 0.000477 ± 0.000625 |
| 2. USA | 36 | 16 | 0.9413 ± 0.0166 | 39 | 2.13 | 9.585714 | 0.020395 ± 0.010641 |
| 3. Canada | 64 | 4 | 0.1215 ± 0.0554 | 5 | 0.43 | 0.274306 | 0.000584 ± 0.000711 |
| 4. Italy | 32 | 2 | 0.3528 ± 0.0836 | 2 | 0.43 | 0.705645 | 0.001501 ± 0.001292 |
| 5. Spain | 14 | 1 | - | - | - | - | - |
| 6. France | 21 | 3 | 0.6381 ± 0.0580 | 8 | 2.13 | 2.590476 | 0.005512 ± 0.003431 |
| 7. Slovenia | 4 | 2 | 0.6667 ± 0.2041 | 2 | 0.43 | 1.333333 | 0.002837 ± 0.002604 |

[a]Sample size
[b]Number of haplotypes
[c]Haplotype diversity with standard error
[d]Number of polymorphic sites
[e]Maximum sequence divergence
[f]Mean number of pairwise differences
[g]Nucleotide diversity with standard error
-, not available owing to a single haplotype

**Table S6.** Pairwise comparisons among 15 Region 5 haplotypes of *Metcalfa pruinosa*

| Haplotype | 1 | 2 | 3 | 4 | 5 | 6 | 7 | 8 | 9 | 10 | 11 | 12 | 13 | 14 | 15 |
|---|---|---|---|---|---|---|---|---|---|---|---|---|---|---|---|
| 1. MPR501 | - | 0.47 | 1.64 | 0.47 | 0.23 | 1.17 | 0.70 | 0.23 | 0.94 | 1.40 | 0.23 | 0.23 | 0.47 | 0.47 | 1.87 |
| 2. MPR502 | 2 | - | 1.17 | 0.94 | 0.70 | 0.70 | 0.23 | 0.70 | 0.47 | 0.94 | 0.23 | 0.70 | 0.94 | 0.94 | 1.40 |
| 3. MPR503 | 7 | 5 | - | 1.17 | 1.87 | 0.47 | 1.40 | 1.87 | 0.70 | 0.23 | 1.40 | 1.87 | 2.10 | 2.10 | 0.23 |
| 4. MPR504 | 2 | 4 | 5 | - | 0.70 | 1.64 | 1.17 | 0.70 | 0.47 | 0.94 | 0.70 | 0.70 | 0.94 | 0.94 | 1.40 |
| 5. MPR505 | 1 | 3 | 8 | 3 | - | 1.40 | 0.47 | 0.47 | 1.17 | 1.64 | 0.47 | 0.47 | 0.70 | 0.70 | 2.10 |
| 6. MPR506 | 5 | 3 | 2 | 7 | 6 | - | 0.94 | 1.40 | 1.17 | 0.70 | 0.94 | 1.40 | 1.64 | 1.64 | 0.70 |
| 7. MPR507 | 3 | 1 | 6 | 5 | 2 | 4 | - | 0.94 | 0.70 | 1.17 | 0.47 | 0.94 | 1.17 | 1.17 | 1.64 |
| 8. MPR508 | 1 | 3 | 8 | 3 | 2 | 6 | 4 | - | 1.17 | 1.64 | 0.47 | 0.47 | 0.70 | 0.70 | 2.10 |
| 9. MPR509 | 4 | 2 | 3 | 2 | 5 | 5 | 3 | 5 | - | 0.47 | 0.70 | 1.17 | 1.40 | 1.40 | 0.94 |
| 10. MPR510 | 6 | 4 | 1 | 4 | 7 | 3 | 5 | 7 | 2 | - | 1.17 | 1.64 | 1.87 | 1.87 | 0.47 |
| 11. MPR511 | 1 | 1 | 6 | 3 | 2 | 4 | 2 | 2 | 3 | 5 | - | 0.47 | 0.70 | 0.70 | 1.64 |
| 12. MPR512 | 1 | 3 | 8 | 3 | 2 | 6 | 4 | 2 | 5 | 7 | 2 | - | 0.70 | 0.70 | 2.10 |
| 13. MPR513 | 2 | 4 | 9 | 4 | 3 | 7 | 5 | 3 | 6 | 8 | 3 | 3 | - | 0.94 | 2.34 |
| 14. MPR514 | 2 | 4 | 9 | 4 | 3 | 7 | 5 | 3 | 6 | 8 | 3 | 3 | 4 | - | 2.34 |
| 15. MPR515 | 8 | 6 | 1 | 6 | 9 | 3 | 7 | 9 | 4 | 2 | 7 | 9 | 10 | 10 | - |

Numbers above the diagonal are percent distance values; numbers below the diagonal are absolute distance values

**Table S7.** Relative frequencies of Region 5 haplotypes for 342 individuals of *Metcalfa pruinosa* collected in Korea and three European countries

| Haplotype | Locality | | | | |
|---|---|---|---|---|---|
| | Korea (297) | Italy (22) | Spain (9) | France (14) | Total |
| 1. MPR501 | 0.515 (153) | 0.318 (7) | 0.333 (3) | 0.286 (4) | 0.488 (167) |
| 2. MPR502 | 0.327 (97) | 0.364 (8) | 0.556 (5) | 0.071 (1) | 0.325 (111) |
| 3. MPR503 | 0.037 (11) | 0.318 (7) | | 0.429 (6) | 0.070 (24) |
| 4. MPR504 | 0.024 (7) | | | | 0.020 (7) |
| 5. MPR505 | 0.007 (2) | | | | 0.006 (2) |
| 6. MPR506 | 0.017 (5) | | | | 0.015 (5) |
| 7. MPR507 | 0.003 (1) | | | | 0.003 (1) |
| 8. MPR508 | 0.047 (14) | | | 0.071 (1) | 0.044 (15) |
| 9. MPR509 | 0.010 (3) | | | | 0.009 (3) |
| 10. MPR510 | 0.007 (2) | | | | 0.006 (2) |
| 11. MPR511 | 0.003 (1) | | | | 0.003 (1) |
| 12. MPR512 | 0.003 (1) | | | | 0.003 (1) |
| 13. MPR513 | | | | 0.071 (1) | 0.003 (1) |
| 14. MPR514 | | | 0.111 (1) | | 0.003 (1) |
| 15. MPR515 | | | | 0.071 (1) | 0.003 (1) |
| Total | 0.868 (297) | 0.064 (22) | 0.026 (9) | 0.040 (14) | 1 (342) |

Numbers in parentheses indicate sample size of each population

**Table S8.** Comparison of three types of marker regions

| Region | Length (bp) | Variable position | Sequence divergence | No. haplotype | No. populations with mono haplotype | No. populations with ≥ 2 haplotypes | Haplotype diversity |
|---|---|---|---|---|---|---|---|
| DNA barcode | 658 | 3 | 0.15-0.46 | 3 | 11 | 13 | 0.1450 ± 0.0197 |
| Region 5 | 424-428 | 14 | 0.23-2.34 | 15 | 4 | 20 | 0.6504 ± 0.0180 |
| DNA barcode + Region 5 | 1,082-1,086 | 17 | 0.09-1.11 | 20 | 4 | 20 | 0.6732 ± 0.0191 |

**Table S9.** Variable positions in the concatenated sequences of the DNA barcoding region and Region 5 in *Metcalfa pruinosa*

| Region | Variable site | Type | Amino acid |
|---|---|---|---|
| DNA barcode | 343 | C ↔ T | Proline |
| | 442 | T ↔ C | Asparagine |
| | 520 | G ↔ A | Leucine |
| Region 5 | 36 | A ↔ C | - |
| | 42 | C ↔ T | - |
| | 68 | C ↔ T | - |
| | 78 | A ↔ T | - |
| | 198 | A ↔ G | - |
| | 228 | C ↔ T | - |
| | 235 | C ↔ T | - |
| | 249-250 | TA ↔ deletions | - |
| | 251 | T ↔ deletion | - |
| | 280 | A ↔ C | - |
| | 289 | C ↔ T | - |
| | 295 | A ↔ G | - |
| | 299-300 | AT ↔ deletions | - |
| | 417 | A ↔ T | - |

*Nucleotide position is with regard to the full mitochondrial genome of *Metcalfa pruinosa* reported in Lee et al. (submitted)

**Table S10.** Country-based relative frequencies of the concatenated sequence-based haplotypes (658 bp of the DNA barcoding region and Region 5) for 342 individuals of *Metcalfa pruinosa* collected in Korea and three European countries

| Haplotype | Locality | | | | |
|---|---|---|---|---|---|
| | 1. Korea (297) | 2. Italy (22) | 3. Spain (9) | 4. France (14) | Total |
| 1. MPBR01 | 0.508 (151) | 0.318 (7) | 0.333 (3) | 0.286 (4) | 0.482 (165) |
| 2. MPBR02 | 0.313 (93) | 0.364 (8) | | 0.071 (1) | 0.298 (102) |
| 3. MPBR03 | 0.010 (3) | | | | 0.009 (3) |
| 4. MPBR04 | 0.024 (7) | | | | 0.020 (7) |
| 5. MPBR05 | 0.007 (2) | | 0.556 (5) | | 0.020 (7) |
| 6. MPBR06 | 0.003 (1) | | | | 0.003 (1) |
| 7. MPBR07 | 0.003 (1) | | | | 0.003 (1) |
| 8. MPBR08 | 0.047 (14) | | | 0.071 (1) | 0.044 (15) |
| 9. MPBR09 | 0.010 (3) | | | | 0.009 (3) |
| 10. MPBR10 | 0.003 (1) | | | | 0.003 (1) |
| 11. MPBR11 | 0.003 (1) | | | | 0.003 (1) |
| 12. MPBR12 | | | | 0.071 (1) | 0.003 (1) |
| 13. MPBR13 | | | 0.111 (1) | | 0.003 (1) |
| 14. MPBR14 | 0.007 (2) | | | | 0.006 (2) |
| 15. MPBR15 | 0.013 (4) | | | | 0.012 (4) |
| 16. MPBR16 | 0.027 (8) | 0.318 (7) | | 0.429 (6) | 0.061 (21) |
| 17. MPBR17 | 0.013 (4) | | | | 0.012 (4) |
| 18. MPBR18 | 0.003 (1) | | | | 0.003 (1) |
| 19. MPBR19 | | | | 0.071 (1) | 0.003 (1) |
| 20. MPBR20 | 0.003 (1) | | | | 0.003 (1) |
| Total | 0.868 (297) | 0.064 (22) | 0.026 (9) | 0.041 (14) | 1.000 (342) |

Numbers in parentheses indicate sample size in each country

**Table S11.** Locality-based relative frequencies of the concatenated sequence-based haplotypes (658 bp of the DNA barcoding region and Region 5) for 342 individuals of *Metcalfa pruinosa* collected in Korea and European countries

| Locality | Haplotype | | | | | | | | | | | | | | | | | | | | |
|---|---|---|---|---|---|---|---|---|---|---|---|---|---|---|---|---|---|---|---|---|---|
| | MPBR 01 | MPBR 02 | MPBR 03 | MPBR 04 | MPBR 05 | MPBR 06 | MPBR 07 | MPBR 08 | MPBR 09 | MPBR 10 | MPBR 11 | MPBR 12 | MPBR 13 | MPBR 14 | MPBR 15 | MPBR 16 | MPBR 17 | MPBR 18 | MPBR 19 | MPBR 20 | Total |
| 01 (15) | | 1.00 (15) | | | | | | | | | | | | | | | | | | | 0.04 (15) |
| 02 (18) | 0.33 (6) | | 0.17 (3) | | | 0.06 (1) | | | | | | | | 0.11 (2) | 0.17 (3) | 0.06 (1) | | 0.06 (1) | | 0.06 (1) | 0.05 (18) |
| 03 (18) | 0.67 (12) | 0.06 (1) | | | | | | 0.06 (1) | | | | | | | | | 0.22 (4) | | | | 0.05 (18) |
| 04 (19) | 0.63 (12) | 0.32 (6) | | | | | | | | 0.05 (1) | | | | | | | | | | | 0.06 (19) |
| 05 (22) | 0.96 (21) | | | | | | | | | | | | | | | 0.05 (1) | | | | | 0.06 (22) |
| 06 (21) | 0.05 (1) | 0.33 (7) | | | | | | 0.62 (13) | | | | | | | | | | | | | 0.06 (21) |
| 07 (23) | 0.22 (5) | 0.52 (12) | | 0.17 (4) | | | | | 0.09 (2) | | | | | | | | | | | | 0.07 (23) |
| 08 (18) | 0.17 (3) | 0.72 (13) | | 0.11 (2) | | | | | | | | | | | | | | | | | 0.05 (18) |
| 09 (17) | | 1.00 (17) | | | | | | | | | | | | | | | | | | | 0.05 (17) |
| 10 (21) | 0.33 (7) | 0.57 (12) | | 0.05 (1) | | | 0.05 (1) | | | | | | | | | | | | | | 0.06 (21) |
| 11 (12) | 0.67 (8) | 0.08 (1) | | | 0.08 (1) | | | | | | | | | | | 0.17 (2) | | | | | 0.04 (12) |
| 12 (16) | 0.50 (8) | 0.44 (7) | | | | | | | | | | | | | | 0.06 (1) | | | | | 0.05 (16) |
| 13 (20) | 1.00 (20) | | | | | | | | | | | | | | | | | | | | 0.06 (20) |
| 14 (18) | 0.78 (14) | 0.11 (2) | | | | | | | | | | | | | 0.06 (1) | 0.06 (1) | | | | | 0.05 (18) |
| 15 (20) | 0.95 (19) | | | | | | | | 0.05 (1) | | | | | | | | | | | | 0.06 (20) |
| 16 (19) | 0.79 (15) | | | | 0.05 (1) | | | | | | 0.05 (1) | | | | | 0.11 (2) | | | | | 0.06 (19) |
| 17 (4) | 0.25 (1) | 0.25 (1) | | | | | | | | | | | | | | 0.50 (2) | | | | | 0.01 (4) |
| 18 (3) | 0.67 (2) | | | | | | | | | | | | | | | 0.33 (1) | | | | | 0.01 (3) |
| 19 (10) | 0.40 (4) | 0.30 (3) | | | | | | | | | | | | | | 0.30 (3) | | | | | 0.03 (10) |
| 20 (5) | | 0.80 (4) | | | | | | | | | | | | | | 0.20 (1) | | | | | 0.02 (5) |
| 21 (9) | 0.33 (3) | | | | 0.56 (5) | | | | | | | | 0.11 (1) | | | | | | | | 0.03 (9) |
| 22 (1) | 1.00 (1) | | | | | | | | | | | | | | | | | | | | 0.003 (1) |
| 23 (9) | 0.11 (1) | 0.11 (1) | | | | | | 0.11 (1) | | | | | | | | 0.67 (6) | | | | | 0.03 (9) |
| 24 (4) | 0.50 (2) | | | | | | | | | | | 0.25 (1) | | | | | | | 0.25 (1) | | 0.01 (4) |
| Total | 0.48 (165) | 0.30 (102) | 0.01 (3) | 0.02 (7) | 0.02 (7) | 0.03 (1) | 0.03 (1) | 0.04 (15) | 0.01 (3) | 0.003 (1) | 0.003 (1) | 0.003 (1) | 0.003 (1) | 0.01 (2) | 0.01 (4) | 0.06 (21) | 0.01 (4) | 0.003 (1) | 0.003 (1) | 0.003 (1) | 1.00 (342) |

Numbers in parentheses indicate sample size in each population.

01, Incheon; 02, Yeoju; 03, Pyeongchang; 04, Wonju; 05, Danyang; 06, Goesan; 07, Taean; 08, Yesan; 09, Cheongdo; 10, Sangju; 11, Yeongju; 12, Gimhae; 13, Iksan; 14, Haenam; 15, Jangseong; 16, Suncheon; 17, San Remo; 18, Savona; 19, Borghetto; 20, Genova; 21, Lleida; 22, INRA (The French National Institute for Agricultural Research); 23, Montpellie; and 24, ARS (Ars-sur-Formans). 1–16, Korea; 17–20, Italy; 21, Spain; and 22–24, France
