# Supplementary material for: Tracing the Invasion and Expansion Characteristics of the Flatid Planthopper, Metcalfa pruinosa (Hemiptera: Flatidae), in Korea Using Mitochondrial DNA Sequences
Source: Insects. 2020 Dec 23;12(1):4. doi: 10.3390/insects12010004 (PMC7822484; doi:10.3390/insects12010004)
Supplement: Supplementary file 1 [file insects-12-00004-s001.zip › supplementary-xml/supplementary figures.pdf]

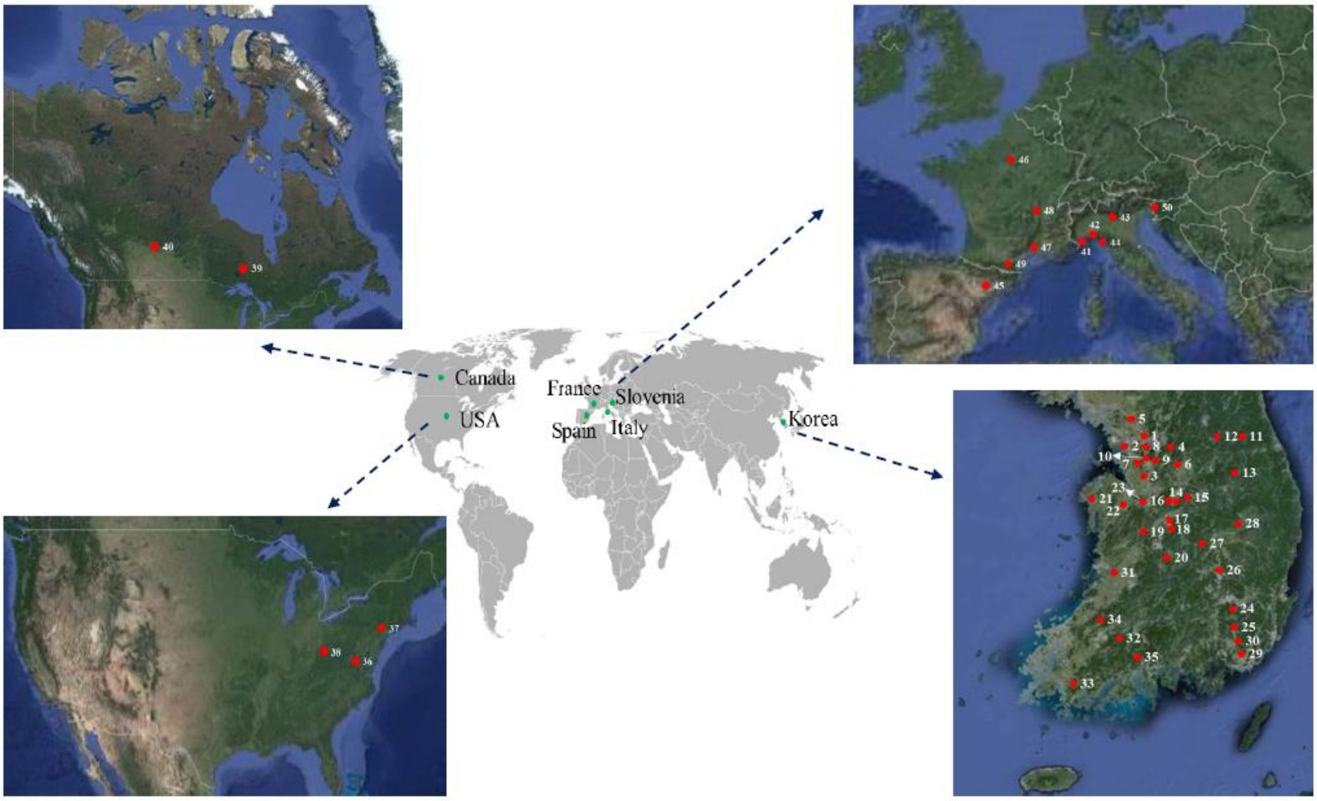

Figure S1. Sampling locations of Metcalfa pruinosa. General locality names are as follows: Korea (1, Seoul; 2, Incheon; 3, Anseong; 4, Yeoju; 5, Paju; 6, Incheon; 7, Hwaseong; 8, Gwacheon; 9, Yongun; 10, Suwon; 11, Pyeongchang; 12, Wonju; 13, Danyang; 14, Goesan; 15, Eumsung; 16, Jincheon; 17, Chungju; 18, Cheongwon; 19, Gongju; 20, Geumsan; 21, Taean; 22, Yesan; 23, Cheonan; 24, Daegu; 25, Cheongdo; 26, Chilgok; 27, Sangju; 28, Yeongju; 29, Gimhae; 30, Miryang; 31, Iksan; 32, Gokseong; 33, Haenam; 34, Jangseong; and 35, Suncheon), USA (36, Maryland; 37, New Jersey; and 38, West Virginia), Canada (39, Ontario; and 40, Saskatchewan), Italy (41, San Remo; 42, Savona; 43, Borghetto; and 44, Genova), Spain (45, Lleida), France [46, INRA (The French National Institute for Agricultural Research); 47, Montpellier; 48, ARS (Ars-sur-Formans); and 49, Le Boulou], and Slovenia (50, Pri Hrastu).

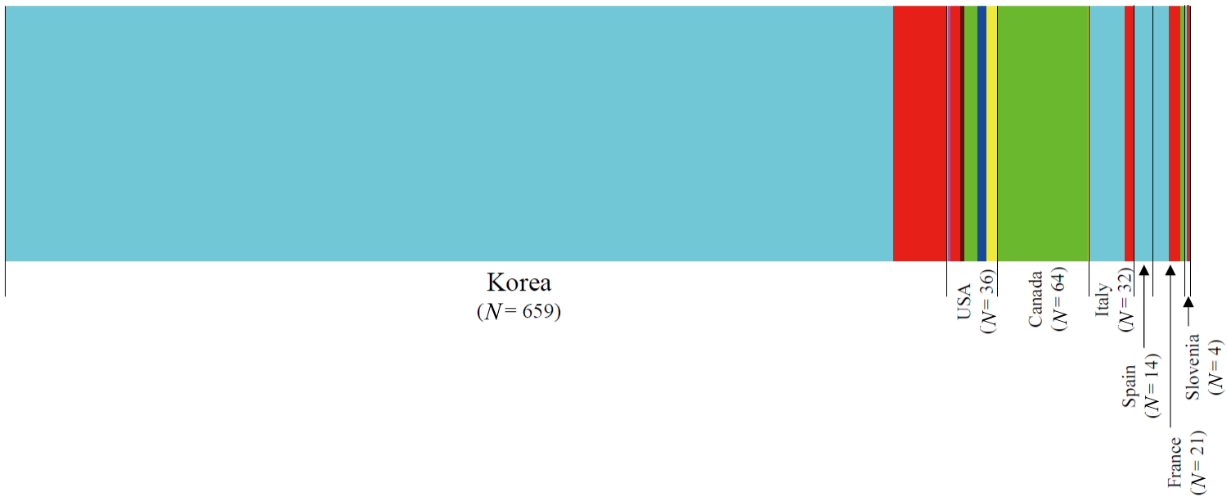

Figure S2. Clustering with Bayesian Analysis of Population Structure (BAPS) of Metcalfa pruinosa based on country using worldwide *COI* data. The optimal number of clusters (K) was seven.
